# Stable clique membership in male mouse societies requires oxytocin-enabled social sensory states

**DOI:** 10.1038/s41467-026-76841-5

**Published:** 2026-08-17

**Authors:** Corentin Nelias, Sarah Ghanayem, David Wolf, Marcel Moor, Danai Nikolantonaki, Eda Dilara Turgut, Max F. Scheller, Valery Grinevich, Jonathan R. Reinwald, Wolfgang Kelsch

**Affiliations:** 1 Department of Psychiatry and Psychotherapy, University Medical Center Mainz, Johannes-Gutenberg University, Mainz, Germany; 2 Department of Psychiatry and Psychotherapy, Central Institute of Mental Health, Medical Faculty Mannheim, Heidelberg University, Mannheim, Germany; 3 Department Neuropeptide Research in Psychiatry, Central Institute of Mental Health, Medical Faculty Mannheim, Heidelberg University, Mannheim, Germany

**Keywords:** Social behaviour, Sensory processing, Social neuroscience

## Abstract

The ability to establish stable de novo relationships in complex environments is essential for social functioning but disrupted in disorders such as autism. Yet, the mechanisms supporting higher-order bonding in large groups remain unclear. Here, we introduce a naturalistic model of clique formation in mouse societies, using longitudinal tracking from large-scale video data. We show that small, stable cliques emerged from the specific group configurations. Consistently, also kinship did not significantly facilitate entry. These cohesive cliques resembled human mutually interacting rich-clubs, exhibiting high social rank and influence over non-members. We next examined whether oxytocin signaling in the olfactory cortex supports such higher-order bonding. Despite preserved social motivation, mice with conditional oxytocin receptor deletion in this sensory cortex failed to join rich-clubs, approached peers less consistently, and received unstable reciprocal connections. This network-level disorganization demonstrates how social dynamics can magnify individual deficits. Our findings identify oxytocin-dependent social sensory states as a necessary mechanism for forming stable relationships in complex social networks.

## Abstract (web summary)

Oxytocin sets sensory cortex into a social processing state, but how this shapes higher-order relationships is unclear. Here, authors show it is required to join stable cliques: without it, mice approach peers erratically, and peers respond likewise.

## Introduction

Structured social networks emerge from repeated interactions, leading to preferential associations that may stabilize into enduring affiliative subgroups^1^. The ability to form and maintain such de novo social relationships also varies substantially across the general population^2–4^. More severe difficulties in establishing stable interpersonal relationships are a hallmark of severe mental disorders and significantly contribute to impairments in everyday functioning^2,5–7^. Importantly, such variations in higher-order social behavior may not necessarily manifest as reduced social interest or interaction frequency^2,7^. Instead, they often reflect a diminished ability to occupy and maintain functional roles within complex social networks^2,4^. High-functioning autism spectrum disorder (ASD), often conceptualized as an extreme expression along a continuum of social functioning^2,3^, exemplifies the dissociation between social motivation and success in building new stable relationships across various contexts^2,3,8–10^. Symptoms may, however, not fully manifest until the increasing complexity and flexibility in social demands exceed limited capacities^11^.

Network theory provides a powerful framework for understanding the structural underpinnings of social cohesion. It highlights the critical role of tightly connected subgroups in maintaining network resilience and integrity^8,12,13^. While such structures are well documented in real-world and digital social networks^2,8,12–14^, the neurobiological mechanisms that support their formation and maintenance remain poorly understood. In graph theory, highly integrated social configurations can be described as rich-clubs (RC), which are densely interconnected nodes within the networks^8,14^. These RCs support robust social functioning and exert significant influence over information flow, social support, and integration^8,12,14,15^. Related clique structures exist in other social species, such as coalitions in chimpanzees, social clusters in dolphins, or relationships in ravens^16–18^. Identifying how individuals gain stable access to these social cliques may provide insight into the subtle cognitive and neural mechanisms regulating social integration.

Social networks are non-linear dynamical systems^8,14^. This has several implications. Certain phenotypes may be amplified through emergent properties of higher-order social interactions involving multiple individuals^8,19^. Emergent properties are macro-level phenomena that result from micro-level interactions and cannot be reduced to the sum of their parts. Thus, a change in one individual (or ‘node’) may not only alter its own behavior but also modify the responses of other nodes, leading to dynamic feedback effects^8,14,15^. We adopt the definition of RCs from network science and use it here strictly as a connectivity-based term, i.e., a topological property describing preferential, reciprocal connectivity among highly connected individuals. Based on this formal description, we can then test whether membership in a RC is associated with other dominance-related behaviors.

In mammals, oxytocin is a key modulator of social behavior^20–24^ and is investigated as a therapeutic agent for disorders characterized by social deficits^25,26^. However, clinical trials of intranasal oxytocin have yielded inconsistent and often disappointing outcomes in socially complex settings^25,26^. This discrepancy raises the question of whether our mechanistic understanding of oxytocin function is sufficient^27^, particularly in the context of higher-order social behaviors.

Oxytocin modulates diverse region-specific brain functions, including social sensory processing, motivation, bonding, and aggression^20–23,28^. Neuropsychological studies have identified impaired social sensory processing and recognition in the auditory, olfactory and visual domains in ASD^29–32^. Oxytocin sets the olfactory system of rodents into a state optimized for processing social information^22,24^. Oxytocin receptor (OXTR) activation in the anterior olfactory nucleus (AON) increases the signal-to-noise of sensory input^22^. Mice with reduced OXTR signaling in this sensory cortex (OXTR^∆AON^) are impaired in social recognition at the neuronal and cognitive level^24^. Similarly, human sensory processing is modulated by genetic polymorphisms influencing OXTR expression^33,34^.

It remains unclear what phenotypic consequences oxytocin’s function in sensory state setting has for higher social functioning in real-life conditions. Several scenarios are conceivable. For example, impaired sensory state setting may selectively affect short-term social recognition. Sensory state modulation may additionally influence social motivation. Alternatively, sensory deficits may be fully compensated in complex environments over time. It is, however, also possible that these subtle sensory deficits become more relevant with increasing complexity, when individuals must learn structured navigation in social networks de novo. The latter scenario would mean that early sensory alterations can cause a phenotype that is typically associated with higher cognitive functions.

To answer these questions, individuals must be observed in groups and over longer periods of time. Groups need to be of sufficient size to allow subnetworks to form, stabilize, or dissolve, and to avoid despotic hierarchies typical of smaller groups^35^. Further, animals should be exposed to groups composed of different individuals to disentangle whether internalized prior experience or the specific group context promotes RC membership. Moreover, multiple domains of social behavior should be captured to put social relations into a comprehensive perspective. Finally, inspired by natural patterns of genetic variation within populations^36^, genetic manipulations should be limited to a minority of animals to ensure that neurotypical individuals continue to shape the group dynamics.

To meet these requirements, we designed a non-invasive, sensor-rich platform (NoSeMaze) for mice^37^, which continuously monitors spontaneous social behavior of identified individuals in a larger group over weeks. The sensor-rich system, equipped with video and wireless identity tracking, enables automated monitoring of naturalistic interactions and their network dynamics without experimenter intervention. Leveraging the NoSeMaze, we begin here to dissect the factors and mechanisms underlying the formation and stabilization of RC structures. To test how the reduced oxytocin signaling in the olfactory cortex affects complex social behavior, we interspersed a minority of mice carrying OXTR^∆AON^ into otherwise neurotypical groups.

## Results

### OXTR deletion in the AON does not alter brief dyadic social interaction patterns

**Fig. 1:**
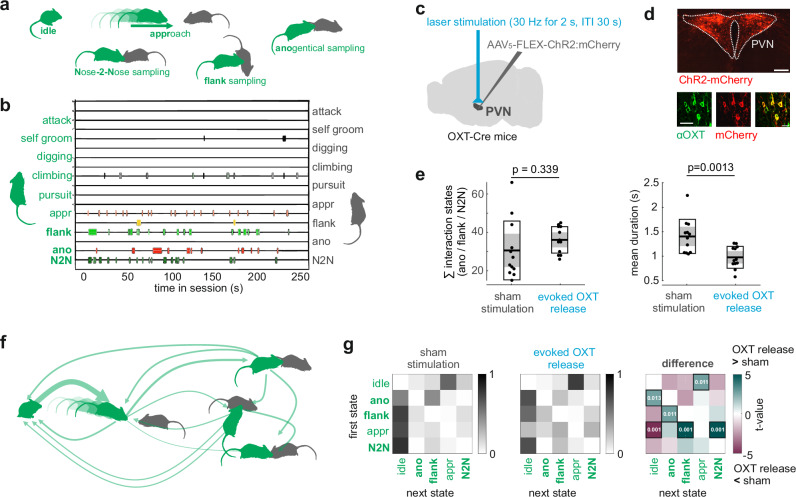
Boosted global OXT release modifies social exploration patterns. **a** Ethogram defining key behavioral states observed during self-paced dyadic interactions, including idle (pause), approach, and various forms of social sniffing. The experimental mouse (adult) is illustrated in green and the interaction partner in gray. **b** Example session illustrating the temporal sequence of behavioral states across different interaction categories. **c** Schematic of optogenetic stimulation. **d** Virus injection in the paraventricular nucleus of OXT-Cre mice for conditional expression of ChR2-mCherry in OXT-immunoreactive neurons. **e** Even though global optogenetic enhancement of OXT release did not significantly increase the number of sniffing events (sum of flank, anogenital, and nose-to-nose; left), it significantly reduced the duration of events (right). (linear mixed effects model with random effects on group and animal identity, see methods). Black lines indicate the group mean, gray shaded areas the 95% confidence intervals of the mean and white boxes the standard deviation. **f** Diagram illustrating transitions between behavioral states. Arrows indicate the direction and frequency of transitions. **g** Transition matrices for behavioral state of sham vs. evoked OXT release. Right panel: paired difference matrix (*t* values) reveals increased transitions from approach into social sniffing events with boosted OXT release. *P* values are FDR-corrected for multiple comparisons. Source data are provided as a Source Data file. AON anterior olfactory nucleus, PVN paraventricular nucleus.

It is not entirely known if loss of OXT signaling in the AON affects the microstructure of self-paced dyadic same-sex interactions. We therefore first analyzed video-tracked interaction states and the transitions in a simple condition of brief explorations of two mice in a cage suited for high-resolution mapping of exploration sequences (Fig. 1a, b). Specifically, we quantified how often an animal progressed from approach (orientation and movement toward the partner within close range) to sniffing (close-contact investigatory olfactory behavior directed to the partner, including anogenital, nose-to-nose, and flank-sniffing), versus disengaging after initiating approach (i.e., aborted interactions). To probe the sensitivity of the isolated dyadic assay, we first tested mice with optogenetics-boosted global OXT release throughout the forebrain (AAV-mediated conditional ChR2 expression in the paraventricular nucleus (PVN) of male OXT-Cre mice) or sham stimulation in the same animals (Fig. 1c, d). Boosted OXT release led to a tendency toward more sniffing events, with significantly shorter durations (Fig. 1e). Boosted OXT release also increased the likelihood that approaches progressed into sniffing and reduced the probability that interactions were aborted after an approach was initiated (Fig. 1f, g), consistent with enhanced social exploration.

**Fig. 2:**
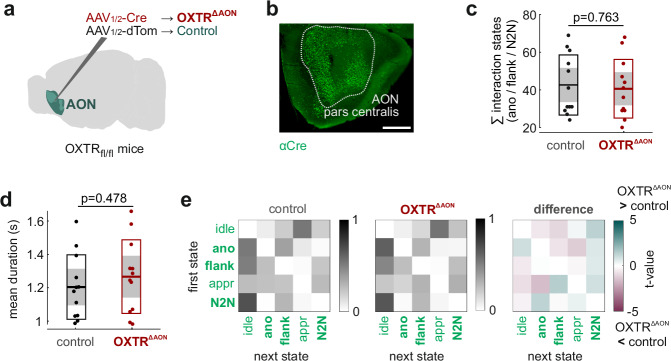
Social microstructure is preserved in OXTR^ΔAON^ mice during brief dyadic explorations. **a** Schematic of selective OXTR deletion in the AON of adult OXTR_fl/fl_ mice via AAV_1/2_-Cre injection. **b** Cre immunoreactivity shown in the AON pars centralis (right). **c**, **d** Mice with selective OXTR^ΔAON^ deletion differed neither in **c** the number nor **d** the duration of sampling events from matched controls (linear mixed effects model with random effects on group and animal identity, see methods). Black lines indicate the group mean, gray shaded areas the 95% confidence intervals of the mean and white boxes the standard deviation. **e** Transition matrices for control vs. OXTR^ΔAON^ mice show no significant differences in state transitions. Together, these findings indicate that deletion of OXTR in the AON does not disrupt the microstructure or progression of short dyadic social behaviors, supporting the idea that this region contributes more to social sensory processing rather than the initiation of social action patterns. Source data are provided as a Source Data file. AON anterior olfactory nucleus, OXTR oxytocin receptor.

In the same conditions, we next examined a group of mice with selective adult deletion of OXTR in the AON (OXTR^ΔAON^ with bilateral injection of AAV_1/2_-CBA-Cre in adult male OXTR_fl/fl_ mice) and their matched controls (Fig. 2a, b). In contrast to boosted OXT release, OXTR^ΔAON^ mice did not differ from controls in the number or duration of interaction states (Fig. 2c, d). Further, OXTR^ΔAON^ mice showed no significant differences from matched controls in transition probabilities (Fig. 2e). Partner behavior remained unchanged across all groups (Supplementary Fig. 1), indicating no reactive changes in partners’ behavior.

In summary, selective deletion of OXTR in the AON did not alter dyadic social interaction behavior during brief interactions, consistent with OXT in this cortex acting mainly on social cognition. We therefore asked what consequences this selective impairment in social cognition may have in complex and dynamic social networks.

### OXTR^ΔAON^ mice display quantitatively normal social activity in groups

**Fig. 3:**
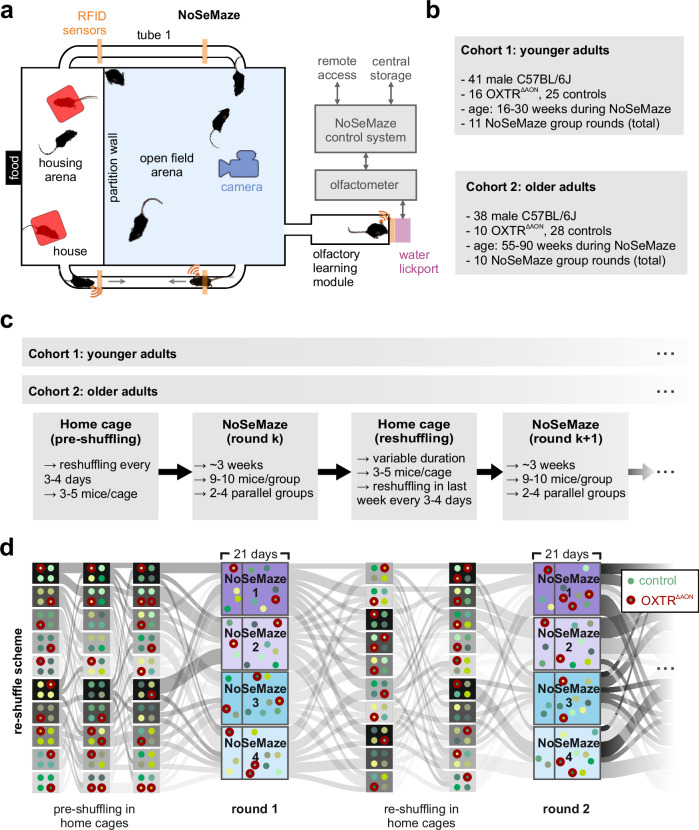
Automated tracking in the NoSeMaze of individuals’ behaviors in groups with interspersed OXTR^ΔAON^ mice. **a** The NoSeMaze combines ceiling-mounted video surveillance and RFID sensors to continuously track the social behaviors and reinforcement learning of mouse groups (*n* = 9–10 mice per group) without human interference. Mice are identified via RFID-tagged implants at access points in the tubes and the water lickport. In the open-field arena, mice are tracked by video to reconstruct social interaction networks from identified individuals. **b** Overview of the two adult cohorts studied in separate experimental series. **c** Experimental timeline. Before entry into the NoSeMaze, home-cage compositions were repeatedly reshuffled every 3–4 days (3–5 mice per cage) to promote familiarity among cohort members. Mice were then housed in the NoSeMaze for 3 weeks per round in groups of 9–10, with 2–4 groups run in parallel. Between consecutive NoSeMaze rounds, mice returned to reshuffled home cages, where they were again reshuffled in the last week before the next NoSeMaze round. Reshuffling occurred within cohorts only, not across cohorts. **d** Sankey-style diagram exemplarily illustrating how mice were reassigned across successive NoSeMaze rounds with reshuffling periods in home cages to ensure broad familiarity both before and between rounds. Each group consisted of 9–10 individuals with 2–5 OXTR^ΔAON^ mice (red circle). Reshuffling enabled dissociation of effects emerging from internalized traits in individuals versus group composition context. NoSeMaze non-invasive sensor-rich maze, RFID radio-frequency identification.

To assess social interaction dynamics in a complex setting while retaining full tracking of individual behavior, we employed the NoSeMaze system^37^. The NoSeMaze provides a sensor-rich environment enabling long-term, automated tracking of cognitive and social behaviors in group-housed mice without human interference (Fig. 3a). We monitored groups of 9–10 RFID-chipped male mice (79 total; 26 OXTR^ΔAON^, 53 controls) on a C57BL/6 J background over continuous three-week rounds in the NoSeMaze. Each group included two to five OXTR^ΔAON^ mice interspersed among controls to mimic sparse genetic variation. To ensure that phenotypes were observed across the adult lifespan, we studied two age cohorts separately: younger (16–30 weeks; 41 mice, 16 OXTR^ΔAON^, 25 controls) and older adults (55–97 weeks; 38 mice, 10 OXTR^ΔAON^, 28 controls) (Fig. 3b). Before entering the NoSeMaze, mice within each cohort were repeatedly reshuffled in home-cage groups of 3–5 animals (Fig. 3c, d) to promote familiarity among future group members. This procedure may also have contributed to the lack of overt aggression or injuries in the NoSeMaze. Most mice participated in multiple rounds (see Supplementary Table S1). To assess whether social behaviors depended on specific group composition, mice lived with different group members in each round of the NoSeMaze (‘reshuffling’, Fig. 3d).

**Fig. 4:**
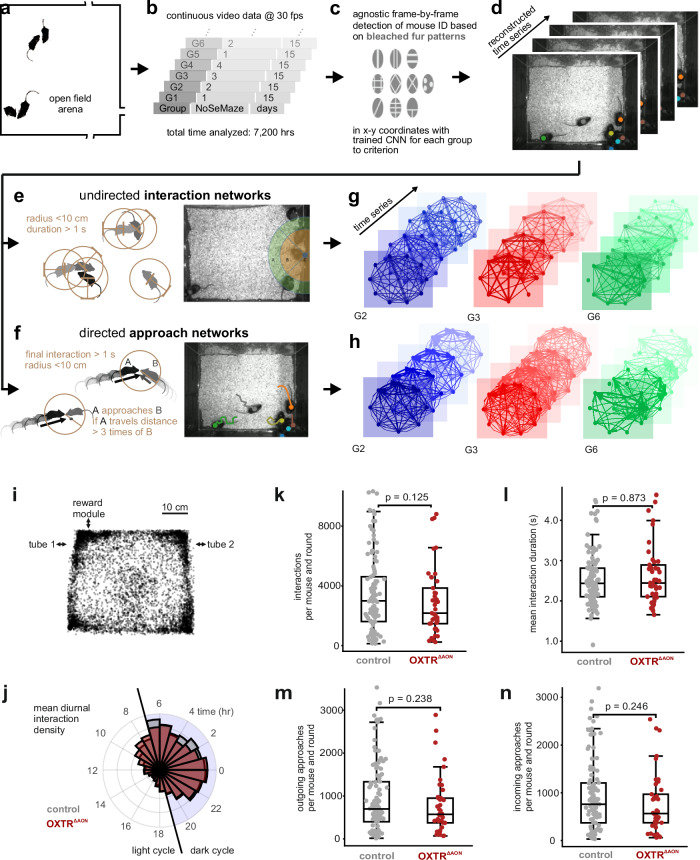
Long-term video tracking reveals comparable levels of social interactions in OXTR^ΔAON^ and control mice. **a** Schematic of the open-field arena in the NoSeMaze, where mice interacted freely. A ceiling-mounted camera continuously recorded video data. **b** The first two weeks were analyzed for each group. **c**, **d** Mouse identity and position were extracted frame-by-frame using convolutional neural networks (CNNs) trained on the bleached individual fur patterns on the animals’ backs (see Methods). The CNN output was smoothed to reconstruct individual trajectories over time, yielding a final data set of ~7200 hours of open-field observation. **e** Social interactions were defined as events in which two mice had a distance <10 cm for at least 1 s. **f** Directed approaches were identified as a subset of interaction events where one mouse (A) approached another (B) by traveling at least three times the distance of B in the 4 s preceding their interaction. **g**, **h** From these two event types, we constructed time-resolved social networks by aggregating interactions for each 24-h period (see Methods). Interaction networks were undirected, as no distinction was made between the two mice involved (**g**). In contrast, approach networks were directed, leveraging the direction of approach (**h**). **i** Spatial distribution of interaction events in G9 over a round. Each dot represents one interaction. **j** Diurnal distribution of interactions. Mice were most active at night. Overlapping interaction activity was observed for OXTR^ΔAON^ (red) and control (gray) mice. **k**–**n** Social behavioral metrics across the first 15 days of each round. Each dot represents a single animal per round. No significant differences were observed between OXTR^ΔAON^ (red) and controls (gray) in **k** total number of interactions, or **l** average interaction duration (seconds), or **m** the number of initiated approaches and **n** received approaches (two-sided permutation test on the median, 10,000 iterations). In the boxplots, the thick central line shows the median. The lower and upper bounds of the box indicate the 25th and 75th percentiles (Q1 and Q3). The whiskers extend from the box to the farthest data point lying within 1.5× the interquartile range (IQR) from the box. Source data are provided as a Source Data file.

**Fig. 5:**
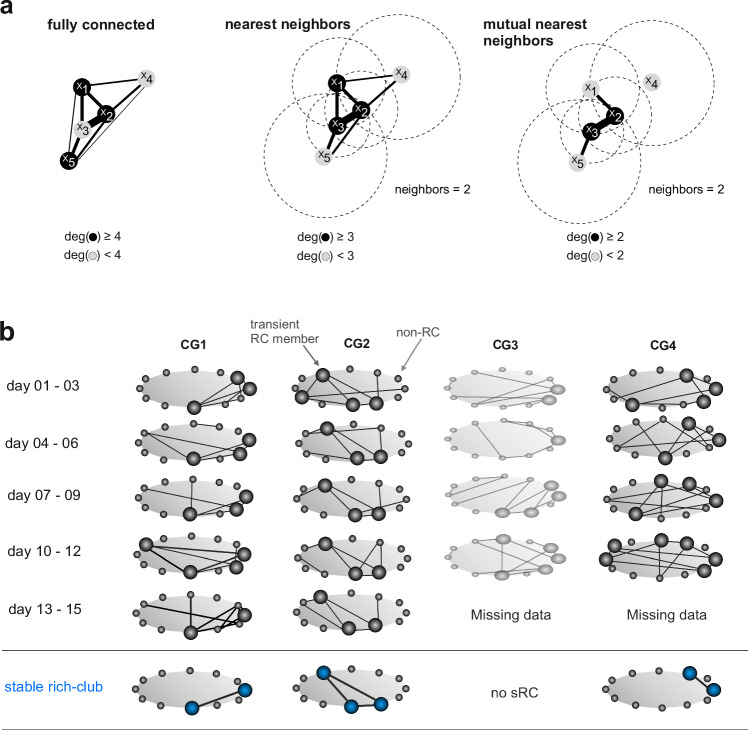
Stable rich clubs (sRCs) emerge in NoSeMaze groups. **a** Schematic illustrating the mutual nearest neighbors graph-cut procedure used to identify RCs. Starting from a fully connected graph (left), edges are filtered based on 2nd-order nearest neighbors (middle), and further reduced to a final graph (right) that preserves edges only if nodes are mutual 2nd-order nearest neighbors of each other. Dashed circles indicate each node’s 2nd-nearest neighbor radius; nodes within each other’s circle form mutual connections. Nodes forming RCs are shown in black; others in gray. As the graph-cut criterion becomes more selective, the number of RC members decreases. **b** RC dynamics across four control rounds (CG1, CG2, CG3 and CG4) over time. This additional cohort only contained male wild-type mice of the same strain as the main cohort. Nodes in ‘stable RCs’ (sRCs), defined as maintaining RC-membership across at least 80% of 3-day time windows, are shown in bold blue; transient members in bold gray. Stability is reflected in the consistent preservation of RC structure over time. Light transparency was applied to indicate the absence of sRC in a round (CG3).

We then established a pipeline to extract the social interaction networks from continuous video data in the open-field arena for the groups of individually identified mice, including interspersed OXTR^ΔAON^ mutants (Fig. 4a, b). To identify individual mice, distinct fur patterns were bleached onto the back of the mice before each round (Fig. 4c). We analyzed the first 15 days of each round, as the subsequent regrowth of the fur compromised the reliability of pattern recognition by the trained Convolutional Neural Network (CNN). Before each new round, the fur patterns were refreshed. 16 out of 21 NoSeMaze rounds with interspersed OXTR^ΔAON^ mice yielded video datasets of sufficient quality for social network reconstruction and were included in the video-based analyses (Fig. 4a, b). Additionally, we analyzed four rounds with wild-type mice only (Fig. 5b), totaling overall to 7200 hours of continuous video data used to generate the social networks in the final dataset (Fig. 4d).

The CNN-based pipeline allowed us to extract spatial coordinates for each animal as a function of time. From these tracked positions, we defined and extracted two types of social events: interactions and approaches. This enabled us to represent the data as a social network. Interactions were counted whenever two animals were within <10 cm for at least 1.0 s (Fig. 4e). Approaches were defined using two criteria: Firstly, an approach had to end with an interaction as defined above. This is to ensure that behaviors such as following or chasing are not included. Secondly, to ensure directionality in the approach, the distance traveled by the ‘approaching’ mouse in the 4 s before the interaction had to be at least three times the distance traveled by the other mouse (Fig. 4f). The threshold was chosen based on a sensitivity analysis (Supplementary Figs. 3 and 4). Events below this relative directionality threshold were excluded from directed-approach analyses. This criterion was chosen to ensure that approach events had a clear direction while retaining a sufficient number of events for the analysis (see Methods). Mutual approaches were therefore excluded, as the relative distances traveled by both interaction partners would be comparable and thus fail to meet the predefined asymmetries in approach. From these measures, we obtained time series of undirected and directed networks (Fig. 4g, h, see Methods).

Spatially, interactions occurred preferentially near the walls (Fig. 4i, Supplementary Fig. 2) and predominantly during the active dark cycle (Fig. 4j). Notably, OXTR^ΔAON^ and control mice displayed quantitatively similar numbers and durations of interactions (Fig. 4k, l), as well as comparable numbers of both incoming and outgoing approaches (Fig. 4m, n), suggesting preserved approach behavior in OXTR^ΔAON^ mice (for further results see Supplementary Fig. 5). We therefore tested the hypothesis that the social cognition deficits caused by OXTR^ΔAON^ specifically impair the formation of targeted and consistent social relationships.

### Stable RCs emerge in social networks of group-housed mice

While social relationships can be unstable or occur by chance, they can also gain stability among some animals in the same habitat. We were particularly interested in identifying the key factors that enable the formation of such stable reciprocal cliques within larger groups.

Interaction data were analyzed across five consecutive three-day windows per round. Given the high connectivity in the networks, graphs were pruned using a mutual nearest-neighbor approach to isolate behaviorally relevant substructures (Fig. 5a). These structures correspond to RCs^15,22^. We defined RC membership based on node degree in the pruned interaction network. Nodes with degree ${{\rm{d}}}\ge 3$ were classified as RC members. Prior to computing degree, we pruned weak connections by retaining, for each node, edges to its mutual k-nearest neighbors (*k* = 3; see Methods).

Before analyzing the main cohort containing both controls and OXTR^ΔAON^ mice, we first asked whether RCs were expected to emerge in pure groups of wild-type mice of the same strain as the main cohort (see Supplementary Table S4). To address this, we analyzed four independent rounds consisting exclusively of younger adult male wild-type mice. We were particularly interested if stable rich-clubs (sRC) emerged in these groups. RCs were considered stable if they persisted across at least four out of five three-day-graphs. Such sRCs were identified in three of four groups (Fig. 5b).

**Fig. 6:**
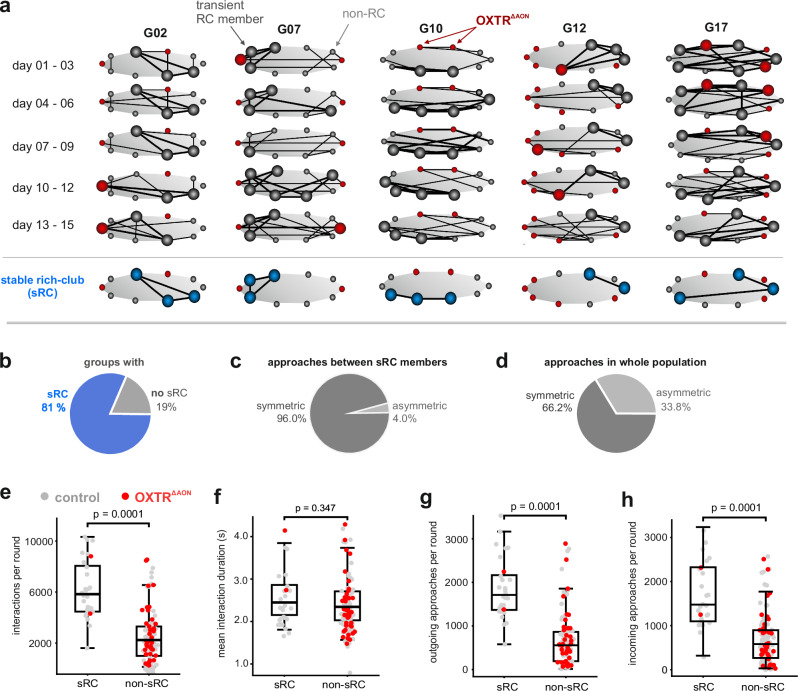
Stable rich clubs (sRCs) are characterized by high approach density. **a** Representative examples of RC dynamics across five rounds (G02, G07, G10, G12, and G17) over time. Nodes in ‘stable RCs’ (sRCs), defined as maintaining RC-membership across at least 80% of 3-day time windows, are shown in bold blue; transient members in bold gray. OXTR^ΔAON^ mice are indicated in red. Stability is reflected in the consistent preservation of RC structure over time. **b** Stable RCs were detected in 81% of the 16 NoSeMaze rounds that were available for analysis. **c** Symmetry of approach behavior of sRC members compared to **d** the rest of the population (bottom). Even more than when examining all mice, sRC members engaged in symmetric approaches among themselves. **e**–**h** Quantification of social activity of sRC members and non-sRC members. Gray dots indicate control mice and red dots OXTR^ΔAON^ mice. Each dot represents one animal in one round. sRC members had more **e** interactions **g** outgoing approaches, as well as **h** incoming approaches per round, than non-members, while **f** average interaction duration (in seconds) was not significantly different between the two groups. Statistical significance was assessed via a two-sided permutation test on the median, 10,000 iterations. In the boxplots, the thick central line shows the median. The lower and upper bounds of the box indicate the 25th and 75th percentiles (Q1 and Q3). The whiskers extend from the box to the farthest data point lying within 1.5× the interquartile range (IQR) from the box. Source data are provided as a Source Data file.

We then examined the main cohort with interspersed OXTR^ΔAON^ mice (Fig. 6a, Supplementary Fig. 6). In most groups (81%), we observed sRCs. sRCs emerged in all 10 rounds with older adults and in four out of seven rounds in younger adults (Fig. 6b). Probabilistic modeling confirmed that such persistence was highly unlikely to occur by chance (*p* < 10⁻⁷ for pairs; *p* < 10⁻⁹ for triads), indicating genuine social structure. This finding was further supported by richness coefficients consistently exceeding random chance throughout the experiment (see Supplementary Fig. 7). Finally, the overall structures of sRCs were stable when varying the pruning criterion (see Supplementary Fig. 8).

Notably, sRCs were robustly identifiable in both undirected interaction and directed-approach networks (Supplementary Fig. 9). sRC members displayed distinct interaction patterns. sRC members exhibited greater reciprocity: 96.0% of their approaches were reciprocal (Fig. 6c), compared to 66.2% in the whole population (Fig. 6d). Compared to non-sRC-members, they engaged in more interactions and approaches, although mean interaction duration did not differ (Fig. 6e–h). Notably, OXTR^ΔAON^ mice behaved similarly to non-sRC-member control mice on these measures, as similar results were obtained with the different measures (Supplementary Fig. 10 and Supplementary Table S3). Taken together, the formation of sRCs is a typical feature in the habitat and can be identified both from undirected interaction graphs and directional approach networks.

### Rich-club membership emerges from group-specific dynamics

**Fig. 7:**
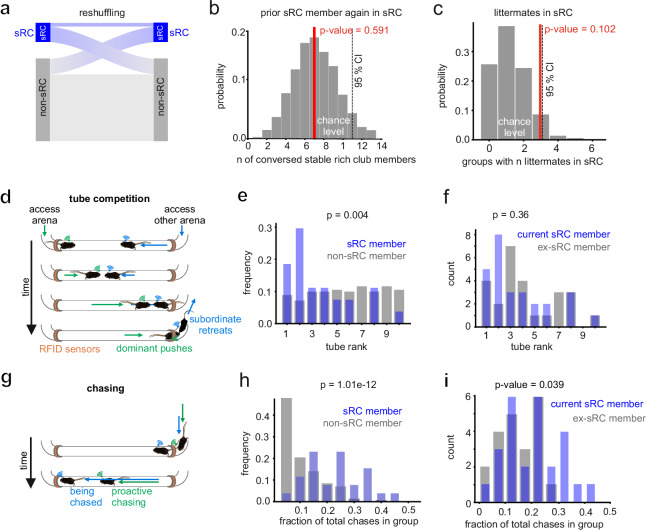
Stable RC membership is shaped by group context and associated with dominance and chasing behavior. **a** Sankey diagram illustrating sRC membership across successive NoSeMaze rounds. The lack of continuity across rounds suggests that sRC membership is not a stable individual trait but group-context dependent. **b** Probability that former sRC members rejoined an sRC in a different round. The observed number (red line) falls within the null model’s 95% confidence interval, indicating that sRC membership is not an internalized individual characteristic. **c** Observed frequency of littermates within the same sRC compared to a null distribution generated by random assignment. The observed frequency (red line) falls within the 95% confidence interval of the null model, indicating that shared family background does not significantly influence sRC formation. **d** Schematic of the tube test used to assess social rank via RFID-tagged outcomes of dyadic competitions. **e** sRC membership was significantly associated with higher social ranks (two-sided *t* test), but was not confined to top-ranking individuals. High rank favors, but does not guarantee sRC membership. **f** Likewise, ex-sRC members often retained high ranks even when not in an sRC (two-sided *t* test). Tube-test ranks of mice that were sRC members in the current round (blue) and the same individuals in rounds when not in the sRC (ex-sRC members, gray). **g** Schematic of chasing events detected in the tubes. **h** sRC members were responsible for a significantly larger fraction of the total initiated chases compared to non-sRC members (two-sided *t* test). **i** Fraction of total chases initiated by current sRC members (blue) and the same individuals when not in the sRC (ex-sRC members, gray). sRC members showed significantly higher chasing activity while in the sRC than when they were not part of the sRC (*p* = 0.039, two-sided permutation test on the mean, 10,000 iterations). High chasing activity may facilitate sRC membership without being a necessary trait. Source data are provided as a Source Data file.

We next examined potential predictors of sRC membership. Firstly, prior membership experience might promote sRC membership in a subsequent round. Alternatively, sRCs may form de novo and be driven by the specific group dynamics. To test this, we leveraged the reshuffling procedure, which generated different group compositions across consecutive NoSeMaze rounds (Fig. 7a). Among the mice participating in multiple NoSeMaze rounds, the probability of re-entering an sRC was at chance level (Fig. 7b). This indicates that sRC membership emerges de novo from the specific social dynamics within each group. These findings were robust and held across a range of graph-pruning parameters (see Supplementary Fig. 11a).

Secondly, because sRCs emerge from the interplay among specific individuals, shared upbringing could promote shared sRC membership in adulthood. We took advantage of having multiple siblings per group to test this possibility. Kinship had a weak trend, but no significant effect on increasing the likelihood of shared sRC membership beyond random chance (Fig. 7c, Supplementary Fig. 11b).

Membership in sRCs was associated with differences in other social behaviors. Using incidental competitions in the integrated tube test of the NoSeMaze, we quantified ranks in the social hierarchy (Fig. 7d). Non-members had a relatively uniform rank distribution, with no significant deviation from uniformity (*χ*² goodness-of-fit test, *p* = 0.11; Fig. 7e). Importantly, sRC members occupied higher social ranks compared to non-sRC-members (Fig. 7e). Consistent with social rank being largely internalized and relatively stable across different groups^37^, the distribution of social ranks of mice that were in the sRC during one NoSeMaze round did not significantly differ from the rank distribution in other rounds when they were not in an sRC (Fig. 7f). Together, this suggests that higher ranks facilitate, but are not sufficient for sRC entry.

We also tested for proactive chasing of others through the tubes (Fig. 7g). sRC-members initiated significantly more chases than non-sRC-members (Fig. 7h). Mice that were in the sRC during one NoSeMaze round contribute more to chasing than in rounds when they were not part of the sRC (Fig. 7i, see also Supplementary Fig. 12b and Supplementary Fig. 13). The sRC-members initiated chasing toward both fellow clique members and non-members (Supplementary Fig. 12e). The behavior of OXTR^ΔAON^ mice in chasing and social rank resembled largely those of non-sRC-member control mice (Supplementary Fig. 12a, c, d).

In summary, most groups in the NoSeMaze formed stable interconnected social structures that can be described as sRCs. We found that mice in the sRCs were significantly more active, engaged in more reciprocal interactions, and initiated more chases than non-members. Because family relationships did not significantly influence rich-club formation, and most mice that entered a clique did not rejoin it with new peers, we conclude that sRC formation is an emergent property of the network, reflecting the dynamic nature of social interactions within each group.

### OXTR^ΔAON^ mice fail to integrate into stable RCs

Since the formation of RC in colonies appears to be a consequence of higher-order social interactions, a crucial question is whether OXTR^ΔAON^ mice enter them or not. OXTR^ΔAON^ mice have been shown to exhibit social recognition deficits^22,24^. While these deficits did not significantly alter the level to which mice engaged in dyadic interactions (cf. Fig. 4k–n), it is nonetheless possible that OXTR^ΔAON^ mice lack the capacity to engage in complex stable social relationships.

**Fig. 8:**
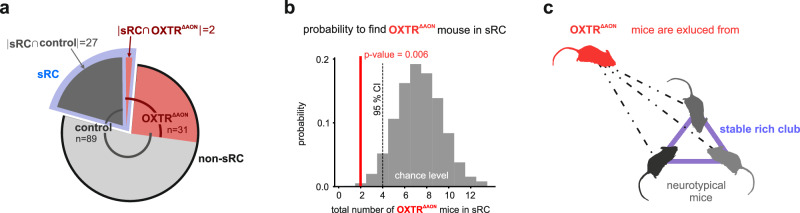
OXTR^ΔAON^ mice fail to integrate into stable rich clubs. **a** Pie chart illustrating the participation of OXTR^ΔAON^ or control mice in the sRCs across all 12 rounds where a stable rich-club was identified. The n indicates the respective mouse-rounds. **b** Observed frequency of OXTR^ΔAON^ mice in sRCs compared to a null distribution generated by random assignment. The observed frequency (red line) was significantly lower than expected by chance (*p* = 0.006, null model testing, implementation details are given in the methods), indicating that OXTR^ΔAON^ mice fail to integrate into sRCs. **c** OXTR^ΔAON^ mice are excluded from stable rich clubs.

Indeed, across all groups, only two OXTR^ΔAON^ mice entered the sRC, a frequency (6.4%) considerably below the fraction observed in control mice (30.3%) (Fig. 8a). We also compared the number of OXTR^ΔAON^ mice that were able to enter the sRC with the number expected by random chance, using a permutation test. The results (Fig. 8b, Supplementary Fig. 14) show that OXTR^ΔAON^ mice were significantly less likely to enter sRCs than controls. This impairment was observed in both younger and older adults (cf. Fig. 6b, Supplementary Fig. 6). In summary, OXT signaling in the AON plays a crucial role in the de novo formation of social cliques within larger mouse groups (Fig. 8c). This contrasts with internalized social ranks and the propensity to chase that are also largely independent of this OXT-mediated olfactory function.

### Unstable interaction dynamics of OXTR^ΔAON^ mice hinder social integration

Although OXTR^ΔAON^ and control mice did not differ in average measures of interaction time, approach frequency, or social rank (c.f. Fig. 4), OXTR^ΔAON^ mice failed to join sRCs (cf. Fig. 8). Building on this finding, we asked whether this deficit extends to group-network dynamics, where fluctuating social connections over time might prevent OXTR^ΔAON^ mice from forming and maintaining higher-order social relationships. In other words, even if overall levels of social activity were normal, their ties may have been too inconsistent or erratic to consolidate into stable cliques.

To address this, we examined the temporal fluctuations of directed-approach networks. To account for potential confounds from current sRC membership, we compared OXTR^ΔAON^ mice to control non-sRC-members (who could still be sRC members in other rounds), thereby isolating network features specific to the OXTR^ΔAON^ phenotype. At the global level, the overall strength of outgoing approaches fluctuated to a similar degree in OXTR^ΔAON^ mice and control non-members (Supplementary Fig. 15), indicating that OXTR^ΔAON^ mice preserved social motivation over time.

**Fig. 9:**
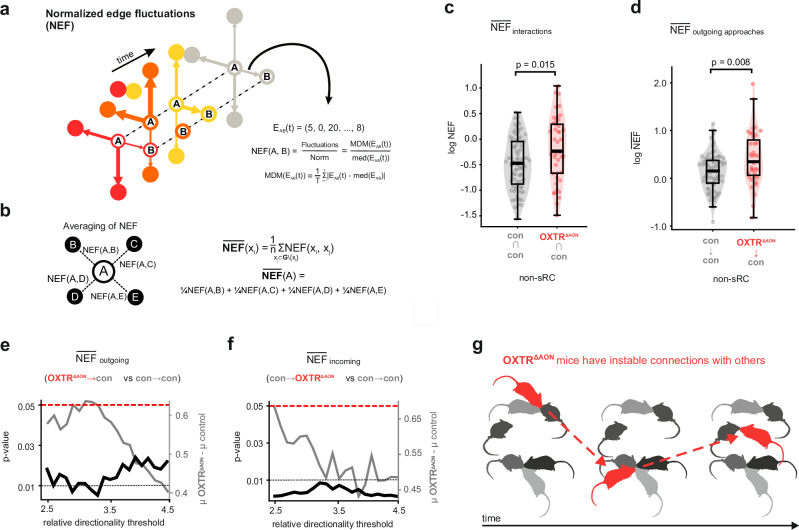
OXTR^ΔAON^ mice display fluctuations in their approach pattern and receive less consistent input from their peers. **a** Schematic illustrating the normalized edge fluctuations (NEFs), defined for each edge as the coefficient of variation (σ/µ) of its time series. **b** Illustration of the procedure to obtain node-level averaged NEF by averaging the NEF values of all edges connected to a node. **c**, **d** Averaged NEF based on **c** the interactions and **d** outgoing approaches. OXTR^ΔAON^ mice showed significantly stronger temporal fluctuations in both interactions and outgoing approaches than non-sRC-member control mice (two-sided permutation test on the ranks, 10,000 iterations). Each dot represents one animal in one round. In the boxplots, the thick central line shows the median. The lower and upper bounds of the box indicate the 25th and 75th percentiles (Q1 and Q3). The whiskers extend from the box to the farthest data point lying within 1.5× the inter-quartile range (IQR) from the box. Shaded areas are a guide to the eye showing the distribution of points within the respective boxplots. **e**, **f** Tuning curves showing how the differences in NEF for **e** the outgoing approaches and **f** incoming approaches of OXTR^ΔAON^ mice compared to controls change as a function of the relative directionality threshold. The black curves show the *p* value of the differences, while the gray curves show the average of the distribution of the NEF for OXTR^ΔAON^ mice minus the average of the distribution of control mice (${\mu }_{{{\rm{OXTR}}}\Delta {{\rm{AON}}}}-\,{\mu }_{{controls}}$). These show that the effect size is maximal at threshold values around 3, as well as the *p*-value of the comparison between outgoing approaches of OXTR^ΔAON^ and control mice. **g** Graphical summary: OXTR^ΔAON^ mice exhibit elevated fluctuations in their social approach patterns, and also their peers interact with them differently. Source data are provided as a Source Data file.

To capture fluctuations of the outgoing approaches at the level of specific peers, a measure is required that could handle weighted, fully connected networks rather than reducing them to binary links. Drawing on concepts from the field of temporal graph theory, we developed the mean normalized edge fluctuation (NEF; Fig. 9a, see Methods for details). In simple terms, NEF quantifies how steadily a mouse maintains its connections to others: low NEF indicates consistent ties, while high NEF reflects erratic, highly fluctuating interactions. For a given mouse, the NEF is computed by quantifying the temporal fluctuation of each of its connections, normalizing these fluctuations by the typical connection strength over the course of the experiment, and averaging the normalized values across partners (Fig. 9b).

Using this measure, we found that male OXTR^ΔAON^ mice both interacted (Fig. 9c) and approached others less consistently (Fig. 9d) than control non-members, as reflected by significantly higher NEF values. Thus, although their overall motivation to initiate approaches was preserved, their targeting of specific partners was unstable, leading to locally fluctuating approach patterns over time. Interestingly, these erratic interaction patterns translated to a diminished consistency of the time spent interacting over the duration of the experiment (Supplementary Figs. 16 and 17). We next asked whether this instability was also reflected in how OXTR^ΔAON^ mice were approached by others in the group. Indeed, incoming NEF values were higher in OXTR^ΔAON^ mice than in control non-members (Fig. 9f, Supplementary Figs. 16–18), indicating that peers engaged with them less consistently over time. Importantly, the difference in both in- and outgoing approaches for OXTR^ΔAON^ mice and non-sRC-member control mice was stable across different relative directionality thresholds (Fig. 9e, f). Finally, we controlled the robustness of these results by assessing their significance and effect sizes over a range of different relative directionality thresholds (see Fig. 9e, f and Methods), which confirmed the validity of our interpretation regarding approaching behaviors. Together, these findings show that OXTR^ΔAON^ mice not only initiate relationships less consistently, but also evoke more variable responses from peers (Fig. 9g).

## Discussion

### Summary

This study reveals that male mice living in larger social groups form stable and reciprocally connected cliques, so-called ‘sRCs’. These tightly connected hubs reflect genuine and selective affiliative bonds. They represent emergent properties of networks of interacting individuals that influence each other. Embedded within groups of predominantly neurotypical mice, mice with a targeted deletion of the OXTR in the AON (OXTR^ΔAON^) failed to integrate into these sRCs despite normal levels of social activity in both dyadic and group contexts. This reveals that state modulation of social sensory processing through oxytocin becomes critical when structured higher-order interactions must be built in complex social situations.

### Stable RCs

Densely connected hubs such as RCs represent a fundamental component of social network organization in both humans and animals^8,14,15^. These structures promote resilience, cohesion, and efficient information flow^1,2,4,7,10,12,13,15^. We focused in mice specifically on sRCs, defined as cliques preserved over at least 80% of the lifetime of a group composed of the same individuals. This approach excluded transient structures that may form by chance or fail to stabilize due to shifting affiliative focus. The formation of sRCs was robust to insertion of a minority of OXTR^ΔAON^ mice, as supported by groups of wild-type mice that never interacted with mutants. Also, groups of either older or younger adults both generally formed sRCs.

In humans, rich-club members often hold positions of influence and exert strong effects on network dynamics^8,13,15^. Consistent with this, sRC-member mice tended to occupy higher competitive ranks and displayed broad chasing also beyond their immediate clique, supporting similarly relatively influential roles in the overall group. In these groups, dominance in tube competitions and chasing has nuanced functions^37^. The hierarchy measured with incidental tube competitions indexed a stable internalized social rank carried by individuals across different group compositions. Chasing functions in these groups as an active mechanism of social rank negotiation within the upper tier of the hierarchy^37^. Such a function of chasing emerges from the given context and is modulated by group size, environments that foster territorial behavior, or limited access to resources^35,38–40^.

### sRC features

Social positions can be determined by intra-individual variables that have been internalized from previous social experiences, so that contextual variables such as the specific group composition play a less important role. In the NoSeMaze system, this applies to social rank and the expression of chasing behavior, both of which are predominantly determined by intra-individual variables and are generally relatively robust against a reshuffling of the group composition^37^. In contrast, belonging to the sRC in one round did not significantly increase the probability of being in the sRC of the next group after reshuffling. Likewise, kinship did not significantly increase the likelihood of belonging to the same clique. This suggests that clique formation emerges anew from the specific interactions and participants within each group; a process expected to involve dynamic learning. The difference between internalized social rank and the ability to build novel social relationships also parallels human behavior, where high rank may facilitate but does not guarantee the ability to form stable contacts^8,12–14^.

### Mice carrying OXTR^ΔAON^ and their deficits

Mice carrying OXTR^ΔAON^ provide mechanistic insight into the relation between higher-order social functioning, social processing states and the specific functions of the OXT system. The activation of OXTRs in the AON puts the sensory system into a state of social processing and increases the signal-to-noise ratio in the olfactory bulb^22^, which disseminates information throughout the brain. The consequences of impaired state induction in mice carrying OXTR^ΔAON^ were surprisingly selective in the complex social environment. They kept normal rates of social interactions in the NoSeMaze. Also, the detailed patterns of olfactory social sniffing behavior were normal in OXTR^ΔAON^ mice during microsequences in simple dyadic tests, supporting the idea that motivation to interact or sample others is mediated by other OXT-modulated brain circuits. However, mice carrying OXTR^ΔAON^ were markedly impaired in forming stable connections in complex social environments. One might have expected that early sensory impairments caused by OXTR^ΔAON^ would predominantly influence numerous aspects of social behaviors such as motivation, while higher social functions such as mastering stable connections in social networks are a process primarily regulated by higher brain regions. Instead, our findings support a non-hierarchical view of social brain functions, in which selective deficits in higher-order social functions can arise from alterations in early sensory processing rather than from dysfunction in higher brain regions. Our findings provide experimental evidence that alterations in early sensory processing and recognition mechanisms can propagate to higher-order functions, extending this principle to the navigation of social networks. In doing so, they complement prior work demonstrating that sensory atypicalities constrain the construction and navigation of internal models of the environment, both social and nonsocial^41–43^.

### Fluctuations in the interactions of the mice carrying OXTR^ΔAON^

The fluctuating approach patterns of OXTR^ΔAON^ carriers to other mice in the group result in failure to integrate into stable cliques. While overall social activity was preserved, the NEFs revealed more erratic and inconsistent patterns of affiliative targeting compared to both control sRC-members and non-members. These instabilities likely emerge from deficits in social memory. The oxytocin-induced sensory processing state enables plasticity during interaction, thereby producing reinforced and better differentiated representations of familiar mice in the olfactory cortices^24^. OXTR^ΔAON^ selectively impairs learning in the social domain, but does not affect non-social olfactory discrimination learning in standard assays^22^ and in the NoSeMaze^37^. Importantly, the OXTR deletion occurred only in adult mice, and thus only after mice had already internalized social rank^37^. Consequently, OXTR^ΔAON^ carriers largely maintained their social rank in new groups^37^, but failed to join new sRCs that require de novo learning (this study). The deficit from OXTR^ΔAON^ in joining sRCs supports that the social sensory state setting actions of oxytocin become particularly relevant in such dynamic situations. These findings underscore the continued role of oxytocin signaling in maintaining coherent social targeting and the formation of new social bonds.

Importantly, these temporal fluctuations also had network-level consequences: neurotypical peers also interacted less consistently with OXTR^ΔAON^ mice, leading to reduced bidirectional stability. This reciprocal disorganization highlights how interactional dynamics in social networks can amplify individual-level deficits, consistent with models of emergent properties of social behavior^8,13,15,19,44^.

### Relations to ASD

This phenotype of OXTR^ΔAON^ mice mirrors some of the key social difficulties in high-functioning ASD. ASD represents the extreme end of a continuum in the capacity to form stable social relationships in the general population^3^. Individuals with ASD may engage in social interactions but often struggle to develop deep, reciprocal relationships in society^2,7^. Many still function well in rigid, structured environments, but encounter difficulties as they grow older when confronted with increasingly complex and flexible social contexts, particularly when forming friendships^2,7,45^. These difficulties affect both personal and professional outcomes^2,4,7,45^. Similarly, OXTR^ΔAON^ mice did not show reduced social interest, but were unable to stabilize their affiliative decisions in complex group situations due to their erratic interaction behavior and the resulting perturbed approach from their peers. Our results therefore suggest that the impairment in OXTR^ΔAON^ mice is not only intrinsic but is amplified by the dynamics of social networks, where inconsistent behavior can cause others to withdraw. The experimental design, in which a minority of cognitively impaired individuals was embedded within predominantly neurotypical groups, mimicked the sparse patterns of genetic variation commonly found in natural populations and was essential for revealing these network-level amplification effects.

Taken together, the longitudinal tracking of the collective behavior of individuals in the NoSeMaze enables the identification of mechanisms underlying non-linear affiliative dynamics. The fluctuations of mice carrying OXTR^ΔAON^ in targeting others undermine the formation of stable reciprocal relationships, despite preserved overall social activity. This demonstrates, firstly, how emergent properties from the mutual interactions among subjects in a social environment amplify individual deficits in social cognition. Secondly, these findings highlight the role that early sensory processing can have in the cognition of higher-order social relationships. Finally, it identifies a molecular mechanism underlying the formation of social network organization. These insights thereby provide a framework for the neural basis of social integration and its disruption in psychiatric conditions.

## Methods

### Ethics statement

All procedures were in accordance with the National Institutes of Health Guide for the Care and Use of Laboratory Animals and the EU 2010/63 directive, and approved by the local animal welfare authority (Referat 35, Regierungspräsidium Karlsruhe, Karlsruhe, Germany).

### Animal strains, husbandry and preparation

#### Animals

All experiments were conducted in adult male mice, backcrossed for >10 generations in a C57BL/6 J background (Charles River, Sulzfeld). To probe oxytocin signaling, we applied two manipulations: (1) selective, adult-onset deletion of the OXTR in the AON (OXTR^ΔAON^) via bilateral rAAV_1/2_-CBA-Cre^22^ injection into OXTR_fl/fl_ mice (B6.129(SJL)-*Oxtr*^*tm1.1Wsy*^/J, RRID: IMSR_JAX:008471, Jackson Laboratory), with control littermates receiving rAAV_1/2_-CBA-dTomato^22^; and (2) optogenetic activation of oxytocin-releasing neurons in the PVN of OXT-Cre mice (B6;129S-*Oxt*^*tm1.1(cre)Dolsn*^/J, RRID:IMSR_JAX:024234, Jackson Laboratory) via rAAV_5_-DIO-ChR2(H134R)-mCherry (Addgene #20297-AAV5). Additionally, as a control group to test if similar sRCs form in groups without OXTR^ΔAON^ mice, we analyzed data from male wild-type C57BL/6 J mice (obtained from Charles River, Sulzfeld); for details on this wild-type cohort see Supplementary Table S4.

#### Sex

The study focused on male mice, for whom social network dynamics are most robustly characterized^46^, and for whom the NoSeMaze system was originally optimized. Including both sexes in the same experimental environment leads to mating behavior, which fundamentally alters social dynamics and network structure. Ongoing work is aimed at adapting the NoSeMaze for use with female cohorts, with the goal of extending these findings to female-only or mixed-sex social systems in future research.

#### Virus vectors and stereotactic surgery

Surgical procedures and histological confirmations followed ref. ^24^. In brief, 79 adult male homozygous OXTR_fl/fl_ mice were used for this part of the study. Mice were divided into two experimental cohorts of different ages. Cohort 1 included 41 mice, which started the NoSeMaze experiment at 16 weeks and completed it at 30 weeks of age. Cohort 2 consisted of 38 mice, aged 55 weeks at the beginning and 90 weeks at the end of the NoSeMaze experiments. Of the 79 mice, 26 mice (cohort 1: *n* = 16, cohort 2: *n* = 10) received bilateral virus injections (rAAV_1/2_-CBA-Cre) into the AON pars centralis to achieve conditional OXTR deletion (OXTR^ΔAON^). The remaining 53 animals were injected with a control vector (rAAV_1/2_-CBA-dTomato). Surgery and virus injection were performed at least six weeks before experiments to allow sufficient time for OXTR depletion. Each animal was also subcutaneously implanted with an RFID transponder (ID 100 trovan®, Euro I.D.) under short-term anesthesia (1.5% isoflurane) for automated individual tracking.

To induce conditional OXTR deletion in one cohort, six mice received bilateral stereotactic injections of an adeno-associated virus carrying Cre recombinase (*rAAV*_*1/2*_*-CBA-Cre*) into the AON pars centralis, while the remaining six animals received a control virus encoding only dTomato (*rAAV*_*1/2*_*-CBA-dTomato*). In another cohort, a Cre-dependent adeno-associated virus expressing channelrhodopsin-2 (*rAAV*_*5*_*-DIO-hChR2(H134R)-mCherry*) was injected to allow optogenetic activation of OXT neurons in the PVN. All stereotactic surgeries were performed under isoflurane anesthesia (induction at 3–4%, maintenance at 1–2%) with pre- and post-operative analgesia (meloxicam, Metacam, Boehringer Ingelheim). Mice were at least 10 weeks old and maintained on a heating pad for the duration of the procedure. After securing the head in a stereotaxic frame (Kopf Instruments), the skull was leveled and a local anesthetic (lidocaine) was applied to the incision site. Virus was delivered using a pulled-glass micropipette attached to a nanoinjector (MO-10, Narishige). A total volume of 0.5 µl was infused at two sites per hemisphere. Coordinates for PVN injections were: 0.1 or 0.3 mm posterior to bregma, 1.0 mm lateral, 4.8 mm ventral from skull surface, delivered at a 10° angle relative to vertical. The optic fiber (FT-200-EMT with ceramic ferrule CFLC230-10, Thorlabs) for optogenetic activation of the PVN-OXT neurons was implanted according to the following coordinates relative to bregma (in mm): 0.2 posterior, 0.8 lateral, 4.3 ventral, at an angle of 10° to the vertical axis. For OXTR deletion in AON, injection coordinates were: 3.0 mm anterior to bregma, 0.7 and 1.2 mm lateral, and 3.5 mm ventral.

#### Immunohistochemistry

Following completion of the experiments, animals were anesthetized and transcardially perfused for histological verification of viral expression. Brains were fixed in 4% paraformaldehyde for two weeks, and then coronally sectioned (50 µm) and stained with antibodies against OXT (anti-OXT (1:1000, mouse; kindly provided by Harold Gainer)), Cre (anti-Cre (1:1000, rabbit; Novagen, cat. n. 69050-3)), or GFP (anti-GFP (1:1000, chicken, Abcam, cat. n. 13970)) diluted in PBS containing 0.2% Triton X-100 (Merck) overnight at 4 °C. The signals were visualized with ALEXA 488 secondary antibody (goat IgG, 1:1000, ThermoFisher Invitrogen, anti-mouse cat. n. A-11001, anti-rabbit cat. n. A-11008, or anti-chicken A-11039), respectively, incubated for 2 hours at room temperature. Fluorescent signals were visualized using either a confocal microscope or a slide-scanning system, in accordance with standard immunohistochemistry protocols (see ref. ^24^). Images were acquired with an Olympus Axio Imager 2 microscope and expression patterns were mapped onto a standard brain atlas.

### Dyadic social interaction experiment

#### Animals and experimental configuration

Each mouse in the OXTR^ΔAON^ group and its corresponding control group participated in two interaction sessions. In contrast, mice in the optogenetic stimulation cohort completed four sessions: two with optogenetically induced oxytocin release and two interleaved control sessions. During control sessions, optical stimulation was prevented by blocking light transmission through insertion of a small non-transparent plastic disc at the junction (mating sleeve) between the ferrule from the patch cord and the optical fiber implant. To minimize fatigue and allow behavioral recovery, animals were given one week between each interaction session.

During each session, the experimental mouse was placed in a clean standard cage (under 70 lux ambient lighting) and introduced to an unfamiliar, adolescent, same-sex C57BL/6 (postnatal days 35–50). This conspecific was selected to minimize the likelihood of aggression or sexual behavior. The dyad was allowed to interact freely for 5 minutes. All sessions were recorded at 30 frames per second using a Sony FDR-X1000V camera. In sessions involving optogenetic stimulation, light pulses (60 pulses per train, 5 ms duration, 30 Hz) were delivered every 30 seconds throughout the interaction period to induce oxytocin release.

#### Analysis of interaction videos

All video recordings were trimmed to 8092 frames ( ~ 4 minutes and 30 seconds) to standardize video length. Frame alignment and resolution were unified to avoid distortions during analysis. Behavioral annotation was conducted manually using BORIS (Behavioral Observation Research Interactive Software^47^), an open-source tool for labeling video-recorded behavior. A custom ethogram was used to classify behaviors into discrete states: anogenital sampling, nose-to-nose sampling, flank sampling, approach, pursuit, attack, climbing, digging, and self-grooming (cf. Fig. 1a, b). All video labeling was blinded to the condition of the mouse (Cre- or dTomato- expression, or optogenetic or sham stimulation). Behaviors were manually labeled using keyboard shortcuts, and outputs were exported in CSV format for further analysis. Inactivity or behaviors unrelated to social interaction like climbing, digging, and self-grooming were post-hoc classified as ‘idle’ states. Pursuit was grouped with approach. No attacks were observed in any session. Subsequent analyses focused on five behavioral states: anogenital sampling, nose-to-nose sampling, flank sampling, approach, and idle. The three sampling behaviors were further grouped as ‘interaction states’ for the state-based analysis, but were kept separate in the transition analysis. Mean duration and frequency of interaction states, approach, and idle were compared using linear mixed-effect models (LME, MATLAB fitlme), with animal ID as a random effect to account for repeated measures. For the optogenetics cohort, the model was: metric ~ intervention + (1|animalID), where intervention = sham vs. oxytocin release. For the OXTR^ΔAON^ cohort, the model was: metric ~ group + (1|animalID), where group = control vs. OXTR^ΔAON^.

To assess sequential behavioral patterns, transition matrices were computed for each animal based on the annotated behavioral state sequences. A transition was defined as a change from one behavioral state (e.g., approach) to another (e.g., nose-to-nose sampling) across consecutive time points. Each matrix represented the relative probability of transitioning from a given source state to a target state, normalized over all outgoing transitions. Matrices were calculated at the individual level and then averaged across animals within each experimental condition (control, OXTR^ΔAON^, sham, oxytocin release). Then, for each transition between behavioral states, LMEs were fit to compare either control vs. OXTR^ΔAON^ or sham vs. oxytocin release, using group (or session type) as a fixed effect and animal ID as a random effect with random slopes. Together with the average matrices for the four experimental conditions, the resulting t-statistics from the LMEs were visualized in matrix form to identify significant differences in transition probabilities between conditions. False discovery rate (FDR) correction was applied to account for multiple comparisons.

### NoSeMaze system

The NoSeMaze is a semi-naturalistic environment for continuous, long-term monitoring of social behavior and reinforcement learning in freely interacting mouse groups without human interference^37^. The full documentation is available at https://github.com/KelschLAB/NoSeMaze. Briefly, each NoSeMaze consists of two connected zones: a housing arena, containing nesting material and free access to food, and an open-field arena where social interactions take place (cf. Fig. 3a). The arenas are linked by two tubes, each equipped with RFID readers at each end that allow for automated tracking of individual movements and interactions. Water is delivered exclusively via a stimulus-outcome learning module connected to the open-field arena, where animals initiate trials ad libitum to meet their water needs. The olfactory learning module was developed based on the Autonomouse habitat^48^. The NoSeMaze design ensures that animals regularly move between the arenas to access both food and water. The open-field arena is continuously recorded using ceiling-mounted infrared cameras, enabling 24/7 video monitoring of spontaneous group behavior throughout each experimental round. All animals learned to use the water lickport, remained healthy, and none were excluded due to injury or social incompatibility over the course of the experiment. Thus, the NoSeMaze provides a relatively peaceful environment with no overt aggression, supporting stable group coexistence.

Two to four parallel NoSeMaze units were used, each housing 9–10 mice for 3-week rounds. Each group included 2–5 OXTR^ΔAON^ mice (see Supplementary Table S1) interspersed among neurotypical controls, mimicking sparse genetic variation. Groups were reshuffled before NoSeMaze entry and again between rounds (see Fig. 3c, d), with most mice participating in multiple rounds. Two separate cohorts, younger (16–30 weeks, *n* = 41, *n* = 16 OXTR^ΔAON^) and older animals (55–97 weeks, *n* = 38, *n* = 10 OXTR^ΔAON^), were tested. Before and after surgery and between the rounds in the NoSeMaze, animals were group-housed (3–5 mice per cage) under controlled environmental conditions (12:12 h light/dark cycle, 24 °C, 55% humidity) with unrestricted access to food and water. Familiarity with the other animals of each cohort was maximized by reorganizing group compositions every 3–4 days, ensuring each mouse interacted with all other cohort members prior to entering the NoSeMaze for the first time.

#### Experimental design and animal grouping

OXTR deletion was performed at least six weeks before the start of the experiments. Over the course of the study, animals were tested across a total of 21 NoSeMaze rounds, each lasting three weeks (see Supplementary Table S2). The majority of mice (*n* = 75) participated in two or more rounds. During each NoSeMaze round, mice were housed in dynamically reconfigured social groups of 9–10 individuals for three weeks, with no human interference during the period of the 3 weeks round. To examine behavioral stability across changing social contexts, group compositions were reshuffled from one round to the next (cf. Fig. 3c). Mice returned to home cages in groups of four between NoSeMaze rounds, with regular regrouping to promote familiarity among potential future group members. The average interval between the start of two successive NoSeMaze rounds was 6.8 weeks (SD = 2.8 weeks). Prior to entering the NoSeMaze, all mice underwent two habituation sessions in the NoSeMaze, conducted in subgroups of 6–7 animals. Each habituation session lasted 16–20 hours and included one full dark cycle, allowing mice to acclimate to the environment and learn the basic functionality of the water lickport (for details on gradual acclimatization, see ref. ^37^). To enable individual identification in the continuous video recordings, mice were marked using non-toxic fur bleaching before each round under light isoflurane anesthesia. These fur patterns were used to train identification networks in DeepLabCut (DLC) for automated tracking.

#### Reward-seeking and social dominance metrics

Behavioral tracking also included automated assessment of reinforcement learning at the water-lick port and social rank and chasing using RFID-based monitoring in the tubes (cf. Fig. 7d, g, see ref. ^37^, and Supplementary Methods). Social rank was quantified by computing David’s scores from automatically detected ‘tube competitions’ (two mice entering from opposite ends; one forced the other to retreat). Additionally, the proportion of initiated and received chases through the tubes was quantified.

Social rank was inferred from interactions occurring in the two tubes connecting the housing and open-field arenas. These tubes were equipped with RFID readers and represented natural bottlenecks that mice had to pass through to access food or water, creating opportunities for competitive and dominance-related interactions. Tube competitions were identified when two animals entered the same tube from opposite ends, resulting in one mouse pushing the other out. The dominant mouse was detected at both ends of the tube, while the subordinate mouse was detected twice at the same entry point, indicating retreat (cf. Fig. 7d). Chasing events were defined as two mice moving in the same direction through a tube in rapid succession, based on specific timing thresholds (i.e., <1.5 s at entry detector, <2 s total transit time, cf. Fig. 7g). Dominance hierarchies were quantified for tube competitions using David’s scores^49^, a standard network-based metric that accounts for both direct wins and losses as well as the hierarchical position of the opponents^37^. In addition, individual social behavior was characterized by the proportion of initiated and received chases^37^.

### Video recording of social interactions

#### Video recording and processing

During each NoSeMaze round, continuous 24/7 videos were recorded in the open-field arena to monitor spontaneous social interactions. A USB 2.0 camera (Aptina MT9V022, FLIR) equipped with a wide-angle lens (focal width: 1.67 mm) captured the arena from above (cf. Fig. 3a). Red LED light strips illuminated the social observation arena, ensuring adequate video quality during both light and dark phases. Videos were analyzed for all groups where video data was complete and of sufficient quality (see Supplementary Table S2) to train DLC^50^. Separate DLC models based on the ResNet-50 architecture were trained for each group to detect the different fur bleaching patterns in the video frames, enabling individual identification and analysis of interaction behavior (cf. Fig. 4c, d). The training set consisted of manually labeled frames from videos recorded on days 1, 5, and 10. Each 24-hour video was divided into 5-minute segments, from which 30-minute clips were assembled based on maximal pixel variation to enrich for mouse activity. Frames were then extracted using DLC’s k-means algorithm to select the most diverse samples. Mice were then manually labeled by trained experimenters based on their fur patterns, ensuring balanced representation of individuals in the training set. Experimenters were fully blind to whether an animal was neurotypical or OXTR^∆AON^. Models were refined iteratively by adding additional training frames until satisfactory prediction accuracy was reached on previously unseen videos (defined as <5-pixel test error with a likelihood p-cutoff of 0.8, and visually validated performance). After training, the entire video dataset was processed using the round-specific DLC model. The output of x–y coordinates and likelihood values for each detected mouse was post-processed to improve accuracy: Predictions with a likelihood below 0.8 were discarded to reduce false positives. Additionally, a temporal filter was applied: mice had to be consistently detected in at least 66% of frames over continuous intervals of at least one second. For two animals in group 3, the models failed to reach sufficient prediction accuracy; these individuals were excluded from further video-based analyses. For group 9, the models failed to reach sufficient prediction accuracy in four out of nine mice, so the entire group was excluded. Moreover, as tracking accuracy declined in the third week of each round due to fading of the fur patterns, data from the 3rd week were excluded from all analyses.

#### Video analysis

First, the time each animal spent in the arena was quantified. Next, social interaction events were extracted based on spatial proximity: an interaction was defined as any instance where the distance between two mice was less than 10 cm for at least 1 second. Successive events separated by fewer than five frames were merged into a single interaction event. For each social interaction, we additionally assessed whether one mouse actively approached the other. To determine this, the smoothed trajectories of both animals were analyzed during the 4 seconds preceding the interaction. If the distance traveled by one mouse exceeded that of the other by a factor of 3 or more, it was classified as the approaching individual. If neither met this threshold—such as when both mice were moving in parallel or converging passively—no approach was assigned. This criterion was chosen to ensure a clear directionality in the approaches, while maintaining a sufficient number of events for the analysis. Since the precise value of this threshold could impact the results of our analysis, we controlled for the robustness of the main results of the article over a range of threshold values from 2 to 4.5 (Supplementary Figs. 3 and 4).

### Social network analysis

#### Network construction and basic metrics

By tracking all events occurring between each pair of animals on a daily basis, we obtained social networks of dyadic (pairwise) interactions. These networks were represented as square matrices, with off-diagonal entries indicating the number of events for each animal pair. Social interaction networks were undirected; accordingly, the matrices were symmetric, with the number of interactions from A to B equal to those from B to A. In contrast, approach networks were directed, capturing how animals initiated or received approaches, resulting in asymmetric matrices. Since each social interaction event had a duration (measured by the number of frames the animals remained within a 10 cm radius), we also constructed undirected networks of mean interaction time, representing the average duration each pair of mice interacted per day.

We first analyzed networks of interactions, approaches, and interaction time aggregated over the first two weeks of each round. To assess group differences between OXTR^ΔAON^ and control animals, we calculated: (1) the total number of approaches initiated (outgoing) or received (incoming) by each animal, (2) the total number of social interactions involving each animal, and (3) the average duration of interactions. These values were computed by summing or averaging the corresponding rows or columns of the aggregated matrices, depending on the respective metric and the symmetry of the matrix. Statistical differences between OXTR^ΔAON^ and control animals were assessed with permutation tests on the median (10,000 permutations).

#### Graph pruning

Beyond analyzing aggregated measures such as the total number of interactions, we aimed to investigate the presence of specific substructures within the social networks and how individual animals contributed to them. To this end, we represented the networks as graphs, where nodes corresponded to individual animals and edges reflected the number of events involving each animal pair. However, due to the high volume of daily interactions, these graphs were densely connected, potentially obscuring meaningful structure. Graph pruning was therefore necessary. Since activity levels varied substantially across groups, standard pruning methods using fixed absolute or relative edge thresholds were impractical. Instead, we applied a mutual nearest-neighbor approach. Specifically, we retained an edge between two animals (A and B) only if they were among each other’s top *k* interaction partners. That is, B had to be among A’s *k* most frequent interaction partners, and A among B’s *k* most frequent interaction partners. We tested several *k* values (see Supplementary Fig. 8), and all results shown in this article were obtained using *k* = 3. The procedure is illustrated in Fig. 5a.

#### Rich-clubs

Having obtained sparser and more interpretable graphs, we next examined whether certain animals formed tightly interconnected subgroups. Specifically, we investigated the presence of RCs, defined as groups of highly connected individuals (i.e., nodes with high degree) that are also densely connected to one another. Formally, a rich-club of degree *k* refers to the subset of nodes *v* with degree *deg*(*v*) ≥ *k* that are more interconnected than expected by chance. To assess this in weighted networks, we computed the weighted rich-club coefficient following the framework proposed by ref. ^51^. In this approach, the observed weighted rich-club coefficient ϕ(k) is compared to that obtained from randomized control networks, ${\phi }_{{rand}}\left(k\right)$. Thus, the *normalized* weighted rich-club coefficient is defined as:

1 $${\phi }_{{norm}}\left(k\right)=\frac{\phi \left(k\right)}{{\phi }_{{rand}}\left(k\right)}=\frac{\frac{C\left(k\right)}{F\left(k\right)}}{\frac{{C}_{{rand}}\left(k\right)}{{F}_{{rand}}\left(k\right)}}$$

where:

- C(k) is the weighted connectedness of the club, defined as the sum of the weights of the edges between the rich-club nodes in the empirical network^51^.
- F(k) is the maximal possible weighted connectedness of the rich-club, defined as the sum of the weights of all edges connected to the rich nodes under the given assumptions.
- ${C}_{{rand}}(k)$ and ${F}_{{rand}}(k)$ are the corresponding quantities averaged across 1000 randomized control networks.

To create randomized control networks, we first rewired the pruned graphs while preserving their degree distribution, and then randomly shuffled the edge weights among the new edges (*n* = 1000). Note that the normalization ensures that ${\phi }_{{\mathrm{norm}}}\left(k\right) > 1$ indicates a true weighted rich-club structure, i.e., that highly connected nodes allocate more weight to their mutual connections than expected by chance.

We were mainly interested in whether RCs remained stable over time. Thus, we assessed their existence by aggregating the social networks over consecutive three-day periods: a 1-day resolution made visualization highly impractical, while a 7-day resolution was too coarse to capture meaningful variations. Although some members of the rich-club varied across time windows, other individuals were consistently part of it. We defined an animal as part of the ‘stable rich-club’ (sRC) if it was included in the rich-club in at least 80% of the networks (i.e., in four out of five graphs). For practical reasons, this analysis was conducted on social interaction networks; however, similar sRCs ( ~ 80% overlap) were observed in networks based on approaches and interaction durations (see Supplementary Fig. 9).

#### Null model testing

To assess the significance of our findings on RCs and their temporal stability, we employed null model testing, comparing the experimentally observed values to distributions generated under random conditions to obtain the statistical significance of our results. Each null model represents the expected outcome under the assumption of no systematic relationship between group membership (e.g., OXTR^ΔAON^ vs. controls, or littermates vs. no littermates) and rich-club inclusion. The specific implementation of the null model depended on the hypothesis being tested (e.g., mutant exclusion, influence of family ties), but all were based on 10,000 random permutations of relevant experimental labels (e.g., IDs, litter indices, round number).

##### Exclusion of OXTR^ΔAON^ mice from the sRC

Specifically, to test whether OXTR^ΔAON^ mice were excluded from the sRC more often than expected by chance, we randomly reassigned IDs within each group 10,000 times. For each iteration, we counted how many randomized IDs of OXTR^ΔAON^ mice occupied positions corresponding to sRC members identified experimentally. This null model assumes that all mice within a group have an equal probability of being part of the sRC. Significance was assessed by counting the number of iterations in which the number of randomized OXTR^ΔAON^ indices in the sRC was less than or equal to the empirical value (one-sided test).

##### Influence of family ties

To examine whether sRC members were more likely to come from the same litter, we randomized litter indices within each group 10,000 times. For each iteration, we counted the number of groups in which at least two sRC members obtained the same litter index. Statistical significance was assessed by counting how many iterations produced a number of randomized groups with littermates in the sRC that was less than or equal to the number observed in the actual experiment.

##### Stability of sRC membership across group reassignments

To assess whether sRC membership reflects a stable individual trait or a characteristic of group-level dynamics, we tested whether mice that had previously been part of the sRC were more likely to re-enter it after being reassigned to a different NoSeMaze group. Notably, the group reshuffling procedure ensured that animals were housed with different group members in each NoSeMaze round. We first identified all mice that (1) participated in at least two NoSeMaze rounds, and (2) had been part of the sRC at least once. For each of these mice, we looked at every later round in which they were placed into a new NoSeMaze group. After randomly reassigning IDs within that group, we checked whether the mouse again became part of the sRC. To avoid potential bias in defining the ‘initial’ sRC membership, one of the eligible rounds was randomly selected as the reference round for each mouse. This null model was repeated across 10,000 independent iterations, each time recording how many of these previously sRC-assigned mice re-entered the sRC at least once following reshuffling. The resulting distribution served as a null model of sRC re-entry, representing the level of membership conservation expected by chance under the assumption that group membership is unrelated to individual traits.

### Graph-theoretical metrics

To investigate how sRC members differ from OXTR^ΔAON^ and control animals that were not part of the sRC, we computed two graph-theoretical metrics: out-strength and mean NEF, which were calculated separately for incoming and outgoing directionality using the Python implementation of the ‘igraph’ toolbox^52^ or custom-implemented scripts with Python’s NumPy package (see below). The corresponding code is available at https://github.com/KelschLAB/NoSeMaze-stableRichClubs.git. In brief, out-strength captures the total number of approaches initiated by an individual, while the NEF provides a measure of how strongly an individual’s social ties fluctuate over time. Detailed descriptions and formulas for each metric are provided below. All metrics were computed on a daily basis for each individual without aggregating the social networks, as their temporal dynamics provide meaningful insights. To better disentangle the behavior of OXTR^ΔAON^ animals from the reactions of their social interaction partners, analyses were based on directed graphs constructed from approach networks. Group differences in the graph metrics (sRC members, non-sRC controls, and OXTR^ΔAON^) were assessed using permutation tests (*n* = 10,000) based on group medians, applying Phipson and Smith’s correction for extremely low values^53^. To account for repeated measures, group labels were shuffled at the level of the animal, ensuring that all observations from the same individual remained assigned to the same group.

#### Out-strength

For node i, ${s}_{i}^{{out}}$ quantifies the cumulative strength of its outgoing connections, i.e, the total weight of edges from node i to its neighbors:

2 $${s}_{i}^{{out}}=\,{\sum}_{j\in {N}_{i}^{{out}}}{w}_{{ij}}$$

where ${N}_{i}^{{out}}$ is the set of nodes that receive edges from i, and ${w}_{{ij}}$ is the weight of the edges from ${i}$ to j. We calculated both the mean out-strength across time and the standard deviation of its temporal derivative, with the latter reflecting the variability in an individual’s motivation to initiate interactions.

#### Mean NEF

In contrast to the standard deviation of the derivative of strength, which captures fluctuations in an individual’s overall activity level, NEF assesses variability at the level of individual connections, thereby providing a more fine-grained measure of temporal instability in social ties. For node ${i}$ in a network ofN nodes, NEF is defined as:

3 $${{NEF}}_{i}=\frac{1}{N}{\sum}_{j\ne i}\frac{{{\rm{fluctuations}}}\left[{e}_{{ij}}\left({{\rm{t}}}\right)\right]}{{{\rm{baseline}}}\left[{e}_{{ij}}\left({{\rm{t}}}\right)\right]}=\frac{1}{N}{\sum}_{j\ne i}\frac{{{\rm{MDM}}}\left[{e}_{{ij}}\left({{\rm{t}}}\right)\right]}{{{\rm{Med}}}\left[{e}_{{ij}}\left({{\rm{t}}}\right)\right]}$$

where ${e}_{{ij}}\left({{\rm{t}}}\right)$ is the time-series obtained by taking the values of the edge between i and j as a function of time, *Med* is the median and MDM is the mean deviation to the median^54^ defined as:

${{MDM}}[e_{{ij}}\left({{\rm{t}}}\right)]=\,\frac{1}{T}\mathop{\sum }_{t=1}^{T}|{e}_{{ij}}\left({{\rm{t}}}\right)-{Med}({e}_{{ij}})|$ In other words, the NEF gives an estimation of the average scale of the fluctuations of the different edges connected to a given node. The normalization of each edge by its typical level of activity is necessary, as the strength of the fluctuations is proportional to its baseline activity. Using the median for the assessment of the fluctuation and baseline level makes the NEF less sensitive to outliers. Note that the choice of method for computing fluctuations in the NEF is not restricted to the MDM, as other robust estimators such as the inter-quantile range (IQR) or Rousseeuw and Croux’s Qn work equally well (see Supplementary Fig. 18)^55^.

In summary, the NEF provides a node-level overview of how variable an individual’s connections are over time, normalized by the typical magnitude of these connections. For directed networks, NEF was computed separately for outgoing edges (from i to others) and incoming edges (from others to i).

To ensure that the statistical conclusions on the approach behavior drawn from the NEF remain stable across a range of relative directionality threshold, we evaluate the NEF across a range of relative directionality threshold values (see Fig. 9e, f). To gain a sense of effect sizes, we display the difference between the mean of the distribution of the NEF’s value of OXTR^ΔAON^ mice and control mice, as a function of the relative directionality threshold parameter (shown as the gray ${\mu }_{{{\rm{OXTR}}}\Delta {{\rm{AON}}}}-{\mu }_{{control}}$ line in Fig. 9e, f).

### Software and statistical analysis

Data processing was performed using custom-written scripts in Matlab (Mathworks), R (https://www.r-project.org/, v4.4.1), and Python (https://www.python.org, v3.13). Information about statistical tests used is given either in the related method sections or the figure legends. LMEs were performed in MATLAB. Social network analyses were conducted in Python using the ‘igraph’^52^ and ‘networkX’^56^ toolboxes, and the statistical analysis of the results was conducted using the Numpy, Pandas, and Scipy packages. Note that all permutation tests accounted for the repeated presence of animals across multiple rounds by shuffling at the level of the animal, ensuring that all observations from the same individual remained assigned to the same group.

Our primary outcomes are longitudinal network-derived measures (e.g., stable rich-club membership and edge-dynamics) from interdependent observations within reshuffled groups, for which standard closed-form a priori power calculations are not readily applicable. Sample size was therefore set by NoSeMaze capacity (up to four parallel units, 9–10 mice per 3-week round) and the design requirement to test the OXTR^ΔAON^ minority in group hypothesis (2–5 mutants per group).

### Reporting summary

Further information on research design is available in the Nature Portfolio Reporting Summary linked to this article.

## Supplementary information

Supplementary Information

Peer Review file

Reporting Summary

## Source data

Source Data 1

Source Data 2

## Data Availability

The data generated in this study are under active use by the reporting laboratory; all data presented in this manuscript are available upon request from the Lead Contact. Source data are provided with this paper.

## Code availability

The code used for the data analysis^57^ is available online via https://github.com/KelschLAB/NoSeMaze-stableRichClubs
