# Supplementary material for: Stable clique membership in male mouse societies requires oxytocin-enabled social sensory states
Source: Nat Commun. 2026 Aug 17;17:8493. doi: 10.1038/s41467-026-76841-5 (PMC13484484; doi:10.1038/s41467-026-76841-5)
Supplement: Supplementary file 1 [file 41467_2026_76841_MOESM1_ESM.pdf]

Supplementary Figures and Tables

Stable clique membership in male mouse societies requires oxytocin-enabled social sensory states

Corentin Nelias[1,2,#], Sarah Ghanayem[1,2,#], David Wolf[1,2], Marcel Moor[1], Danai Nikolantonaki[1,2], Eda Dilara Turgut[1,2], Max F. Scheller[1], Valery Grinevich[3], Jonathan R. Reinwald[1,2,§], Wolfgang Kelsch[1,2,§,*]

[1] Dept. of Psychiatry and Psychotherapy, University Medical Center Mainz, Johannes-Gutenberg University, Untere Zahlbacher Strasse 8, 55131 Mainz, Germany; [2] Dept. of Psychiatry and Psychotherapy, Central Institute of Mental Health, Medical Faculty Mannheim, Heidelberg University, Square J5, 68159 Mannheim, Germany; [3] Dept. Neuropeptide Research in Psychiatry, Central Institute of Mental Health, Medical Faculty Mannheim, Heidelberg University, Square J5, 68159 Mannheim, Germany; [#] shared first authors, [§] shared last authors, * corresponding author: wokelsch@uni-mainz.de

Supplementary Figures 1 – 19

Supplementary Tables S1 – S5

Supplementary Figures

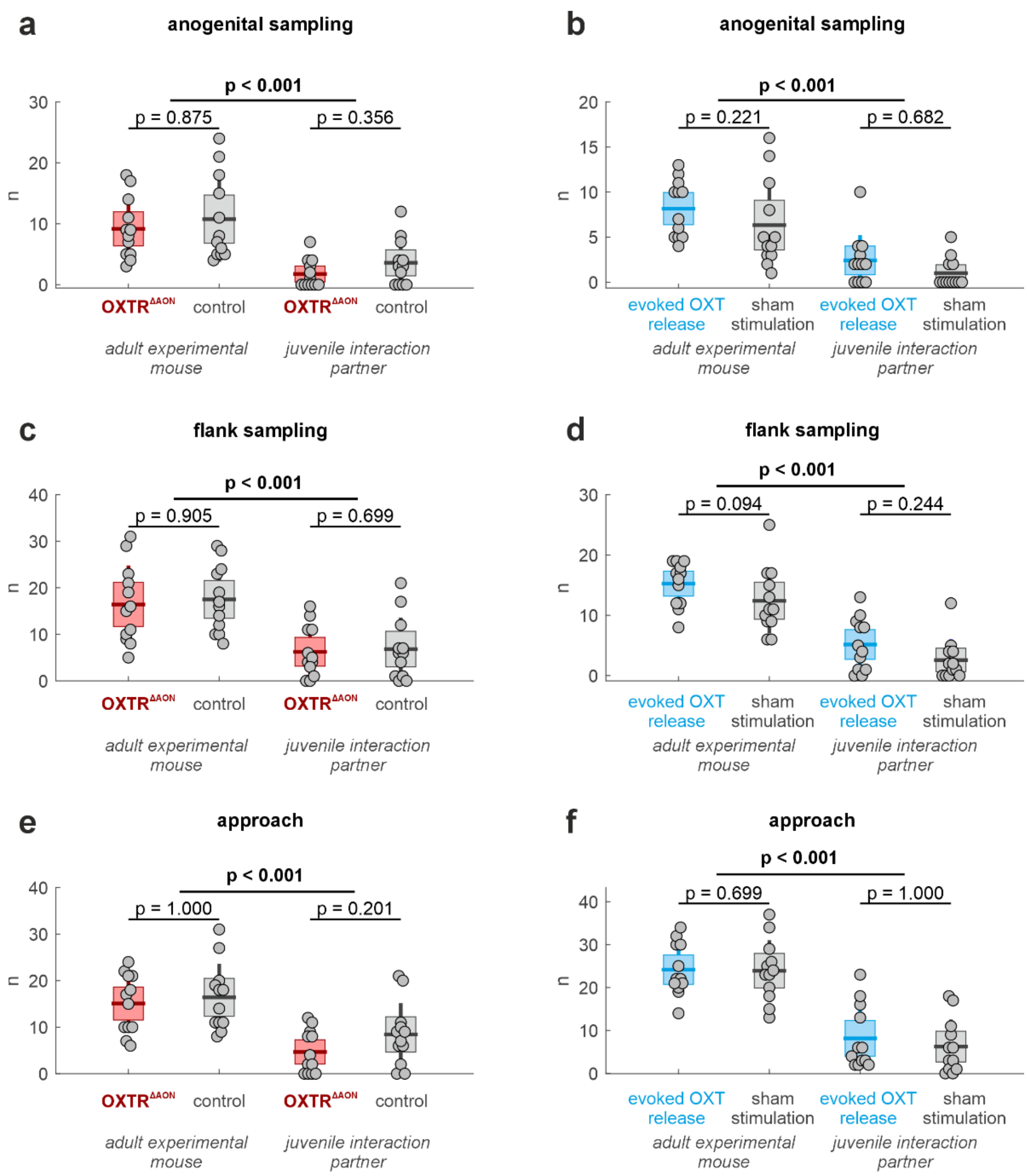

Supplementary Fig. 1: Sampling and approach behaviors in adult experimental mice and their juvenile partners during brief dyadic exploration.

OXTR$^{\Delta AON}$ vs. control sessions (left): Frequency of anogenital sampling (**a**), flank sampling (**c**), and approach behavior (**e**) initiated by adult experimental mice (OXTR$^{\Delta AON}$ vs. control; left panels) and by their respective juvenile interaction partners (when paired with OXTR$^{\Delta AON}$ or control adults; right panels). Behaviors of adult experimental mice and juvenile interaction partners were quantified separately and compared across genotypes of the experimental animal. No significant differences were observed across genotypes in either group (two-sided permutation test on the median, n = 10,000 permutations). However, across all categories, adult mice exhibited higher levels of social sampling than their juvenile partners. *Evoked OXT release vs. sham stimulation sessions (right):* Frequency of anogenital sampling (**b**), flank sampling (**d**), and approach behavior (**f**) in mice undergoing optogenetically evoked oxytocin (OXT) release compared to sham stimulation. Data are shown separately for adult experimental mice (left panels) and their juvenile interaction partners (right panels).

Each dot represents one individual per session.

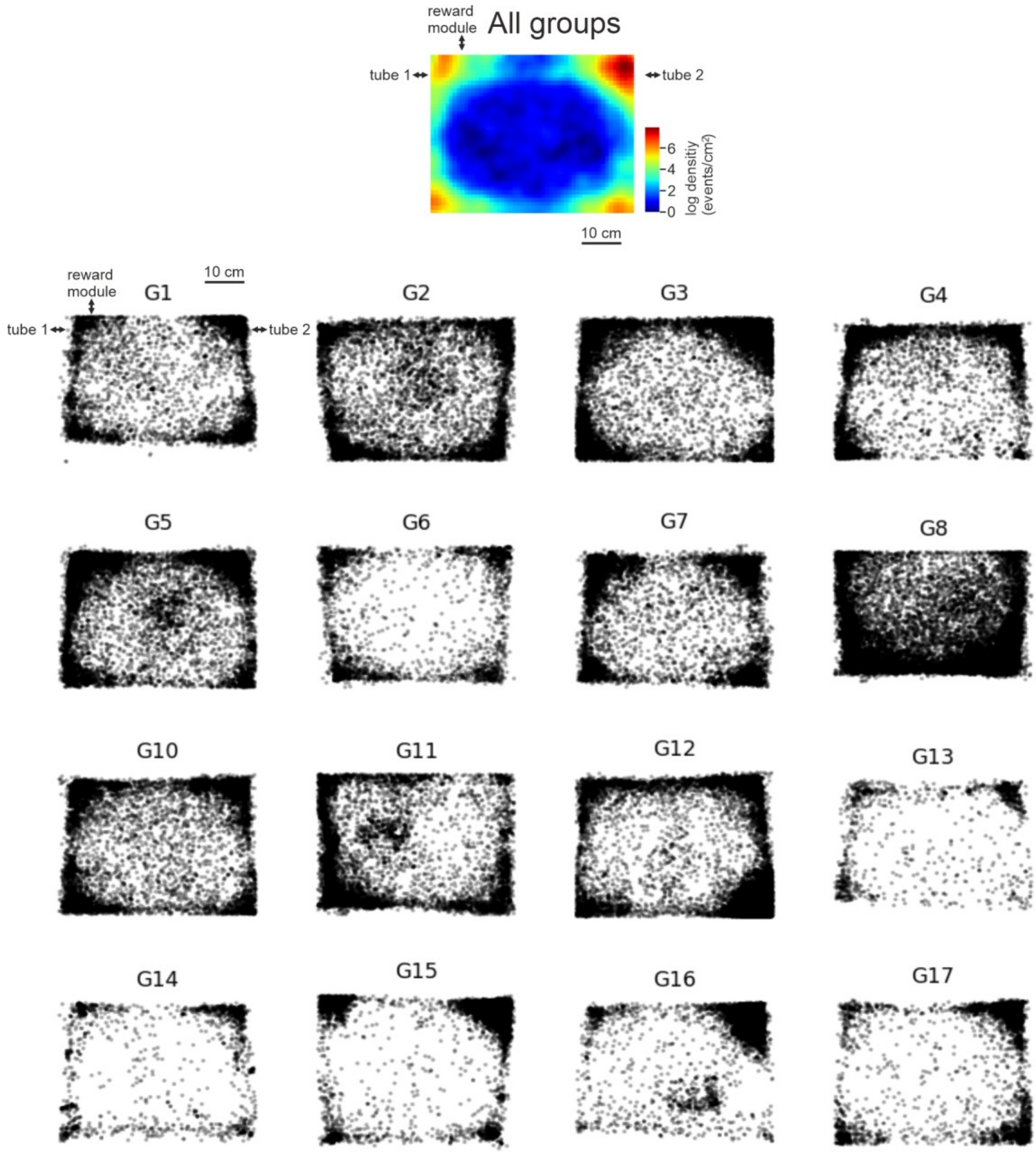

Supplementary Fig. 2: Spatial distribution of interaction events across all 16 NoSeMaze groups.
The top panel indicates the density of recorded interactions as a function of spatial position within the social arena, averaged over all groups from the study. Each numbered panel (G1–G8, G10-G17; G9 excluded, see Methods) shows the spatial locations of individual interaction events detected over one full NoSeMaze round for a given group. Each dot represents a social interaction defined by physical proximity (<10 cm for ≥1 sec) between two individually tracked mice. Interaction activity was most pronounced around the corners and along the arena walls, particularly near access points such as entry tubes and the reward module (top wall).

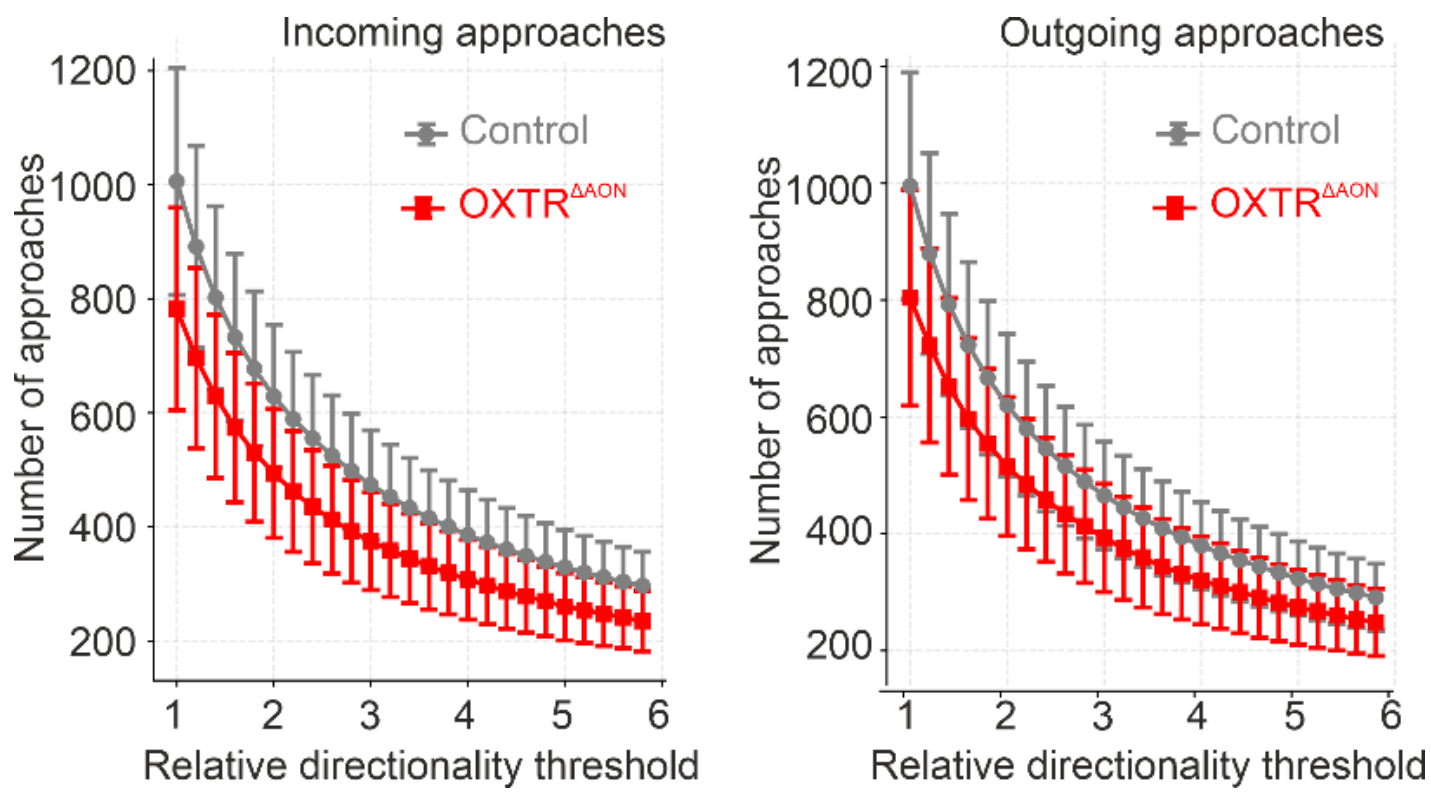

Supplementary Fig. 3: Variation of the number of detected approaches as a function of the relative directionality threshold.
In both directions (outgoing and incoming), the number of detected approaches decreases as the relative directionality threshold increases. This decrease is very sharp at low threshold values, reflecting the fact that the directionality of approaches at threshold values below 2 is most likely driven by a certain randomness in assigning directionality. Therefore, to capture volitional approaches, we set the relative directionality threshold for further analyses to 3. Error bars represent the SEM.

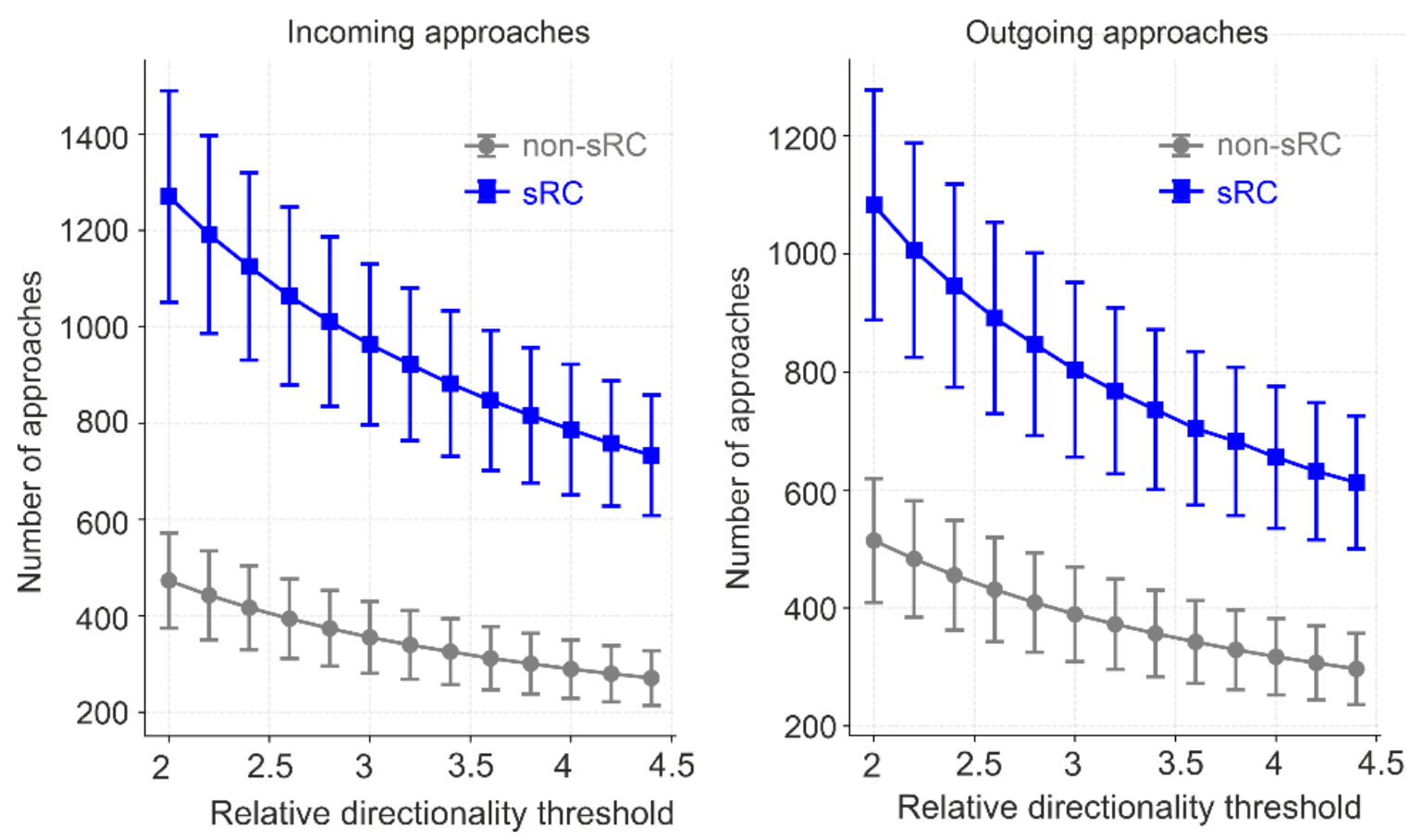

Supplementary Fig. 4: Variation of the number of detected approaches as a function of the approach distance threshold, shown for sRC members and non sRC members.
In both directions (outgoing and incoming), the number of detected approaches decreases as the relative directionality threshold increases and follow the trends that was described in Supplementary Fig. 3. To the groups (sRC in blue and non-sRC in gray) include both controls and $OXTR^{\Delta AON}$ mice. Error bars represent the SEM.

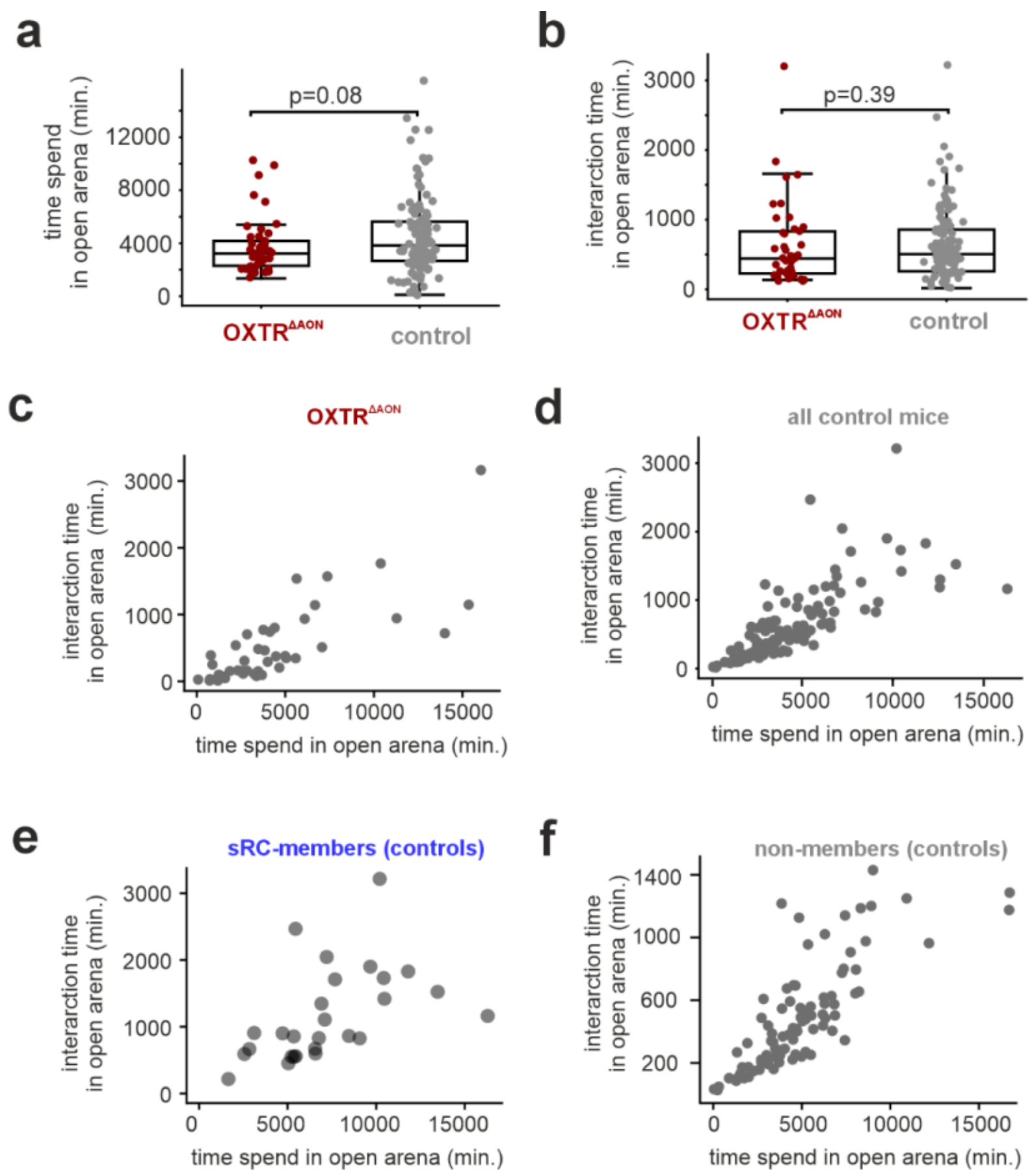

Supplementary Fig. 5: Time spent in the open arena and social interaction time in OXTR$^{\Delta AON}$ and control mice.

a) Total time spent in the open arena per round was slightly reduced in OXTR$^{\Delta AON}$ compared to control mice (p = 0.08, two-sided permutation test on the mean with 10,000 permutations accounting for animal identity). **b)** Total time spent in social interactions in the open arena did not differ significantly between OXTR$^{\Delta AON}$ and control mice (p = 0.86, two-sided permutation test on the mean with 10,000 permutations accounting for animal identity). **c–f)** Correlations between total time spent in the open arena and interaction time was similar across all groups: **c)** OXTR$^{\Delta AON}$ mice; **d)** all control mice; **e)** control sRC-members; **f)** control non-sRC-members. Each dot represents one individual per session.

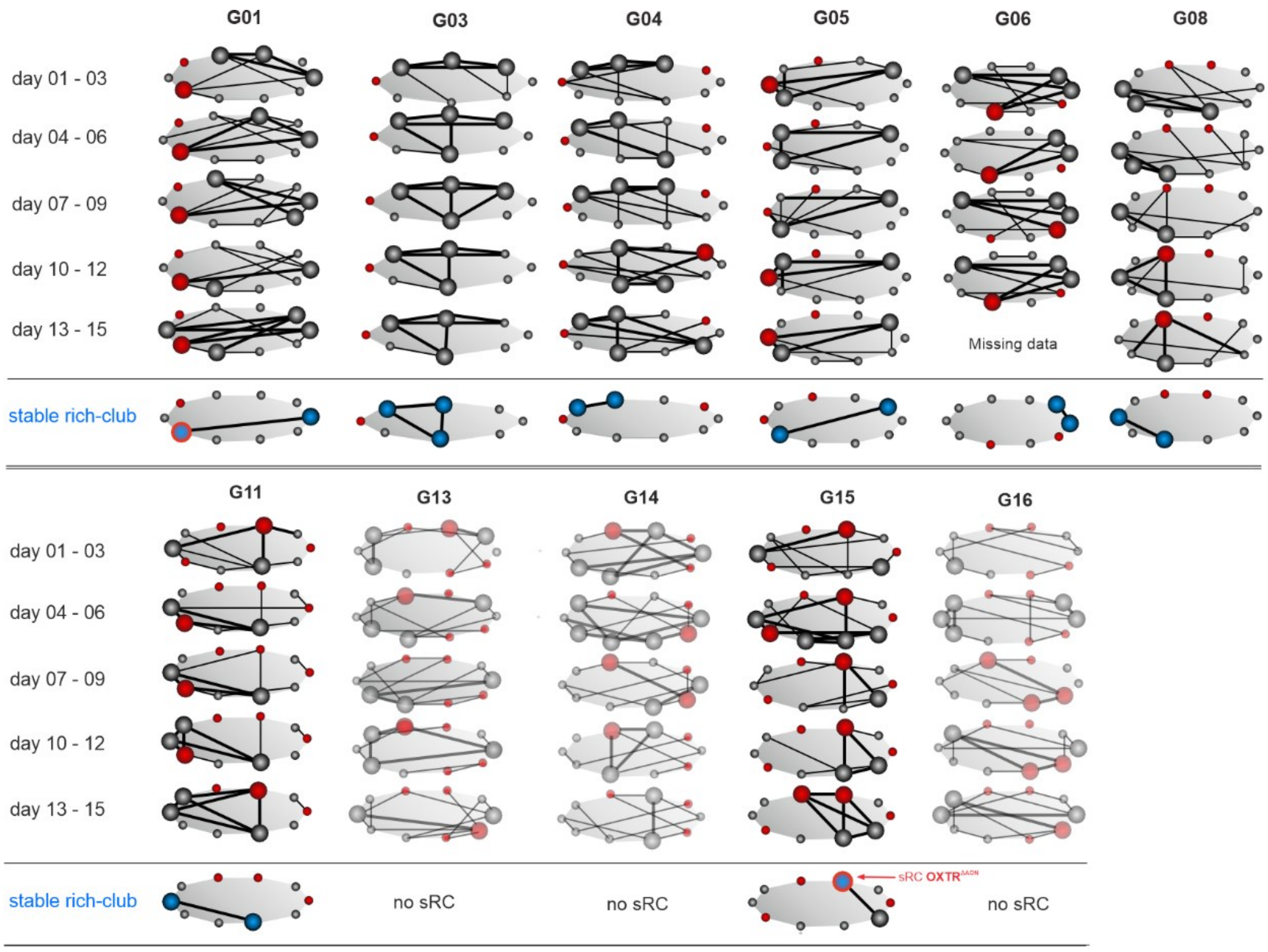

Supplementary Fig. 6: Rich-club dynamics across different NoSeMaze groups.
Rich-club (RC) dynamics are shown for 11 groups (G01, G03–06, G08, G11, G13–G16) across five consecutive 3-day intervals, displayed in horizontal columns. The remaining groups (G02, G07, G10, G12, G17) are displayed in Fig 4c of the main text. Each node represents an individual mouse. Edges reflect the total number of interactions between two mice, with the black edges representing the pruned networks using the mutual nearest-neighbor algorithm (see Methods). Stable RC (sRC) members, defined as nodes maintaining RC membership in ≥80% of time bins, are shown in bold blue in the last row of each column; transient RC members in bold gray. $OXTR^{\Delta AON}$ mice are indicated in red. Groups are divided into older (top) and younger (bottom) sub-cohorts. Groups lacking stable RC structure (G13, G14, G16) are shown with light transparency. G06 has missing data for days 13–15.

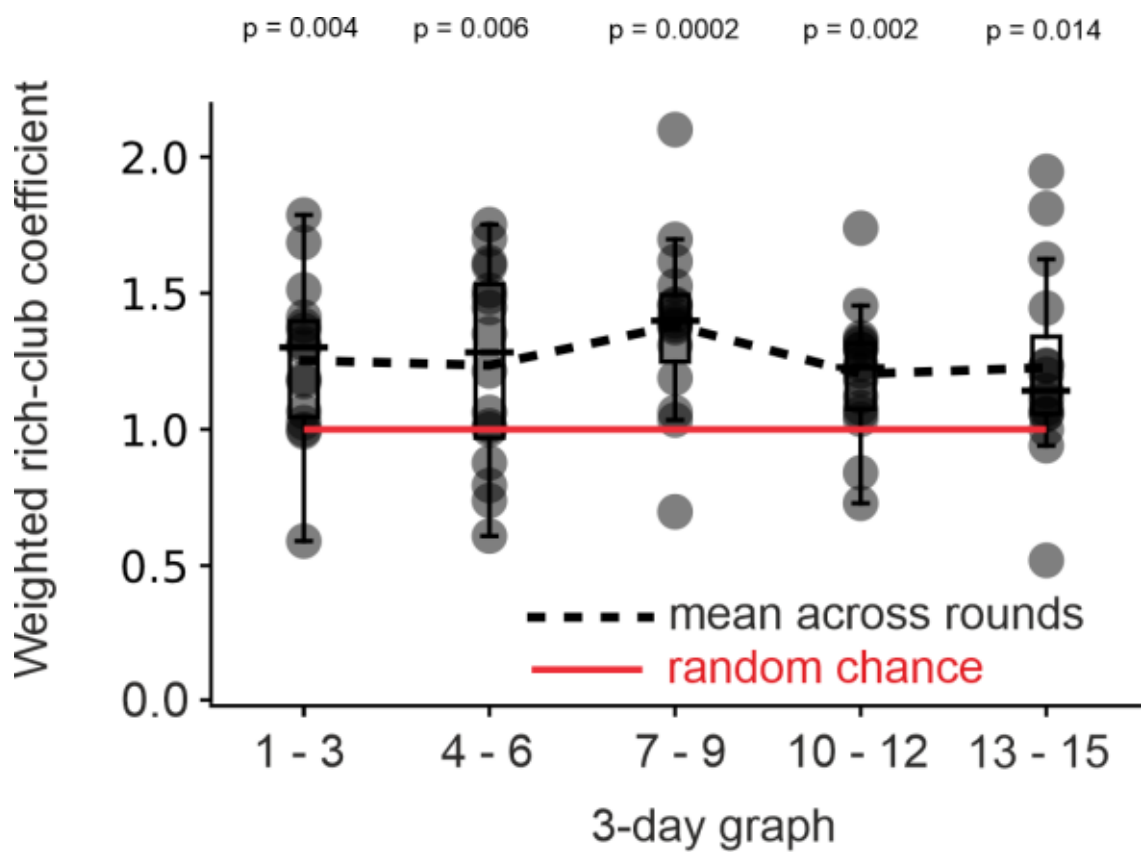

Supplementary Fig. 7: Sustained presence of weighted rich-club organization across time.
The normalized weighted rich-club coefficient ($\phi_{norm}(k)$) is shown across five consecutive 3-day windows spanning the 15-day observation period, for nodes of degree k ≥ 3. Each dot represents the normalized weighted rich-club coefficient for one NoSeMaze group; box plots indicate the distribution (median and interquartile range). The dashed black line shows the average rich-club coefficient across groups; the red line marks the null expectation ($\phi_{norm}(k)$ = 1) derived from randomized control networks. A normalized rich-club coefficient greater than 1 indicates that high-degree nodes (i.e., animals with many social connections) are more strongly interconnected and allocate disproportionately more weight to their mutual interactions than expected by chance (see methods). P-values for each 3-day experimental windows are shown on top of the corresponding box plots (two-sided one sample t-test).

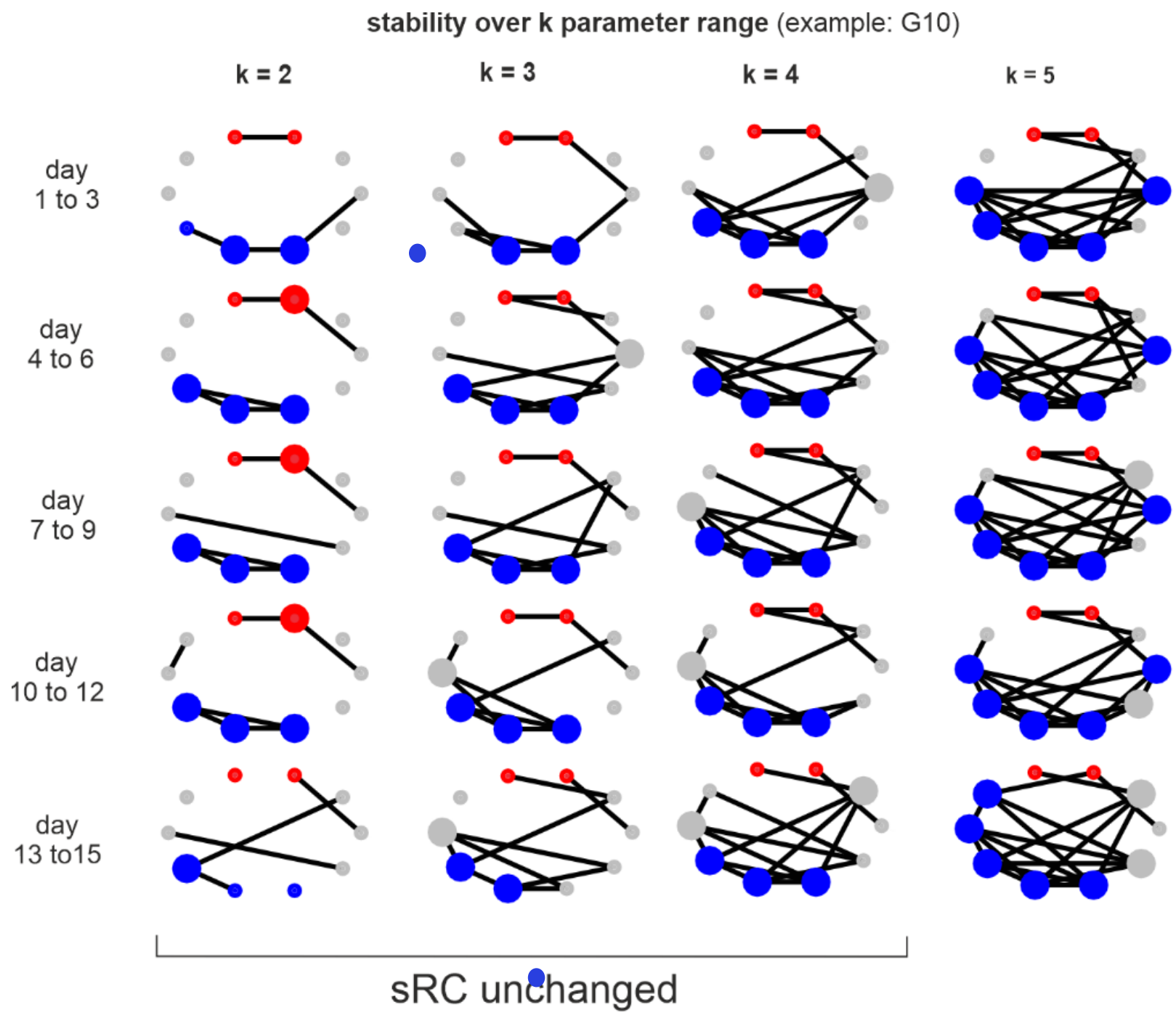

Supplementary Fig. 8: Robustness of stable rich-club detection across different mutual nearest-neighbor pruning levels.

Rich-club (RC) structure for group G10 is shown across five 3-day windows and four mutual nearest-neighbor pruning levels (*k* = 2–5). Each node represents an individual mouse. Edges represent social ties that survive pruning, i.e., pairs of animals that are mutually ranked within each other's top *k* most frequent interaction partners (see Methods). RC members identified at each pruning level are shown as large nodes; non-members as small nodes. Stable RC members (present in ≥80% of time bins) are colored blue. $OXTR^{\Delta AON}$ mice are shown in red. As *k* increases, more edges are retained, but the core stable RC structure remains consistent, demonstrating robustness of RC detection to pruning stringency.

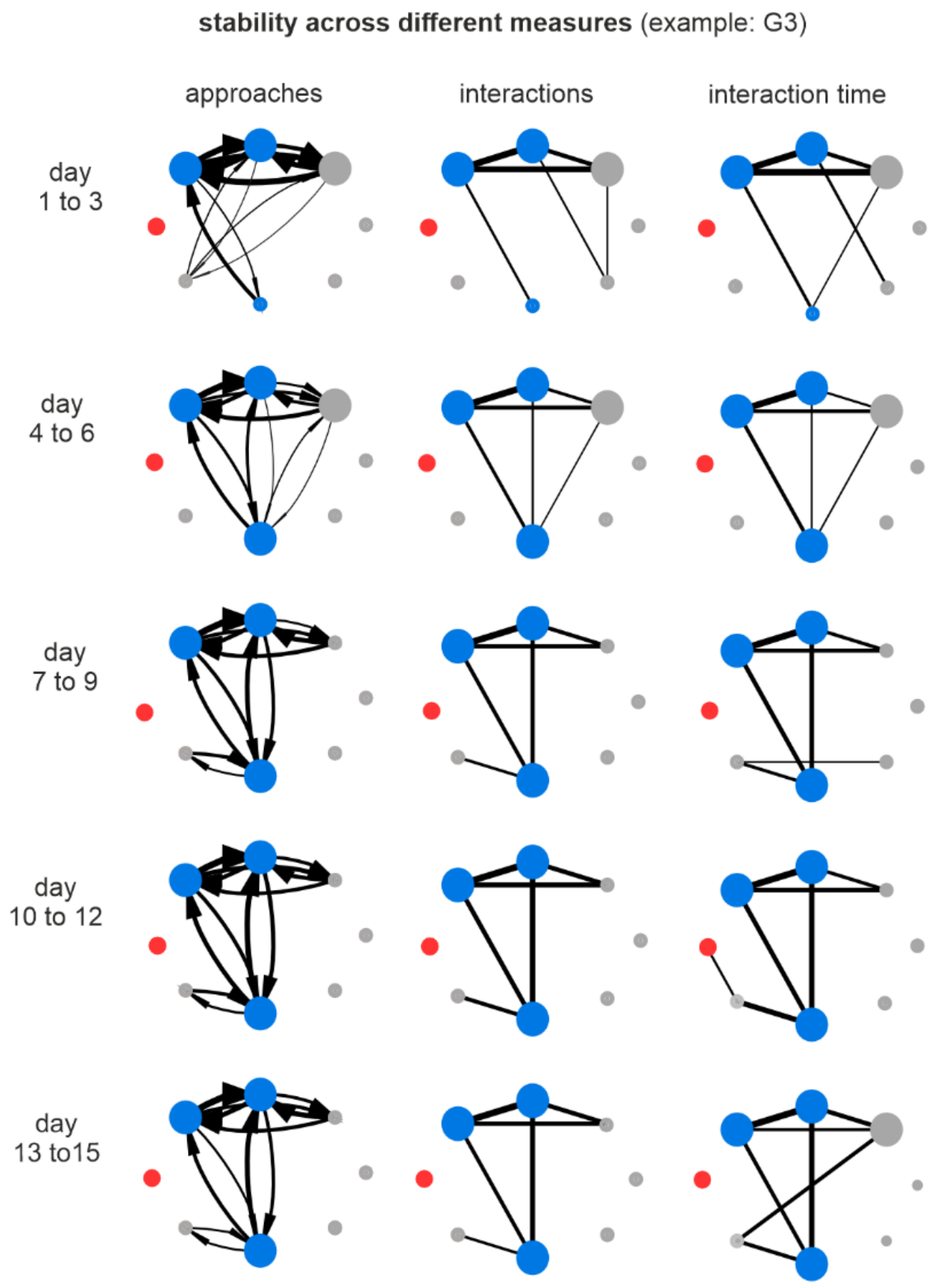

Supplementary Fig. 9: Consistency of stable rich-club detection across different interaction metrics for social network construction.

Rich-club (RC) structure for group G3 is shown across five 3-day windows and three network types based on different behavioral measures: approaches (left), number of interactions (middle), and interaction durations (right). Each node represents an individual mouse. Edges represent social ties that survive mutual nearest-neighbor pruning (k = 3), i.e., pairs of animals that are mutually ranked within each other's top three most frequent interaction partners (see Methods). RC members identified for each measure are shown as large nodes, non-members as small nodes. Stable RC members (present in ≥80% of time bins) are colored blue. $OXTR^{\Delta AON}$ mice are shown in red. Despite variation in the behavioral metric used for social network construction, the core RC structure remains consistent across network types, demonstrating the robustness of RC detection to different definitions of social interaction.

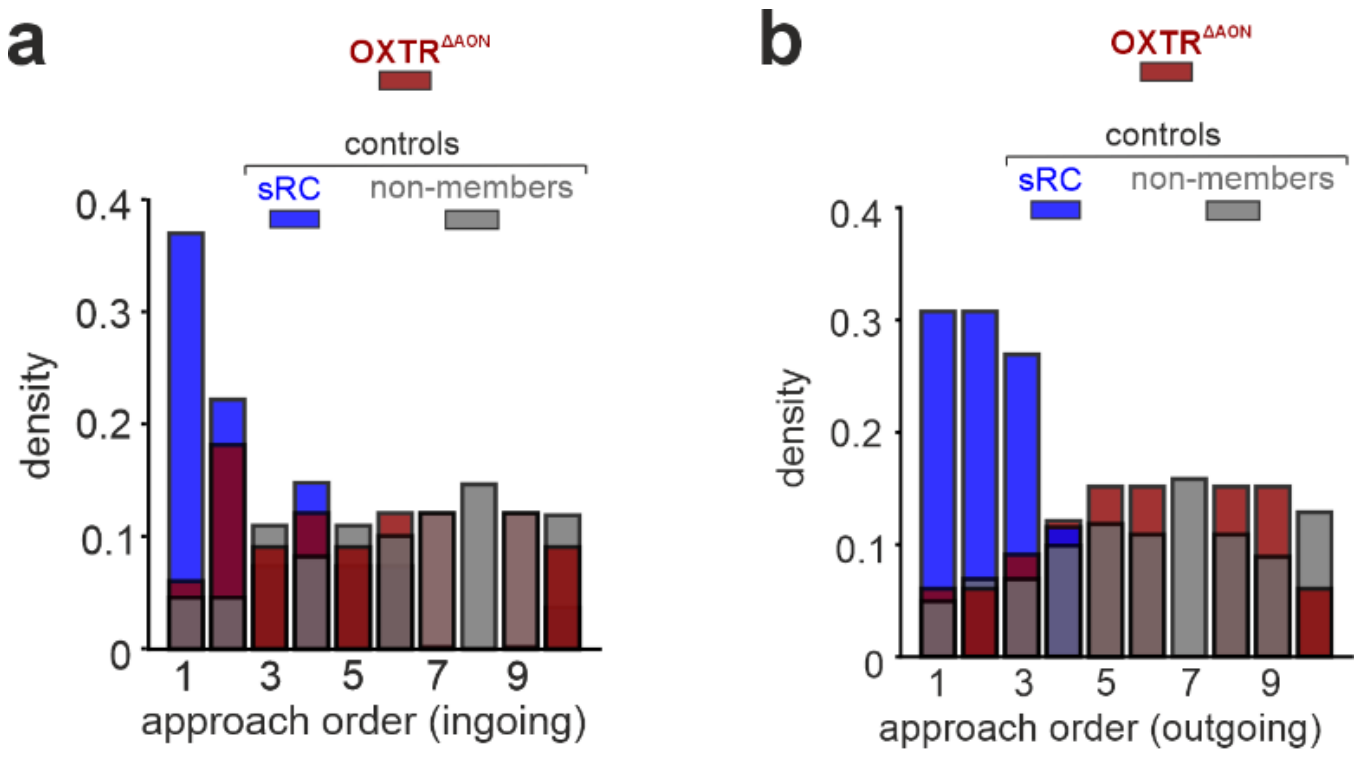

Supplementary Fig. 10: Approach distribution frequency across genotypes and stable rich-club membership.
Distribution of behavioral measures of mice subdivided into three groups: sRC-members (blue), non-members (gray), and OXTRΔAON mice (red). sRC and non-members here only show control mice, so that no animal is represented more than once across groups. Normalized density distributions of within-group behavioral ranks (1 = highest activity) based on **a)** the fraction of total ingoing approaches by mice across the three groups and **b)** the fraction of total outgoing approaches received across the three groups. All densities are normalized (area under each curve = 1) to allow comparison across groups with unequal sample sizes. Significant differences between the distribution of sRC members (blue) and both non-members (gray) and $OXTR^{\Delta AON}$ mice (red) were observed in all cases (two-sided t-test, p = 5.4e-13 and p = 5.9e-11 for ingoing and outgoing approaches respectively), while no differences were detected between non-members and $OXTR^{\Delta AON}$ mice.

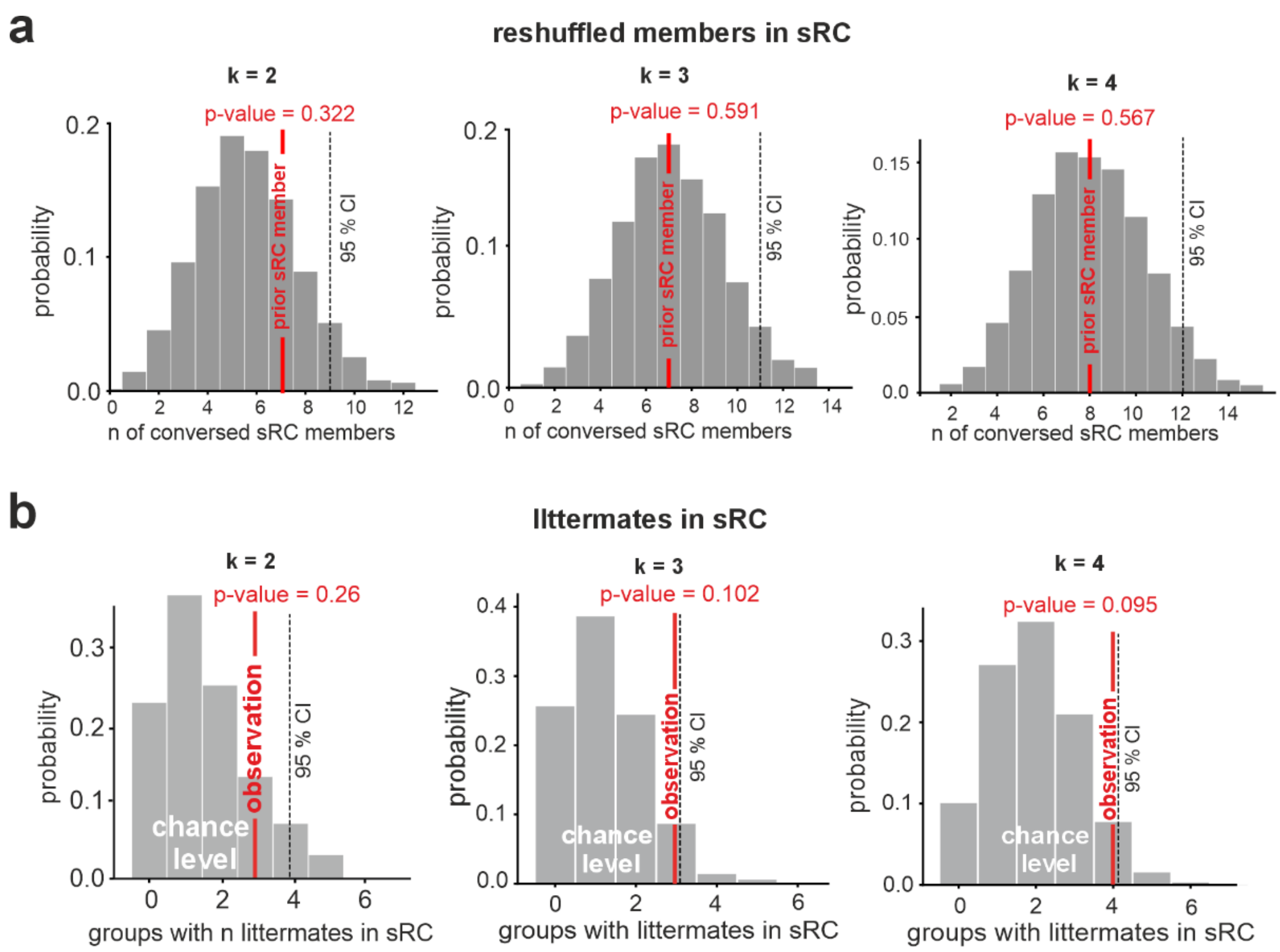

Supplementary Fig. 11: Robustness of stable rich-club composition across different pruning parameters, assessed via permutation tests.

a) Number of conserved sRC members across reshuffled group contexts. The observed values (red lines) represent the number of prior sRC members who rejoined a sRC after random reassignment of group membership. Across all pruning levels (*k* = 2–4), the observed values fall within the null model distribution, suggesting that sRC membership is not a persistent individual trait, but rather emerges from the specific social dynamics of each group.

b) Observed number of groups containing littermates within the same sRC compared to a null distribution generated by randomly shuffling group member identities (10,000 iterations). The observed values (red lines) fall within the 95% confidence intervals of the null model (gray distributions), indicating that shared family background does not significantly influence sRC formation.

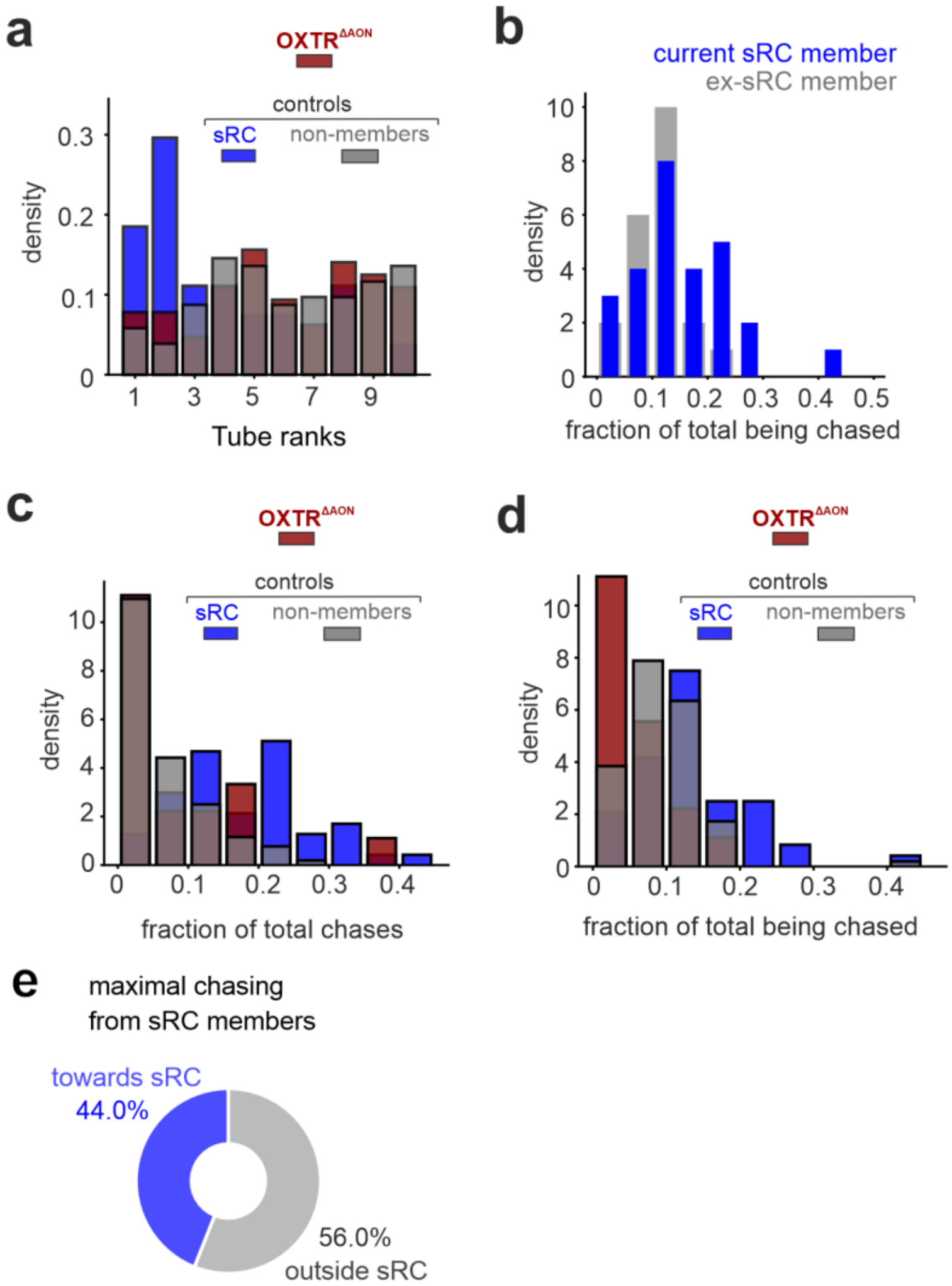

Supplementary Fig. 12: Chasing and social rank across genotypes and stable rich-club membership.
a) Distribution of behavioral measures of mice subdivided into three groups: sRC-members (blue), non-members (gray), and OXTRΔAON mice (red). sRC and non-members here only show control mice, so that no animal is represented more than once across groups. Normalized density distributions of within-group behavioral ranks (1 = highest activity) based on social ranks derived from the integrated tube-test (illustrated in Fig. 6d). The distributions of events of sRC members (blue) were different both from non-members (gray) and OXTR$^{\Delta AON}$ mice (red) (p = 0.007, two-sided permutation test on the median, 10000 iterations). **b)** Fraction of total chases received by current sRC members (blue) and the same individuals when not in the sRC (ex-sRC members, gray). Being part of the sRC did not significantly change the distribution of being chased (p-value = 0.07, two-sided permutation test on the mean, 10000 iterations). **c-d)** Distribution of behavioral measures of mice subdivided into three groups: sRC-members (blue), non-members (gray), and OXTRΔAON mice (red). sRC and non-members here only show control mice, so that no animal is represented more than once across groups. Normalized density distributions of within-group behavioral ranks (1 = highest activity) based on **c)** the fraction of total chases initiated by mice across the three groups and **d)** the fraction of total chases received across the three groups. All densities are normalized (area under each curve = 1) to allow comparison across groups with unequal sample sizes. Both in c) and d) the distributions of events of sRC members (blue) were different both from non-members (gray) and OXTR$^{\Delta AON}$ mice (red) ($p < 0.01$, two-sided permutation test on the median, 10000 iterations). **e)** Directionality of chases performed by sRC members.

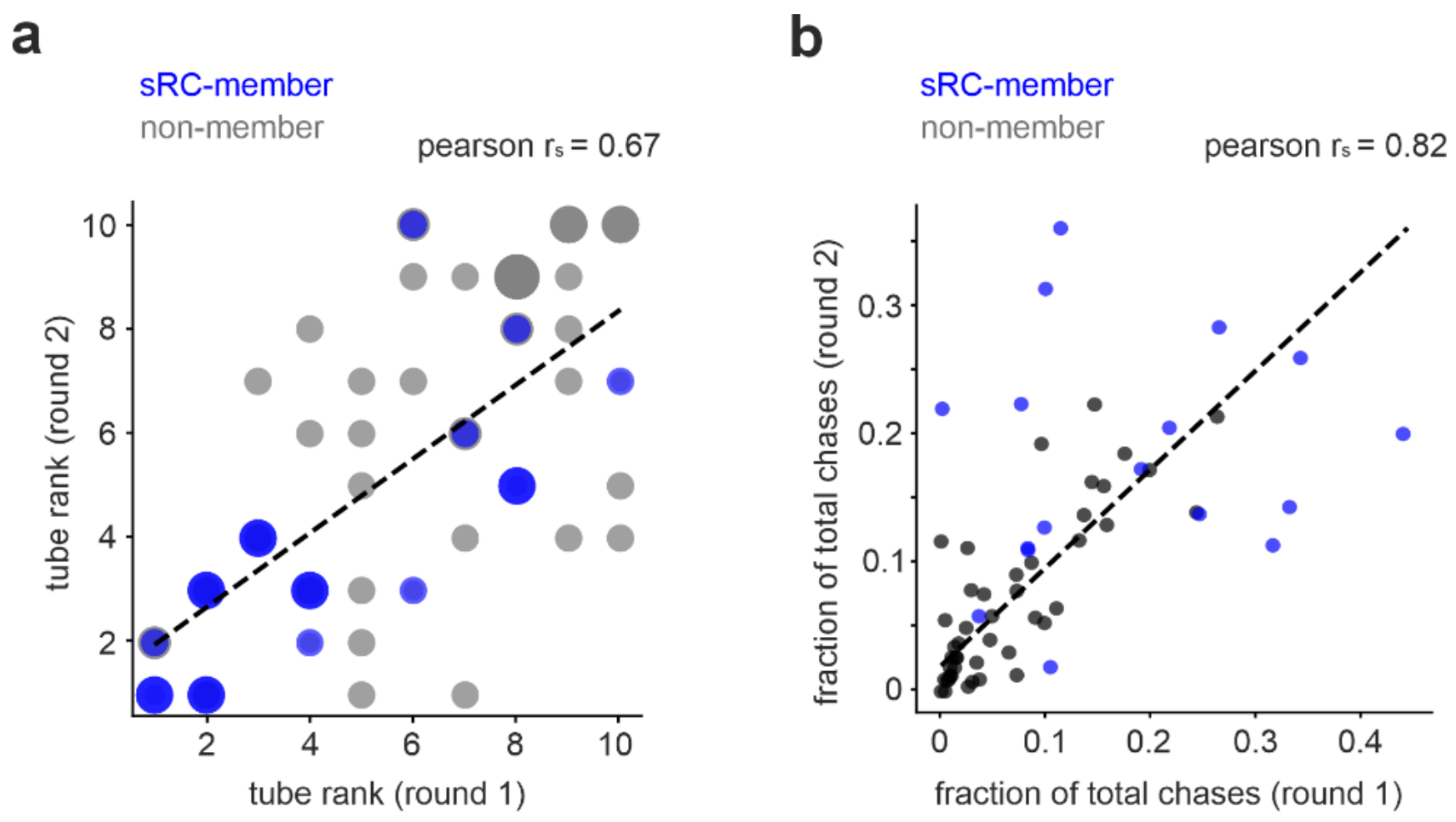

Supplementary Fig. 13: Stability of tube-test rank and chasing behavior across rounds.
a) Tube-test rank of mice in their first appearance in a NoSeMaze group plotted against their rank after reshuffling into a different group at a later round. sRC-members had to be in a sRC in at least one of the rounds. Non-members were not in a sRC in neither of the rounds. Dot size reflects the frequency of a given value combination across rounds. While a clear overall correlation is observed, no significant difference was found between correlations of sRC members and non-members. **b)** Fraction of initiated chases in the first roundcompared to the second after reshuffling. sRC-members had to be in a sRC in at least one round. Non-members were not in a sRC in neither of the rounds. A moderate overall correlation is observed, independent of sRC membership.

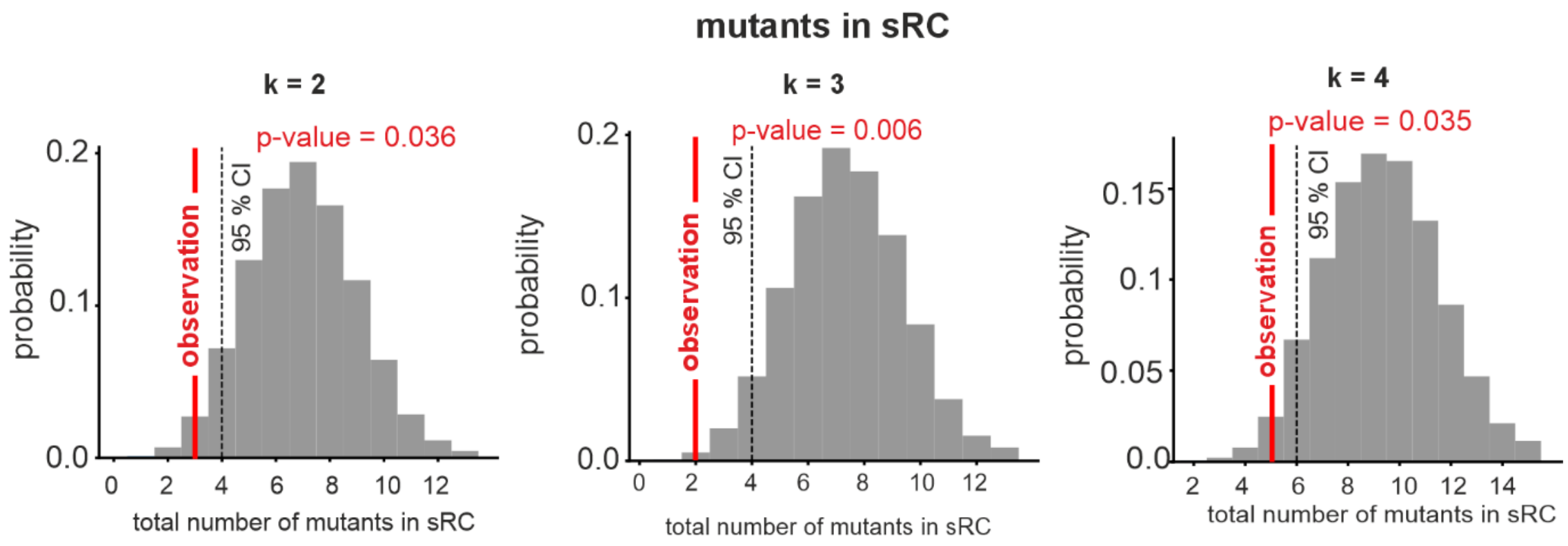

Supplementary Fig. 14: Robustness of stable rich-club composition across different pruning parameters, assessed via permutation tests.

Observed total number of OXTR$^{\Delta AON}$ mice within sRCs compared to a null distribution generated by random assignment. Across all pruning levels, observed frequencies (red lines) were significantly lower than expected by chance (all $p < 0.05$), indicating that OXTR$^{\Delta AON}$ mice are consistently underrepresented in sRCs. For details on the three permutation procedures, see Methods.

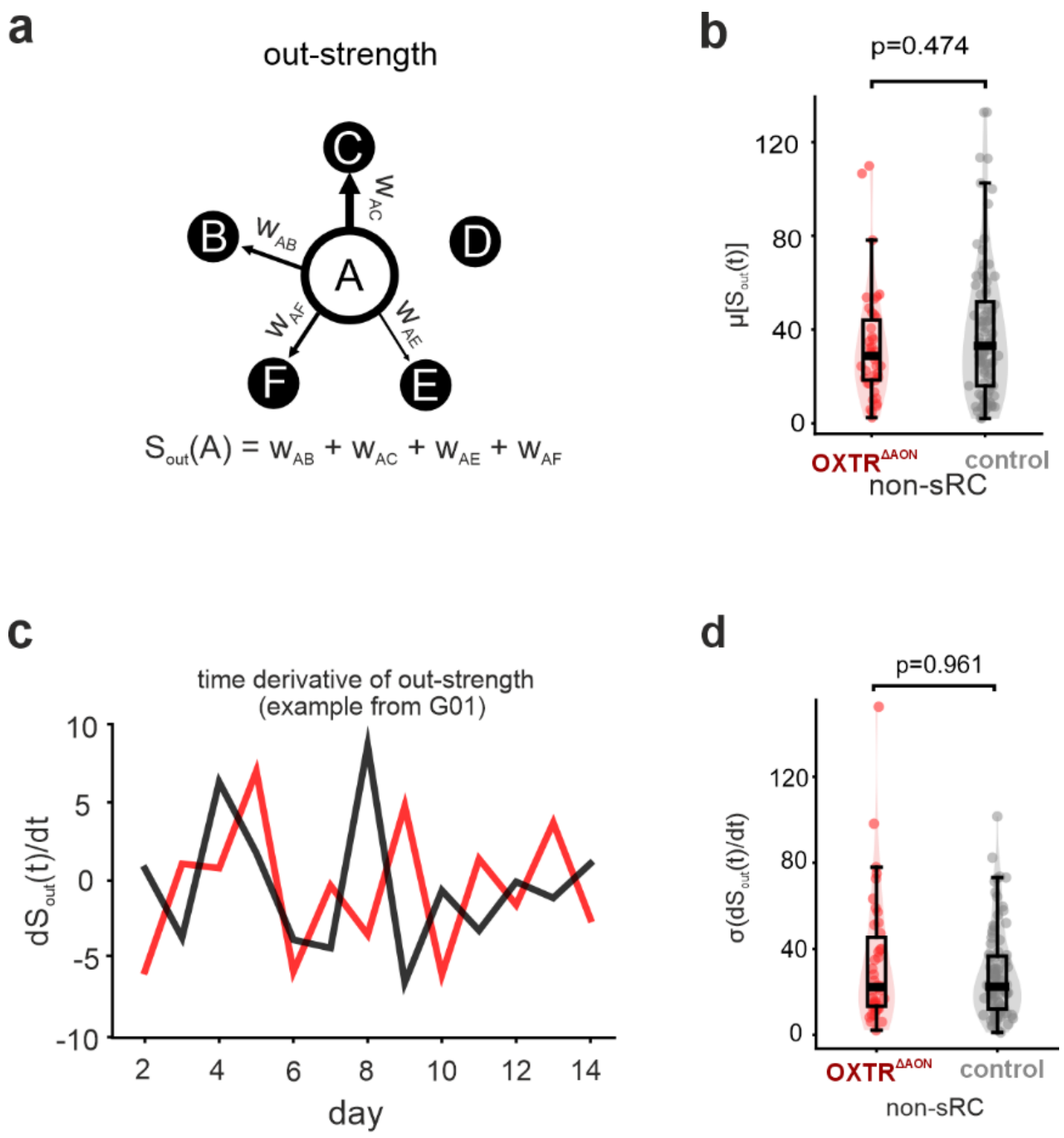

Supplementary Figure 15: Outgoing strength

a) Schematic illustrating outgoing strength (S_{out}), calculated as the sum of weighted edges from a node to all others. **b)** Mean S_{out} did not differ significantly between OXTR$^{\Delta AON}$ mice and control non-sRC-members. **c)** Example time derivatives of S_{out} from an OXTR$^{\Delta AON}$ mouse (red) and a control (dark gray) mouse from the same group. **d)** Standard deviation of the time derivative of S_{out}, used to assess fluctuations. Because S_{out} reflects overall social output of a given mouse, it can be viewed as a proxy for motivation to initiate social interactions. The absence of differences between OXTR$^{\Delta AON}$ mice and control non-members indicates that OXTR$^{\Delta AON}$ mice did not show impaired motivation during the experiment (two-sided permutation test on the median, 10000 iterations).

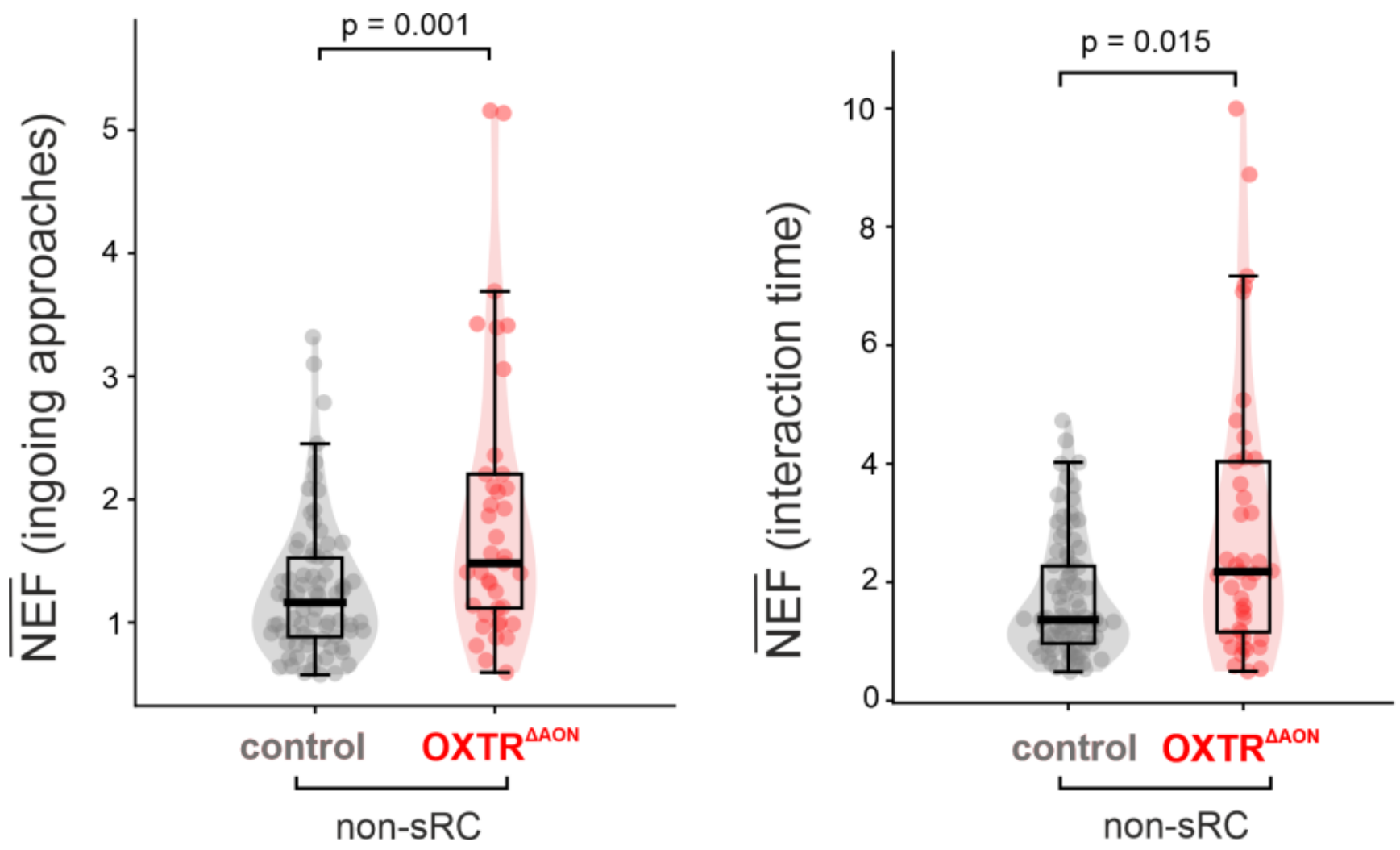

Supplementary Figure 16: Mean NEF for ingoing approaches and time spent interacting
Averaged NEF based on (left) ingoing approaches and (right) total interaction time (daily time spent interacting). $OXTR^{\Delta AON}$ mice showed significantly stronger temporal fluctuations in both ingoing approaches and daily time spent interacting than non-sRC-member control mice. Statistical significance is shown via the p-values displayed above their respective box plots (two-sided permutation test on the ranks, 10,000 iterations). Each dot represents one animal in one round.

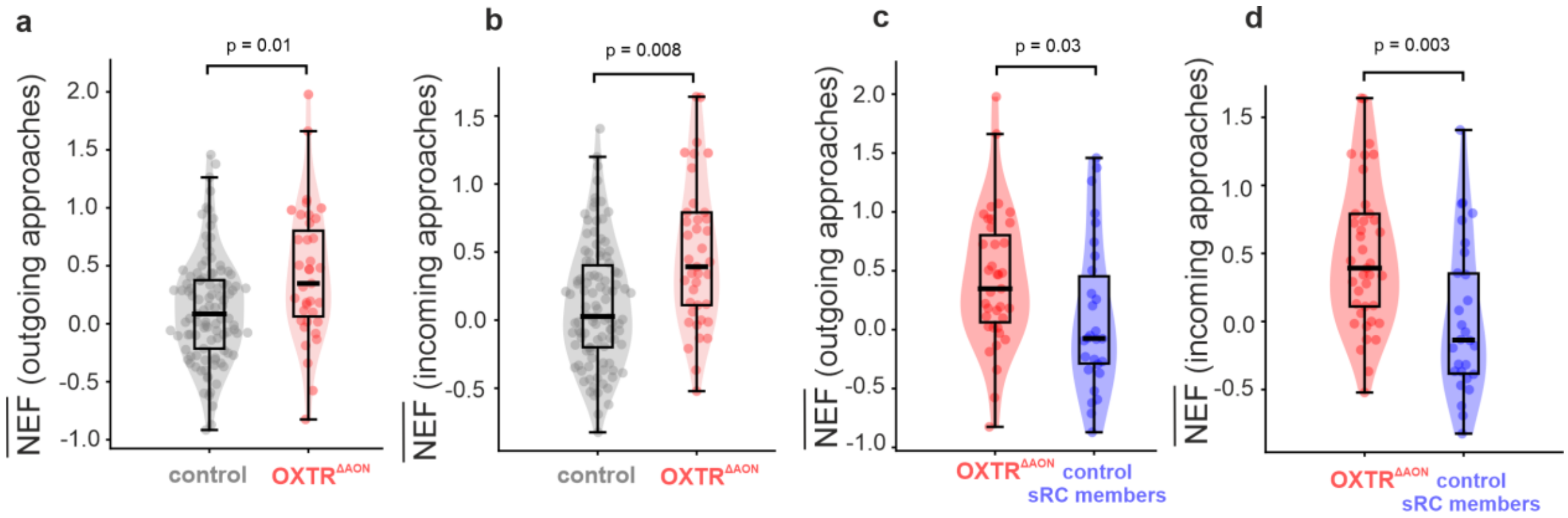

Supplementary Figure 17: Mean NEF for approaches of OXTR$^{\Delta AON}$ mice compared to all controls and sRC controls

Behavioral fluctuation metric (NEF) of OXTR$^{\Delta AON}$ mice compared to two groups: all control mice irrespective or sRC membership (gray), and control sRC members (blue). Comparison of the mean averaged NEF values between OXTR$^{\Delta AON}$ mice and all controls are shown for the **a)** outgoing and **b)** ingoing direction, respectively. Similarly, comparison of the mean averaged NEF values between OXTR$^{\Delta AON}$ mice and all control sRC members are shown for the **c)** outgoing and **d)** ingoing direction, respectively. OXTR$^{\Delta AON}$ mice showed significantly higher NEF values than controls or sRC members in both the outgoing (initiating) and incoming (receiving) direction (two-sided permutation test on the median, 10,000 iterations), indicating less consistent and more irregular social interactions. Statistical significance is shown via the p-values displayed above their respective box. For ease of readability, the axes are on a logarithmic scale.

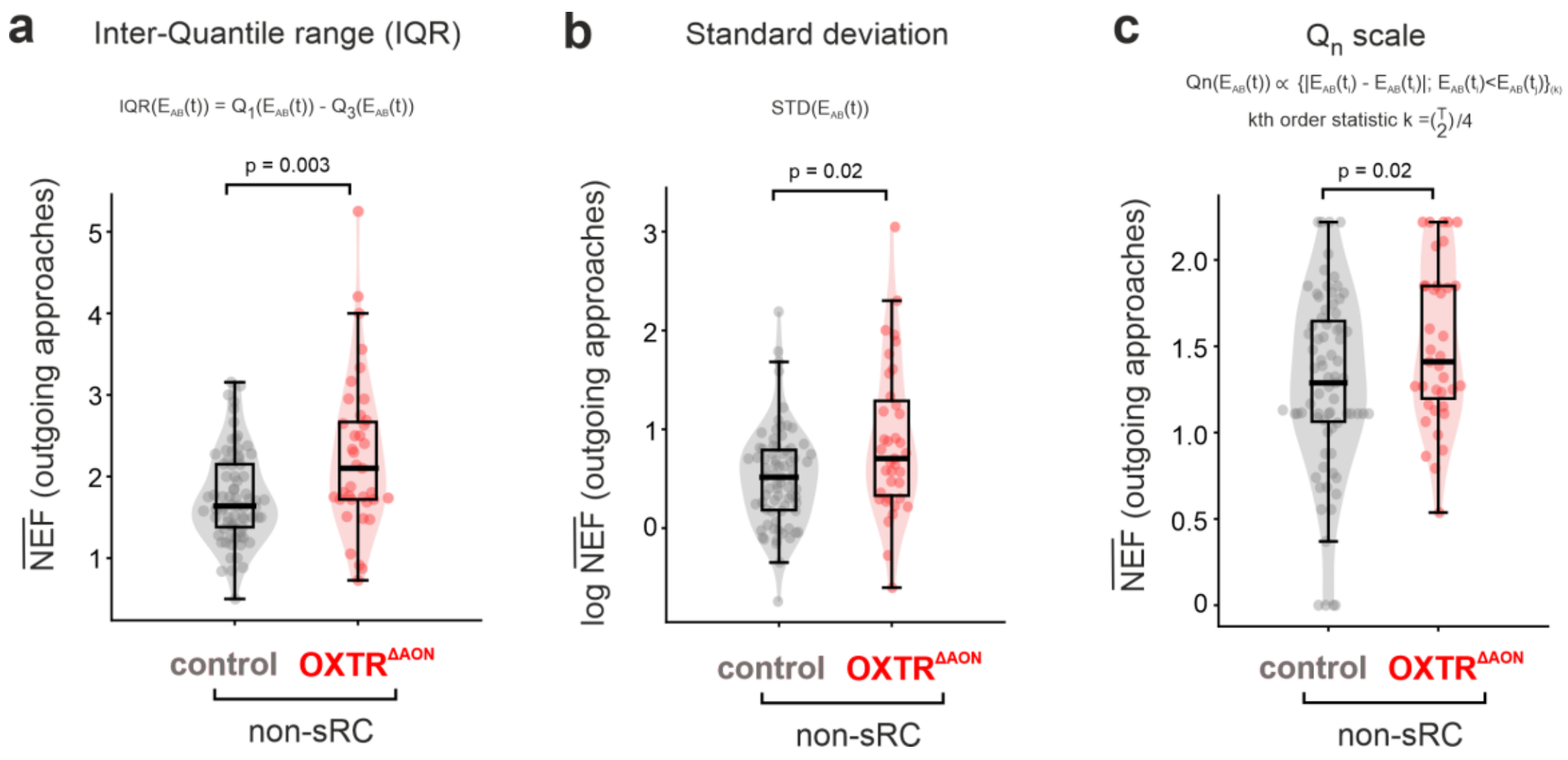

Supplementary Figure 18: Alternative expressions for the fluctuation term in the NEF

Alternative expression for the NEF. Since the NEF is quantifying the overall edge fluctuations normalized by "typical" activity level (denoted as *fluctuations* over *norm* in the main Fig. 8 and methods section), the formula given in the main text is not the only possible way to construct this measurement. Here we show how the NEF of non-sRC control mice compared to $OXTR^{\Delta AON}$ mice changes with different ways of quantifying edge fluctuations. In these three examples, the normalization factor used in the denominator is the median. For all possible alternative formulation of the NEF, we observe significant differences between non-sRC animal compared to $OXTR^{\Delta AON}$ mice (two-sided permutation test on the ranks, 10,000 iterations). In these plots, the approach distance threshold was set to 3. **a)** NEF computed using the inter-quantile range to estimate fluctuations. The inter quantile range of an edge is difference between the value of the first quartile (Q1) of the edge's values and its 3rd quartile (Q3). **b)** NEF computed using the standard deviation of the edge values. **c)** NEF computed using the Q_n robust scale estimator, which is designed to handle asymmetric data distribution and be robust against outliers.

Supplementary Tables

Supplementary Table S1: Overview of group compositions and animal information.
$OXTR^{\Delta AON}$, bilateral oxytocin receptor deletion in the AON pars centralis; RFID, radio-frequency identification; CON, control mouse

Mouse RFID	group	repetition	age	genotype	weight	Mouse RFID	group	repetition	age	genotype	weight
0007AC2B02	1	1	55	CON	33.59	0007CB0A48	12	1	18	CON	28.38
0007CB0942	1	1	55	CON	34.36	0007CB0AD4	12	1	16	CON	23.28
0007CB44CE	1	1	55	CON	25.92	0007CB2ED2	12	1	16	CON	26.51
0007CB7051	1	1	56	CON	32.58	0007CB321B	12	1	18	CON	26.61
0007CD6778	1	1	57	$OXTR^{\Delta AON}$	36.42	0007CB39C9	12	1	18	$OXTR^{\Delta AON}$	23.49
0007CD765E	1	1	57	CON	31.68	0007CB464C	12	1	16	$OXTR^{\Delta AON}$	27.25
0007CD77C5	1	1	57	$OXTR^{\Delta AON}$	32.71	0007F2B770	12	1	17	$OXTR^{\Delta AON}$	27.57
0007CDADED	1	1	55	CON	36.89	0007F2B957	12	1	18	$OXTR^{\Delta AON}$	28.45
0007CDC443	1	1	55	CON	36.46	0007F2BA9B	12	1	18	CON	26.04
0007CDDBAF	1	1	57	CON	31.46	0007F2CA05	12	1	18	$OXTR^{\Delta AON}$	25.31
0007CB2224	2	1	58	CON	35.99	0007CA3B1C	13	1	17	CON	24.43
0007CB3491	2	1	56	CON	33.91	0007CB1E1F	13	1	18	CON	28.15
0007CB4837	2	1	56	$OXTR^{\Delta AON}$	40.03	0007CB2183	13	1	17	$OXTR^{\Delta AON}$	25.32
0007CB6AB4	2	1	56	CON	27.19	0007CB2277	13	1	18	$OXTR^{\Delta AON}$	26.24
0007CD5116	2	1	57	CON	36.69	0007CB3D9B	13	1	18	CON	24.12
0007CDE223	2	1	75	CON	33.16	0007CB3F5E	13	1	18	CON	25.26
0007CDE4F0	2	1	58	$OXTR^{\Delta AON}$	30.71	0007CB6BC3	13	1	16	CON	23.81
0007CDEAA7	2	1	57	CON	36.04	0007F2945F	13	1	18	CON	23.81
0007CDEC0B	2	1	75	CON	32.32	0007F2B8A6	13	1	17	$OXTR^{\Delta AON}$	24.56
0007CDEEE2	2	1	75	CON	32.92	0007F2E455	13	1	18	$OXTR^{\Delta AON}$	24.72
0007CB0942	3	2	59	CON	34.47	0007CB0CDD	14	1	16	CON	23.82
0007CD38E5	3	1	59	CON	35.51	0007CB0F67	14	1	16	$OXTR^{\Delta AON}$	27.03
0007CD7FB2	3	1	63	$OXTR^{\Delta AON}$	34.07	0007CB48B4	14	1	16	CON	27.09
0007CD81D3	3	1	59	CON	37.51	0007CB6DFA	14	1	16	$OXTR^{\Delta AON}$	24.81
0007CD86B3	3	1	78	CON	32.71	0007F29741	14	1	17	CON	25.78
0007CDAD6B	3	1	78	CON	28.63	0007F2B88A	14	1	17	CON	24.38
0007CDC395	3	1	63	$OXTR^{\Delta AON}$	34.19	0007F2C687	14	1	18	CON	26.08
0007CDC91B	3	1	78	CON	31.69	0007F2EEFA	14	1	17	CON	24.78
0007CDDBAF	3	2	61	CON	32.41	0007F2F26A	14	1	18	CON	25.74
0007CE0327	3	1	78	CON	29.83	0007F2F283	14	1	16	$OXTR^{\Delta AON}$	24.07
0007ABF555	4	1	62	$OXTR^{\Delta AON}$	32.48	0007CA3A72	15	2	22	$OXTR^{\Delta AON}$	26.26
0007AC2B02	4	2	60	CON	33.03	0007CB0B74	15	2	22	CON	27.02
0007ACA781	4	1	79	CON	32.69	0007CB0DBC	15	2	22	$OXTR^{\Delta AON}$	28.11
0007CB2224	4	2	62	CON	35.46	0007CB2407	15	2	22	$OXTR^{\Delta AON}$	24.43
0007CB7051	4	2	61	CON	32.78	0007F2B3FF	15	2	22	CON	26.18
0007CD6778	4	2	62	$OXTR^{\Delta AON}$	38.56	0007F2B59E	15	2	20	CON	28.52
0007CD765E	4	2	62	CON	30.89	0007F2B8A2	15	2	22	$OXTR^{\Delta AON}$	26.42
0007CDADED	4	2	60	CON	37.41	0007F2B9FC	15	2	22	CON	26.52
0007CDC443	4	2	60	CON	37.22	0007F2E37E	15	2	22	CON	28.04
0007CDEAA7	4	2	60	CON	35.07	0007F2E3D4	15	2	22	CON	26.56
0007CB0942	5	3	62	CON	34.51	0007CA3B1C	16	2	21	CON	26.49
0007CB3491	5	2	62	CON	32.79	0007CB1E1F	16	2	22	CON	28.75
0007CB4837	5	2	62	$OXTR^{\Delta AON}$	36.62	0007CB2183	16	2	21	$OXTR^{\Delta AON}$	25.83
0007CD72E3	5	1	81	CON	35.94	0007CB2277	16	2	22	$OXTR^{\Delta AON}$	27.41
0007CD77C5	5	2	64	$OXTR^{\Delta AON}$	32.59	0007CB3D9B	16	2	22	CON	25.72
0007CDDBAF	5	3	64	CON	31.31	0007CB3F5E	16	2	22	CON	26.67
0007CDE223	5	2	81	CON	31.39	0007CB6BC3	16	2	20	CON	26.87
0007CDEC0B	5	2	81	CON	31.29	0007F2945F	16	2	22	CON	24.34
0007CDEEE2	5	2	81	CON	31.82	0007F2B8A6	16	2	21	$OXTR^{\Delta AON}$	26.76
0007CE0327	5	2	81	CON	28.41	0007F2E455	16	2	22	$OXTR^{\Delta AON}$	25.24
0007CB6AB4	6	2	73	CON	29.18	0007CB0CDD	17	2	20	CON	25.19
0007CB7051	6	3	74	CON	33.63	0007CB0F67	17	2	20	$OXTR^{\Delta AON}$	29.21
0007CD5116	6	3	74	CON	31.61	0007CB48B4	17	2	20	CON	26.15
0007CD765E	6	3	75	CON	31.39	0007CB6DFA	17	2	20	$OXTR^{\Delta AON}$	25.86
0007CD86B3	6	3	91	CON	32.58	0007F29741	17	2	21	CON	25.49
0007CDAD6B	6	2	91	CON	27.82	0007F2B88A	17	2	21	CON	25.22
0007CDADED	6	3	73	CON	36.98	0007F2C687	17	2	22	CON	25.96
0007CDC395	6	2	77	$OXTR^{\Delta AON}$	32.67	0007F2EEFA	17	2	21	CON	25.61
0007CDC443	6	3	73	CON	34.07	0007F2F26A	17	2	22	CON	26.72
0007CDE4F0	6	2	75	$OXTR^{\Delta AON}$	30.01	0007F2F283	17	2	20	$OXTR^{\Delta AON}$	25.74
0007ABF555	7	2	68	$OXTR^{\Delta AON}$	31.61	0007CA3B1C	18	3	26	CON	27.78
0007ACA781	7	2	85	CON	31.38	0007CB0AD4	18	3	25	CON	23.92
0007CB2224	7	3	68	CON	35.72	0007CB0F67	18	3	25	$OXTR^{\Delta AON}$	29.38
0007CD38E5	7	2	66	CON	34.85	0007CB2183	18	3	26	$OXTR^{\Delta AON}$	26.82
0007CD72E3	7	2	85	CON	34.39	0007CB2407	18	3	27	$OXTR^{\Delta AON}$	25.84
0007CD7FB2	7	2	70	$OXTR^{\Delta AON}$	33.79	0007CB3F5E	18	3	27	CON	29.41
0007CD81D3	7	2	66	CON	34.29	0007CB48B4	18	3	25	CON	27.93
0007CDEAA7	7	3	66	CON	34.37	0007F2BA9B	18	3	27	CON	28.36
0007CDEEE2	7	3	85	CON	30.45	0007F2E3D4	18	3	27	CON	28.97
0007CE0327	7	3	85	CON	29.61	0007F2E455	18	3	27	$OXTR^{\Delta AON}$	26.29
0007CA3A3A	8	1	68	CON	25.47	0007CB0AD4	19	2	20	CON	22.81
0007CB0942	8	4	68	CON	34.24	0007CB2ED2	19	2	20	CON	30.42
0007CB30A6	8	1	72	$OXTR^{\Delta AON}$	30.41	0007CB321B	19	2	22	CON	26.19
0007CB486D	8	1	71	$OXTR^{\Delta AON}$	31.92	0007CB39C9	19	2	22	$OXTR^{\Delta AON}$	26.18
0007CD5116	8	2	69	CON	32.67	0007F2B770	19	2	21	$OXTR^{\Delta AON}$	27.57

Mouse RFID	group	repetition	age	genotype	weight
0007CD86B3	8	2	87	CON	32.68
0007CDC91B	8	2	87	CON	32.73
0007CDDBAF	8	4	70	CON	32.51
0007CDE223	8	3	87	CON	33.11
0007CDEC0B	8	3	87	CON	31.56
0007CB2224	9	4	73	CON	36.15
0007CB2302	9	1	71	$OXTR^{\Delta AON}$	28.67
0007CB3713	9	1	71	CON	32.15
0007CB40DE	9	1	71	CON	25.89
0007CB47A6	9	1	72	CON	36.48
0007CB486D	9	2	74	$OXTR^{\Delta AON}$	30.16
0007CDEC0B	9	4	90	CON	31.29
0007CDEEE2	9	4	90	CON	31.51
0007CE0327	9	4	90	CON	29.14
0007CA3A3A	10	2	75	CON	25.77
0007CB0942	10	5	75	CON	35.77
0007CB2302	10	2	75	$OXTR^{\Delta AON}$	28.45
0007CB30A6	10	2	79	$OXTR^{\Delta AON}$	29.28
0007CB3713	10	2	75	CON	31.29
0007CB47A6	10	2	76	CON	35.97
0007CDDBAF	10	5	77	CON	31.39
0007CDE223	10	4	94	CON	32.49
0007CDEC0B	10	5	94	CON	30.08
0007CE0327	10	5	94	CON	28.62
0007CA3A72	11	1	18	$OXTR^{\Delta AON}$	26.68
0007CB0B74	11	1	18	CON	27.92
0007CB0DBC	11	1	18	$OXTR^{\Delta AON}$	28.07
0007CB2407	11	1	18	$OXTR^{\Delta AON}$	24.12
0007F2B3FF	11	1	18	CON	25.38
0007F2B59E	11	1	16	CON	28.07
0007F2B8A2	11	1	18	$OXTR^{\Delta AON}$	25.69
0007F2B9FC	11	1	18	CON	25.71
0007F2E37E	11	1	18	CON	26.1
0007F2E3D4	11	1	18	CON	26.16
0007F2B957	19	2	22	$OXTR^{\Delta AON}$	30.19
0007F2BA9B	19	2	22	CON	26.18
0007F2CA05	19	2	22	$OXTR^{\Delta AON}$	25.88
0007F2E42C	19	1	22	CON	30.91
0007CB0DBC	20	3	27	$OXTR^{\Delta AON}$	30.82
0007CB1E1F	20	3	27	CON	30.65
0007CB321B	20	3	27	CON	28.21
0007F29741	20	3	26	CON	27.97
0007F2B770	20	3	26	$OXTR^{\Delta AON}$	29.14
0007F2B9FC	20	3	27	CON	27.96
0007F2CA05	20	3	27	$OXTR^{\Delta AON}$	26.27
0007F2E37E	20	3	27	CON	29.92
0007F2EEFA	20	3	26	CON	28.03
0007F2F283	20	3	25	$OXTR^{\Delta AON}$	27.64
0007CA3A72	21	3	27	$OXTR^{\Delta AON}$	27.51
0007CB2277	21	3	27	$OXTR^{\Delta AON}$	29.32
0007CB464C	21	3	25	$OXTR^{\Delta AON}$	28.02
0007F2B3FF	21	3	27	CON	27.05
0007F2B59E	21	3	25	CON	31.52
0007F2B88A	21	3	26	CON	28.08
0007F2B8A6	21	3	26	$OXTR^{\Delta AON}$	28.69
0007F2B957	21	3	27	$OXTR^{\Delta AON}$	31.32
0007F2C687	21	3	27	CON	27.81
0007F2F26A	21	3	27	CON	28.52

Supplementary Table S2: Overview of group characteristics and acquisition of behavioral data in the NoSeMaze.

ID, identity; NoSeMaze, non-invasive sensor rich maze; SD, standard deviation

group ID	n of mice	age (median, SD, in weeks)	tube data	stimulus-outcome learning data	video data
1	10	55.5, 0.99	acquired	acquired	acquired
2	10	57.5, 8.79	acquired	acquired	acquired
3	10	63, 9.07	acquired	acquired	acquired
4	10	61.5, 5.77	acquired	acquired	acquired
5	10	72.5, 9.62	acquired	acquired	acquired
6	10	74.5, 7.17	acquired	acquired	acquired
7	10	69, 9.20	acquired	acquired	acquired
8	10	71.5, 9.03	acquired	acquired	acquired
9	9	73, 9.06	acquired	acquired	insufficient quality
10	10	76.5, 8.78	acquired	acquired	acquired
11	10	18, 0.63	acquired	acquired	acquired
12	10	18, 0.95	acquired	missing	acquired
13	10	18, 0.81	acquired	missing	acquired
14	10	16.5, 0.82	acquired	missing	acquired
15	10	22, 0.63	acquired	missing	acquired
16	10	22, 0.71	missing	acquired	acquired
17	10	20.5, 0.82	acquired	acquired	acquired
18	10	26.5, 0.92	acquired	acquired	missing
19	10	22, 0.88	acquired	acquired	missing
20	10	27, 0.84	missing	acquired	missing
21	10	27, 0.84	missing	acquired	missing

Supplementary table S3

In the main text, we show the presence of stable rich-clubs (sRCs) in groups composed entirely of control mice to demonstrate their existence in semi-naturalistic environments. sRC members have a notably higher level of social activity, as seen by a significantly larger number of total interactions, outgoing and incoming approaches. The mean duration of the interactions that sRCs members are involved with does not differ from that of non-member mice. This indicates that sRC members, while having higher social activities, are performing typical interactions with their peers. The p-values are obtained with a permutation test on the ranks (10,000 permutations), following the procedure described in the methods section of the main text. The approaches were obtained using an approach distance threshold value of 3.0. The mean interaction duration is given in seconds.

Measure	Median		Mean		Inter-quantile range		p-value
Number of interactions	990	*480*	3460	*1130*	5991	*807*	**0.005**
Mean interaction duration	2.66	*2.43*	2.96	*2.56*	0.85	*0.68*	0.23
Outgoing approaches	193	*95*	583	*225*	851	*158*	**0.01**
Incoming approaches	204	*81*	744	*177*	1294	*176*	**0.007**

sRC members *non-members*

Supplementary Table S4: Comparison non-sRC Control vs non-sRC $OXTR^{\Delta AON}$ mice

In the main text, non-sRC Control and non-sRC $OXTR^{\Delta AON}$ mice are displayed together as one category compared to sRC mice (Fig. 5e-h). This choice is motivated by the absence of significant differences between non-sRC Control and non-sRC $OXTR^{\Delta AON}$ mice, which can be seen in this table. The p-values are obtained with a permutation test on the median (10,000 permutations), following the procedure described in the methods section of the main text. The approaches were obtained using an approach distance threshold value of 3.0. The mean interaction duration is given in seconds.

Measure	Median of Controls	Median of $OXTR^{\Delta AON}$	p-value
Number of interactions	2173	2293	0.817
Outgoing approaches	565	591	0.779
Incoming approaches	572	560	0.877
Mean interaction duration	4.96	4.84	0.527
Tube rank	6	6	1.0
Chasing fraction	6.2%	5.5%	0.831

Supplementary Table S5: Overview of group compositions and animal information for the wild-type groups without OXTR^{ΔAON} deletion.
OXTR^{ΔAON}, bilateral oxytocin receptor deletion in the AON pars centralis; RFID, radio-frequency identification.

Mouse RFID	group	repetition	age	strain	Mouse RFID	group	repetition	age	strain
0007CA3A35	1	1	14	C57Bl/6J	0007FA1E6A	3	1	22.29	C57Bl/6J
0007CB357C	1	1	14	C57Bl/6J	0007D10A52	3	1	22.29	C57Bl/6J
0007CB239E	1	1	14	C57Bl/6J	0007D105F0	3	1	22.29	C57Bl/6J
0007CA38FF	1	1	14	C57Bl/6J	0007D10363	3	1	22.29	C57Bl/6J
0007CB330D	1	1	14	C57Bl/6J	0007FA0E3B	3	1	22.29	C57Bl/6J
0007CB08A5	1	1	14	C57Bl/6J	0007FA1CAC	3	1	22.29	C57Bl/6J
0007CB4123	1	1	14	C57Bl/6J	0007CE5289	3	1	22.29	C57Bl/6J
0007CB0D91	1	1	14	C57Bl/6J	0007FA1D79	3	1	22.29	C57Bl/6J
0007CB1EC1	1	1	14	C57Bl/6J	0007D0FA14	3	1	22.29	C57Bl/6J
0007CB6EA3	1	1	14	C57Bl/6J	0007D1019A	3	1	22.29	C57Bl/6J
0007CB0ABC	1	1	14	C57Bl/6J	0007FA0A36	3	1	22.29	C57Bl/6J
0007CB42F2	1	1	14	C57Bl/6J	0007D0FD05	3	1	22.29	C57Bl/6J
0007CA3D87	2	1	14	C57Bl/6J	0007FA0733	4	1	25.14	C57Bl/6J
0007CB0FC2	2	1	14	C57Bl/6J	0007FA0C72	4	1	25.14	C57Bl/6J
0007CA3BEA	2	1	14	C57Bl/6J	0007FA4B1A	4	1	25.14	C57Bl/6J
0007CB2584	2	1	14	C57Bl/6J	0007FA0B38	4	1	25.14	C57Bl/6J
0007CB24AD	2	1	14	C57Bl/6J	0007FA1F28	4	1	25.14	C57Bl/6J
0007CB24DA	2	1	14	C57Bl/6J	0007FA010F	4	1	25.14	C57Bl/6J
0007CB3395	2	1	14	C57Bl/6J	0007FA55AE	4	1	25.14	C57Bl/6J
0007CB39B0	2	1	14	C57Bl/6J	0007FA0EC5	4	1	25.14	C57Bl/6J
0007CB2246	2	1	14	C57Bl/6J	0007FA1FB2	4	1	25.14	C57Bl/6J
0007CB6FE7	2	1	14	C57Bl/6J	0007FA04F5	4	1	25.14	C57Bl/6J
0007CB6A8C	2	1	14	C57Bl/6J	0007FA1EFB	4	1	25.14	C57Bl/6J
0007CB2275	2	1	14	C57Bl/6J	0007FA1B3E	4	1	25.14	C57Bl/6J
