# Supplementary material for: Stable clique membership in male mouse societies requires oxytocin-enabled social sensory states
Source: Nat Commun. 2026 Aug 17;17:8493. doi: 10.1038/s41467-026-76841-5 (PMC13484484; doi:10.1038/s41467-026-76841-5)
Supplement: Supplementary file 2 [file 41467_2026_76841_MOESM2_ESM.pdf]

nature portfolio

Peer Review File

Stable clique membership in male mouse societies requires oxytocin-enabled social sensory states

Corresponding Author: Dr Wolfgang Kelsch

This file contains all reviewer reports in order by version, followed by all author rebuttals in order by version.

Version 0:

Reviewer comments:

Reviewer #1

(Remarks to the Author)
The study "Stable clique membership in mouse societies requires oxytocin-enabled social sensory states" by Nelias and Ghanayem et al investigates social behavior in male mice using the "NoSeMaze"-setup. The researchers used video tracking and identification by RFID tags and visual markers to obtain behavioral measures such as approaches, interactions, and interaction durations to construct networks representing social ties between individual mice. Certain animals form tightly interconnected subgroups, so called "stable rich-clubs" which seemingly play an important role in maintaining a vital social structure within the groups. Interestingly oxytocin signaling in the olfactory cortex is playing a prominent role in this social behavior: significantly less mice with an induced depletion in that system were able to be among these stable rich clubs compared to genetically un-altered controls.
The study is written in a clear and concise manner making it accessible not only to readers familiar with the specific field of research. The introduction sets the stage for the study by providing background information and highlighting the significance of the research especially with regard to the value for studying models for autism spectrum disorder. The methodological section is understandable and, all in all, presented in a clear manner. Together with the supplements, it contains all the necessary information. The results are overall presented clearly including data visualization where applicable. Some parts of the result section are only comprehensible after reading the method section, I would suggest to double check for adapting this better to the (in my view awkward) style of the journal where the methods are presented at the end of the paper. In the discussion the implications of the results are interpreted without exaggeration. Overall, I think this work will make a valuable contribution to 'Nature communications'. I have a few specific comments that I have taken from my notes while reading the manuscript, with the corresponding sections and line numbers:

Title: According to the ARRIVE guidelines I would recommend to include the term "male mice" in the title or at least in the abstract to emphasize that only one sex was analyzed.

Abstract: The term "rich-clubs" might not be clear to all audience and it is used in different contexts (according to a quick Google search). As this is an important concept for this paper this needs a little bit more elaboration. If it does not fit in the abstract wording limits, rather try something less confusing i.e., "We show that small, stable groups emerge from the specific social configuration."

It might not be obvious that "mice with genetic perturbations" are conditional KO animals. I would suggest using "genetically modified mice" instead to emphasize that this is an experimental model rather than a natural occurring genetic mutation.

Line 153: I know that the format of 'Nature communications' of presenting the results before the method section is a challenge… However, in order to be better comprehensible a short elaboration of what is meant by "transitions from approach into sampling behaviors" is needed. In addition, I would rather use the term "sniffing" than "sampling" for that type social interaction of mice.

173: include "male" before "mice". How many replicates were done?

177: How many times was each individual being tested? How did you re-shuffle groups of male mice without them killing

each other?

186: RNN first time mentioned – please explain the abbreviation. Also, in fig 3 it is mentioned that tracking was done using a CNN (also not explained). Did you use both types of neural networks? The training procedure should also be mentioned in the methods section.

341 It is more appropriate to use "male mice" instead of "mice."

351 I am not aware that the concept of “stable rich clubs” has found prominent entry into behavioral ecology literature. The three publications (8, 13, 14) mentioned seem to focus more (solely?) on human networks. I find this point extremely interesting and would like to see the authors add a few more sentences on other animal populations. The phenomenon is very prominent, but is often described using other terms (i.e., "coalition" in chimps doi.org/10.1007/3-540-28277-7_6; "grooming clique" in baboons, "social cluster" in dolphins https://doi.org/10.1098/rstb.2006.1997; types of relationships described for in ravens doi.org/10.1016/j.anbehav.2010.01.008; …).

481 I would recommend adding a sentence about how your sample size was estimated (ARRIVE guidelines).

544 Neither 38 nor 41 can be divided by 4 as in "group-housed (4 per cage)".

549 I am missing a brief description of how you dealt with aggressive behavior. Were there any serious injuries that resulted in animals having to be removed from the experiment? I am asking this also because the authorities responsible for granting approval are increasingly questioning this point. It is therefore also important to know if no such problems arose.

570 Refer to the type of fur bleaching patterns to figure 3 (also in the supplements).

577 typo: "to the whether an animal"

784 I can't find the LME designs in the respective method sections.

(Remarks on code availability)

Reviewer #2

(Remarks to the Author)
The article addresses one of the most important and still poorly understood questions in behavioral neuroscience: how mice, the dominant model for human brain disorders, form complex, stable social relationships inside groups rather than just engaging in isolated dyadic interactions.
For decades, laboratory mouse social behavior was treated as simple and largely irrelevant to understanding real social life. This work explicitly challenges that view by showing that mice living in large, semi-naturalistic groups self-organize into stable social cliques with reciprocity and influence, closely resembling the structure of human social networks.
Further, the study is highly timely. Across neuroscience, psychiatry, and computational neuroscience, there is now a strong push to move beyond single-pair interaction assays and instead understand social behavior as an emergent network phenomenon. This paper sits at that frontier by combining long-term tracking of large mouse groups, network theory, and circuit-specific manipulations.
A particularly strong aspect of this work is its treatment of mouse social systems as dynamic, complex networks rather than collections of independent individuals. The paper convincingly shows that perturbations in a single animal do not remain local but cascade through the network, reshaping group-level structure and stability. This network-level amplification of individual differences is en vogue in contemporary social neuroscience and deserves an even stronger emphasis in the framing and interpretation of the results. Authors may consider putting the relevant paragraph as an opening one in the Intro, to enhance the framing of their most notable results.
Further, by embedding a minority of impaired mice inside mostly neurotypical groups, the study shows how network dynamics amplify small individual deficits into large social exclusion, a mechanism that is almost impossible to see in traditional dyadic tests. However, this approach lacks backing by the standard control group approach, and it poses challenges to data interpretation (see further comments).
Another major point to be further addressed, and in my view, refined by the authors, is that mice fail to join social rich clubs (RCs). In my opinion, additional work is required to support this claim (see the following comments). Notably, the authors claim to identify a neurobiological mechanism that is necessary for joining and maintaining RCs. Mice with disrupted oxytocin signaling specifically in the anterior olfactory nucleus (AON) - OXTRΔAON interact normally and show normal social motivation, yet they seem to show unstable targeting of social partners over time. However, I believe the current analysis and data (as shown) are insufficient to convince the reader that it is the case.

Things to consider to improve the paper:
MAJOR
- Referring to mouse cliques as “rich-clubs” is potentially misleading. While the term is well-established in network science to denote highly interconnected nodes in the neural and behavioral context (e.g., van den Heuvel and Sporns, 2011; and other later influential works), it is commonly interpreted in social contexts as implying high status or preferential access to resources. If the authors wish to retain this terminology, they should explicitly clarify in the Intro that in this paper “rich-club” refers strictly to network connectivity and reciprocity (this is incoherent throughout the paper), not to social rank or material

advantage, to avoid conceptual confusion.
- The PVN optogenetic manipulation and the AON OXTR del address different levels of the OXY system and are therefore not directly comparable. Presenting them within a single figure and paragraph makes the experimental logic harder to follow and a bit forced, like putting together two sets of experiments not clearly connected from the get-go. Separating them conceptually (and possibly visually) would clarify what each manipulation is designed to test. It would also help if the Results explicitly stated - in one or two sentences - what behavioral context Fig. 1 reflects (isolated dyadic assay vs homecage setting), rather than leaving this information to the Methods only.
- The definition of an “approach” is unclearly phrased in the Results text. From the current description, it is not immediately clear whether approaches can be sequentially bidirectional (if the definition for the proportion of distance traveled is met). In addition, the rationale for the 1.5 distance ratio threshold is not justified. Because this variable underlies all directed network analyses, both the wording and the justification should be made more explicit in the Results, not only under the Fig. and in the Methods.
- The sparse inclusion of OXTRΔAON mice within mostly neurotypical groups is an interesting and ecologically relevant design. However, the current experiments lack a classic group-level control consisting of wild-type-only groups. The authors themselves explicitly argue that individual-level perturbations propagate through the social network and reshape group behavior. Thus, the presence of mutants itself becomes a group-level manipulation. Control groups without any mutants are therefore needed to establish a proper baseline for interpreting these network effects, or at a bare minimum, this limitation should be explicitly acknowledged. However, I believe compromising on this issue would significantly deplete the impact of the paper. I thus encourage the authors to think about including the conventional control groups as a baseline reference for their intriguing results.
- The RC are central to the paper, yet their quantitative definition is not clearly stated in the Results. The criteria used to identify RCs should be briefly but explicitly defined here, not only put in the Methods, for readers' benefit.
- For Fig. 4, it would be more informative to present 4 experimental groups rather than 3: RC controls, non-RC controls, RC OXTRΔAON, and non-RC OXTRΔAON. This would allow a clearer separation of network/genotype effects. The number of OXTRΔAON mice in the experiments may be too small for such stratification, but if so, this should be openly stated. This has a significant impact on the interpretation of the results, and it is unclear to me why the authors decided not to present the data in this way from the beginning. If there is an justification of that fact it should be presented to the reader.
- Line 259: “sRC members tended to occupy higher ranks in the social hierarchy, which was quantified by incidental tube-test competitions within the NoSeMaze (Fig. 5d-e, 260 Supplementary Fig. 9b).”This cannot be unequivocally concluded given the applied data presentation; the notion requires additional statistical support.
- The conclusion that OXTRΔAON mice fail to enter RCs seems to be difficult to evaluate, given the low number of the mutant animals overall in comparison to the WT mice. I would recommend that the authors additionally support this claim by reporting and comparing the probability of RC membership for (i) the general tested population, (ii) wild-type mice, and (iii) mutant mice. Not only the comparison between the general population/wt and the mutant mice, which I reckon is the analysis right now (Fig. 6a): I am unsure which one it is, since the presentation of this result is challenging for the reader, and the description is insufficiently clear for me to be more definite. A more explicit description would greatly benefit the comprehensibility of these data. In my view, without this, it remains unclear whether the apparent exclusion of the OXTRΔAON mice reflects a biological effect or simply the sparse representation of mutants. This is another case where including or referencing wild-type-only control groups would greatly help disentangle genotype effects from group-composition effects. Especially given the conclusions of the authors, such as “the overall strength of outgoing approaches fluctuated to a similar degree in mut and wt mice (line 310)”.
- Also, using NEF as the measure of social stability is an exciting concept, and I believe showing its application in the manuscript more broadly and explicitly would be beneficial to the field. Additionally, it might be interesting to the reader what the effects of applying this analytical approach to other measures of sociability, such as the time series of the time spent together in subsequent bins by the mice of diverse genotypes (since the OXTRΔAON mice do not differ from the wild-types when this variable is taken under consideration as a scalar value).
- The Discussion content (lines 397-421) should be rethought and rephrased as needed, if the conclusions regarding OXTRΔAON mice's ability to enter RCs is seen in a more comprehensive way in the light of new analysis (if performed).

MINOR:
- The claim (line 69) that difficulties in forming stable relationships become particularly relevant in adulthood is problematic. In ASD, the most severe functional impairments typically emerge in early childhood, when individuals learn to operate in structured social environments such as kindergarten and school; the current framing appears driven by the authors’ adult mouse model rather than by clinical reality. This statement should either be removed or explicitly reframed/cited to explain how adult de novo social integration deficits relate to early developmental social dysfunction.
- The intro paragraph (from line 91) already provides a nuanced account of oxytocin’s role in social behavior. To further strengthen it, I would recommend that the authors explicitly add (and cite) that oxytocin can promote not only affiliation but also aggression and other context-dependent “unpleasant” social responses, depending on the situation. Making this explicit would reinforce oxytocin as a complex, state-setting neuromodulator shaping multiple forms of social behavior, which aligns directly with the network-level framework of the paper.
- While dyadic assays (Fig. 1) are valuable for establishing baseline social interactions, dyadic behaviors outside a group are not equivalent to dyadic behaviors embedded in a social network (as the authors themselves argue). A brief acknowledgment of this in the Discussion would strengthen the interpretation of the following group-level results.
- Fig. 2b and c would benefit from being more explicit about the number of reshuffling rounds and how they were organized. In particular, the depiction of different age groups in the figure is not easily mapped onto the description in the Results. Aligning the figure more closely with the text and explaining the reshuffling structure more clearly would substantially improve readability.
- In Fig. 3, the rationale for giving panels g and h such prominent space is unclear. It would help the reader if the authors either justify their centrality or reduce it. Similarly, i and j seem to reiterate the same point, however, from slightly different

angles. In my view one should be put in the supplement; k is unclear on when the dark/light phase begins; also word "massive" in the figure caption seems obsolete.
- In Fig. 4, panels a and c are difficult to read in their current form. Presenting these panels as higher-quality graphics would substantially improve their interpretability and make the structure of the RC easier for the reader to follow
- This claim (line 228) "sRC emerged in all 10 rounds with older adults (Fig. 4c, Supplementary Fig. 5), while in younger adults they were observed in 4 of 7 rounds (Supplementary Fig. 5), potentially suggesting that age may favor clique formation." lacks the experimental support due to the insufficiency of the control conditions performed; I would advise softening it and further elaborating on it in the discussion.
- Chasing behavior in semi-naturalistic settings has been shown to correlate with social hierarchy/dominance (e.g., Winiarski et al., 2025). Given this, the way data is interpreted here should be more carefully contextualized. If the present results diverge from that established relationship, this discrepancy should be discussed.
- Fig. 6f: should be "unstable", not "instable"; also, Fig. 6 f-g should rather be the initial panels in this data illustration than shown at the end.
- This notion "(…) our findings support a non-hierarchical view of social brain functions, in which selective deficits in higher-order social functions can arise from alterations in early sensory processing rather than from dysfunction in higher brain regions. " presented in the discussion (line 393), and strongly supported by the presented data should be described in more depth, as it represents one of the most noteworthy discoveries presented in the paper.

Congratulations to the Authors on this intriguing and well-executed study. I am grateful for the opportunity to contribute to the improvement of this work. I remain at your disposal, as I believe this is a valuable and timely contribution to the field of social neuroscience. Alicja Puścian

(Remarks on code availability)
not applicable

Version 1:

Reviewer comments:

Reviewer #1

(Remarks to the Author)
All comments and concerns have been addressed in the revisions. I recommend publishing the article.

(Remarks on code availability)

Reviewer #2

(Remarks to the Author)
I believe that the authors have done a very good job addressing the comments. The new version of the ms is significantly clearer. Several of the corrections made the work stronger. Especially in terms of the presented rationale and the logic of the analyses.
Most notably, the authors improved the description of the algorithm used to quantify approach behavior, which lies at the foundation of their analysis of complex social behavior. This is an important correction. In the revised version, this measure is much more rigorous and clearly explained, which increases confidence in the analyses built on it.
Figures have also been significantly improved. Especially new figs. 3 and 5. They are now much more reader-friendly and make the logic of the results easier to follow. In addition, the authors performed new quantitative analyses, including new statistical testing, which further strengthens the conclusions.
The authors also improved the normalized edge fluctuation (NEF) calculations by changing the normalization strategy so that it better reflects behavioral fluctuations relative to the typical (rather than mean) activity levels of the animals. I see this as a significant improvement. The authors also applied it to new measures of social behavior in their experiments, which brought new insights, now presented in the Suppl figs. 17-19. NEF is, in my opinion, an exciting measure of variability that could be further applied to other experimental measures of sociability in the network context, beyond this work.
Further, the addition of new control experiments with WT mice was an indispensable element for empowering the conclusions of the paper. The fact that RCs were formed in 3 out of 4 cohorts is informative. It shows that the process is not universal, which is completely fine, as the social nature of the groups is understandably complex. However, I strongly believe that these results are critical to the understanding of the paper and should not remain in the supplementary material and cited in the main ms, which is how the authors decided to place it. They should be moved to the main figures. In my view, this is not a secondary control, but one of the main results on which the whole logic of the paper is built. I would also advise the authors to include in these fig. panels analogous to those currently shown in fig. 5e-h, to make the comparisons equivalent. In addition, a pie chart equivalent to the new (and very clear!) fig. 7a, but prepared for the control experiments with WT animals exclusively, would be a greatly valuable addition to this data presentation. Taken together, this would further help disentangle group effects from genotype effects, which I think is critically important, especially in face of inability to perform statistical tests allowing to disentangle the two, because of very few mutant animals in the RCs.
Provided this correction is made, I recommend this paper for publication. I believe it is a very valuable and well-thought-out

addition to our understanding of higher order group processes in rodent social groups. I congratulate the authors on the immense workload they put into this paper, as the effects of their work are highly noteworthy.
Alicja Puścian

(Remarks on code availability)

Open Access This Peer Review File is licensed under a Creative Commons Attribution 4.0 International License, which permits use, sharing, adaptation, distribution and reproduction in any medium or format, as long as you give appropriate credit to the original author(s) and the source, provide a link to the Creative Commons license, and indicate if changes were made.
In cases where reviewers are anonymous, credit should be given to 'Anonymous Referee' and the source.
The images or other third party material in this Peer Review File are included in the article's Creative Commons license, unless indicated otherwise in a credit line to the material. If material is not included in the article's Creative Commons license and your intended use is not permitted by statutory regulation or exceeds the permitted use, you will need to obtain permission directly from the copyright holder.
To view a copy of this license, visit https://creativecommons.org/licenses/by/4.0/

REVIEWER COMMENTS: Nelias, Ghanayem et al.

Reviewer #1

The study "Stable clique membership in mouse societies requires oxytocin-enabled social sensory states" by Nelias and Ghanayem et al investigates social behavior in male mice using the "NoSeMaze"-setup. The researchers used video tracking and identification by RFID tags and visual markers to obtain behavioral measures such as approaches, interactions, and interaction durations to construct networks representing social ties between individual mice. Certain animals form tightly interconnected subgroups, so called "stable rich-clubs" which seemingly play an important role in maintaining a vital social structure within the groups. Interestingly oxytocin signaling in the olfactory cortex is playing a prominent role in this social behavior: significantly less mice with an induced depletion in that system were able to be among these stable rich clubs compared to genetically un-altered controls.

The study is written in a clear and concise manner making it accessible not only to readers familiar with the specific field of research. The introduction sets the stage for the study by providing background information and highlighting the significance of the research especially with regard to the value for studying models for autism spectrum disorder. The methodological section is understandable and, all in all, presented in a clear manner. Together with the supplements, it contains all the necessary information. The results are overall presented clearly including data visualization where applicable. Some parts of the result section are only comprehensible after reading the method section, I would suggest to double check for adapting this better to the (in my view awkward) style of the journal where the methods are presented at the end of the paper. In the discussion the implications of the results are interpreted without exaggeration. Overall, I think this work will make a valuable contribution to 'Nature communications'.

We thank the Reviewer for this helpful comment, addressed these points and revised the manuscript to provide key technical information in the main results.

I have a few specific comments that I have taken from my notes while reading the manuscript, with the corresponding sections and line numbers:

- Title: According to the ARRIVE guidelines I would recommend to include the term "male mice" in the title or at least in the abstract to emphasize that only one sex was analyzed.

We added male to the title.

- Abstract: The term "rich-clubs" might not be clear to all audience and it is used in different contexts (according to a quick Google search). As this is an important concept for this paper this needs a little bit more elaboration. If it does not fit in the abstract wording limits, rather try something less confusing i.e., "We show that small, stable groups emerge from the specific social configuration."

We modified the abstract, focused on the term cliques and in this context explained the term rich club: starting L. 42 "*We show that small, stable cliques emerge from the specific group configuration. Consistently, also kinship did not significantly facilitate entry. These cohesive cliques resembled human mutually interacting rich-clubs, exhibiting high social rank and influence over non-members.*"

- It might not be obvious that "mice with genetic perturbations" are conditional KO animals. I would suggest using "genetically modified mice" instead to emphasize that this is an experimental model rather than a natural occurring genetic mutation.

Thank you. We modified this to: L. 48 *"…mice with conditional oxytocin receptor deletion in this sensory cortex …"*

- Line 153: I know that the format of 'Nature communications' of presenting the results before the method section is a challenge… However, in order to be better comprehensible a short elaboration of what is meant by "transitions from approach into sampling behaviors" is needed. In addition, I would rather use the term "sniffing" than "sampling" for that type social interaction of mice.

We now better explain in the results starting L. 149: "*It is not entirely known if loss of OXT signaling in the AON affects the microstructure of self-paced dyadic same-sex interactions. We therefore first analyzed video-tracked interaction states and the transitions in a simple condition of brief explorations of two mice in a cage suited for high-resolution mapping of exploration sequences (new Fig. 1a-b). Specifically, we quantified how often an animal progressed from approach (orientation and movement toward the partner within close range) to sniffing (close-contact investigatory olfactory behavior directed to the partner, including anogenital, nose-to-nose, and flank-sniffing), versus disengaging after initiating approach (i.e., aborted interactions).…"*

And starting L. 161 "*Boosted OXT release led to a tendency toward more sniffing events, with significantly shorter durations (new Fig. 1e). Boosted OXT release also increased the likelihood that approaches progressed into sniffing and reduced the probability that interactions were aborted after an approach was initiated (new Fig. 1f-g), consistent with enhanced social exploration.*"

Also, we replaced "sampling" by "sniffing" in the figure legends.

- 173: include "male" before "mice". How many replicates were done?

We added "male" in the text, now L. 185, and revised new Fig. 3b–d, together with the corresponding Results and Methods text, to clarify the experimental design. A detailed description of these procedures is provided in the next point.

- 177: How many times was each individual being tested? How did you re-shuffle groups of male mice without them killing each other?

To clarify the experimental design, we modified new Fig. 3b-d and its caption, adding details on the cohorts and the re-shuffling scheme. We now explicitly state the number of independent NoSeMaze rounds performed in each cohort (11 for the younger, and 10 for the older cohort), and we clarify that mice were tested repeatedly across multiple 3-week NoSeMaze rounds with different group compositions. Of the 21 NoSeMaze rounds conducted, 16 rounds (7 younger, 9 older) had complete video data and extraction of individual animal positions were consistently obtained with DLC to include them into the analysis.

The number of rounds per individual varied. We now clarify this in the text and provide the group assignments across rounds in Supplementary Table 1. We also expanded the description of the reshuffling procedure. Reshuffling was performed within age cohorts only. One possible explanation for the absence of overt aggression or injuries in the NoSeMaze is this stepwise familiarization.

In addition to the new Figure 3 and its caption, we modified the results section starting L. 188: *"To ensure that phenotypes were observed across the adult lifespan, we studied two age cohorts separately: younger (16–30 weeks; 41 mice, 16 OXTR$^{\Delta AON}$, 25 controls) and older adults (55–97 weeks; 38 mice, 10 OXTR$^{\Delta AON}$, 28 controls) (new Fig. 3b). Before entering the NoSeMaze, mice within each cohort were repeatedly reshuffled in home-cage groups of 3-5 animals (new Fig. 3c-d) to promote familiarity among future group members. This procedure may also have contributed to the lack of overt aggression or injuries in the NoSeMaze. Most mice participated in multiple rounds (see Supplementary Table S1). To assess whether social behaviors depended on specific group*

composition, mice lived with different group members in each round of the NoSeMaze ('reshuffling', new Fig. 3d).

We then established a pipeline to extract the social interaction networks from continuous video data in the open field arena for the groups of individually identified mice, including interspersed OXTR$^{\Delta AON}$ mutants (new Fig. 4a-b). To identify individual mice, distinct fur patterns were bleached onto the back of the mice before each round (new Fig. 4c). We analyzed the first 15 days of each round, as the subsequent regrowth of the fur compromised the reliability of pattern recognition by the trained Convolutional Neural Network (CNN). Before each new round, the fur patterns were refreshed. 16 out of 21 NoSeMaze rounds with interspersed OXTR$^{\Delta AON}$ mice yielded video datasets of sufficient quality for social network reconstruction and were included in the video-based analyses (Fig. 4a-b). Additionally, we analyzed four rounds with wild-type mice only (cf. New Supplementary Fig. 10), totaling overall to 7,200 hours of continuous video data used to generate the social networks in the final dataset (new Fig. 4d).“

We also updated the Methods section starting L. 603: *"Two to four parallel NoSeMaze units were used, each housing 9–10 mice for 3-week rounds. Each group included 2–5 OXTR$^{\Delta AON}$ mice (see Supplementary Table S1) interspersed among neurotypical controls, mimicking sparse genetic variation. Groups were reshuffled before NoSeMaze entry and again between rounds (see new Fig. 3c-d), with most mice participating in multiple rounds. Two separate cohorts, younger (16–30 weeks, n = 41, n = 16 OXTR$^{\Delta AON}$) and older (55–97 weeks, n = 38, n = 10 OXTR$^{\Delta AON}$) animals, were tested. During baseline housing (before and after surgery) and between the rounds in the NoSeMaze, animals were group-housed (3-5 mice per cage) under controlled environmental conditions (12:12 h light/dark cycle, 24 °C, 55% humidity) with unrestricted access to food and water. Familiarity with the other animals of each cohort was maximized by reorganizing group compositions every 3–4 days, ensuring each mouse interacted with all other cohort members prior to entering the NoSeMaze for the first time. For details on the NoSeMaze system and the habituation, see section 'NoSeMaze' in the Supplementary information."*

- 186: RNN first time mentioned – please explain the abbreviation. Also, in fig 3 it is mentioned that tracking was done using a CNN (also not explained). Did you use both types of neural networks? The training procedure should also be mentioned in the methods section.

Thank you for highlighting this. We consistently used DLC's ResNet-50. We changed in the main text RNN to "Convolutional Neural Network (CNN)" in L. 205.

We also added a more detailed description of the training procedure to the methods starting L.632: "*Videos were analyzed for all groups where video data was complete and of sufficient quality (see Supplementary Table S2) to train DeepLabCut (DLC) [47]. Separate DLC models based on the ResNet-50 architecture were trained for each group to detect the different fur bleaching patterns in the video frames, enabling individual identification and analysis of interaction behavior (cf. Fig. 4c-d). The training set consisted of manually labeled frames from videos recorded on days 1, 5, and 10. Each 24-hour video was divided into 5-minute segments, from which 30-minute clips were assembled based on maximal pixel variation to enrich for mouse activity. Frames were then extracted using DLC's k-means algorithm to select the most diverse samples. Mice were then manually labeled by trained experimenters based on their fur patterns, ensuring balanced representation of individuals in the training set."*

- 341 It is more appropriate to use "male mice" instead of "mice."

We added "male"

- 351 I am not aware that the concept of "stable rich clubs" has found prominent entry into behavioral ecology literature. The three publications (8, 13, 14) mentioned seem to focus more

(solely?) on human networks. I find this point extremely interesting and would like to see the authors add a few more sentences on other animal populations. The phenomenon is very prominent, but is often described using other terms (i.e., "coalition" in chimps doi.org/10.1007/3-540-28277-7_6; "grooming clique" in baboons, "social cluster" in dolphins https://doi.org/10.1098/rstb.2006.1997; types of relationships described for in ravens doi.org/10.1016/j.anbehav.2010.01.008; …).

We highlight this point in the introduction starting L. 80: "*Related clique structures exist in other social species, such as coalitions in chimpanzees, social clusters in dolphins, or relationships in ravens [16–18].*"

- 481 I would recommend adding a sentence about how your sample size was estimated (ARRIVE guidelines).

We added to the method section starting L. 859: "*Our primary outcomes are longitudinal network-derived measures (e.g., stable rich-club membership and edge-dynamics) from interdependent observations within reshuffled groups, for which standard closed-form a priori power calculations are not readily applicable. Sample size was therefore set by NoSeMaze capacity (up to four parallel units, 9–10 mice per 3-week round) and the design requirement to test the $OXTR^{\Delta AON}$ minority in group hypothesis (2–5 mutants per group).*"

- 544 Neither 38 nor 41 can be divided by 4 as in "group-housed (4 per cage)".

We thank the reviewer for this clarification. Before entering the NoSeMaze and between NoSeMaze rounds, mice were housed in standard group cages of typically 4 animals, with a small number of cages containing 3 or 5 animals as required by cohort size and husbandry logistics. We now provide a precise description starting L. 192 in the Results *"Before entering the NoSeMaze, mice within each cohort were repeatedly reshuffled in home-cage groups of 3-5 animals (new Fig. 3c-d) to promote familiarity among future group members."* and L. 610 in the Methods *"Before and after surgery and between the rounds in the NoSeMaze, animals were group-housed (3-5 mice per cage) under controlled environmental conditions (12:12 h light/dark cycle, 24 °C, 55% humidity) with unrestricted access to food and water."*

- 549 I am missing a brief description of how you dealt with aggressive behavior. Were there any serious injuries that resulted in animals having to be removed from the experiment? I am asking this also because the authorities responsible for granting approval are increasingly questioning this point. It is therefore also important to know if no such problems arose.

We now explain this point briefly in the result section starting L. 192: "*… Before entering the NoSeMaze, mice within each cohort were repeatedly reshuffled in home-cage groups of 3-5 animals (new Fig. 3c-d) to promote familiarity among future group members. This procedure may also have contributed to the lack of overt aggression or injuries in the NoSeMaze.*"

- 570 Refer to the type of fur bleaching patterns to figure 3 (also in the supplements).

We added a reference to new Fig. 4c in the respective places.

- 577 typo: "to the whether an animal"

Thank you, we corrected it.

- 784 I can't find the LME designs in the respective method sections.

We thank the reviewer for pointing this out. The LME models were only provided in the Supplementary Methods. We have now revised the main manuscript to explicitly direct readers to the Supplementary Methods for the full LME specification (Methods starting L. 590: *"The LME designs used for the statistical assessments of between group differences are reported in the Supplementary Methods ("Analysis of interaction videos"). Models were fitted in MATLAB."*).

In addition, we revised the Supplementary Methods to state the fixed and random effects explicitly and to provide example model formulas for both the state-based metrics and the transition analyses (Suppl Methods starting L. 79: *"Mean duration and frequency of interaction states, approach, and idle were compared using linear mixed-effect models (LME, MATLAB fitlme), with animal ID as a random effect to account for repeated measures. For the optogenetics cohort, the model was: metric ~ intervention + (1|animalID), where intervention = sham vs. oxytocin release. For the $OXTR^{\Delta AON}$ cohort, the model was: metric ~ group + (1|animalID), where group = control vs. $OXTR^{\Delta AON}$."*)

Reviewer #2

The article addresses one of the most important and still poorly understood questions in behavioral neuroscience: how mice, the dominant model for human brain disorders, form complex, stable social relationships inside groups rather than just engaging in isolated dyadic interactions.

For decades, laboratory mouse social behavior was treated as simple and largely irrelevant to understanding real social life. This work explicitly challenges that view by showing that mice living in large, semi-naturalistic groups self-organize into stable social cliques with reciprocity and influence, closely resembling the structure of human social networks.

Further, the study is highly timely. Across neuroscience, psychiatry, and computational neuroscience, there is now a strong push to move beyond single-pair interaction assays and instead understand social behavior as an emergent network phenomenon. This paper sits at that frontier by combining long-term tracking of large mouse groups, network theory, and circuit-specific manipulations.

A particularly strong aspect of this work is its treatment of mouse social systems as dynamic, complex networks rather than collections of independent individuals. The paper convincingly shows that perturbations in a single animal do not remain local but cascade through the network, reshaping group-level structure and stability. This network-level amplification of individual differences is en vogue in contemporary social neuroscience and deserves an even stronger emphasis in the framing and interpretation of the results. Authors may consider putting the relevant paragraph as an opening one in the Intro, to enhance the framing of their most notable results.

Further, by embedding a minority of impaired mice inside mostly neurotypical groups, the study shows how network dynamics amplify small individual deficits into large social exclusion, a mechanism that is almost impossible to see in traditional dyadic tests. However, this approach lacks backing by the standard control group approach, and it poses challenges to data interpretation (see further comments).

Another major point to be further addressed, and in my view, refined by the authors, is that mice fail to join social rich clubs (RCs). In my opinion, additional work is required to support this claim (see the following comments). Notably, the authors claim to identify a neurobiological mechanism that is necessary for joining and maintaining RCs. Mice with disrupted oxytocin signaling specifically in the anterior olfactory nucleus (AON) - OXTRΔAON interact normally and show normal social motivation, yet they seem to show unstable targeting of social partners over time. However, I believe the current analysis and data (as shown) are insufficient to convince the reader that it is the case.

We thank the Reviewer for her helpful comments and addressed them point-by-point.

MAJOR

- Referring to mouse cliques as “rich-clubs” is potentially misleading. While the term is well-established in network science to denote highly interconnected nodes in the neural and behavioral context (e.g., van den Heuvel and Sporns, 2011; and other later influential works), it is commonly interpreted in social contexts as implying high status or preferential access to resources. If the authors wish to retain this terminology, they should explicitly clarify in the Intro that in this paper “rich-club” refers strictly to network connectivity and reciprocity (this is incoherent throughout the paper), not to social rank or material advantage, to avoid conceptual confusion.

We thank the reviewer for highlighting this. We have clarified in the Introduction that “rich-club” is used strictly in the network-science sense (a connectivity-based topological property) to identify them. We further state that any relationship between rich-club membership and dominance-related variables is treated as a separate empirical question, which we address using independent measures (tube-test rank and chasing). We added starting L. 91: *“We adopt the definition of RCs from network science and use it here strictly as a connectivity-based term, i.e., a topological property describing preferential, reciprocal connectivity among highly connected individuals. Based on this formal description, we then can test whether membership in an RC is associated with other dominance-related behaviors.”*

- The PVN optogenetic manipulation and the AON OXTR del address different levels of the OXY system and are therefore not directly comparable. Presenting them within a single figure and paragraph makes the experimental logic harder to follow and a bit forced, like putting together two sets of experiments not clearly connected from the get-go. Separating them conceptually (and possibly visually) would clarify what each manipulation is designed to test. It would also help if the Results explicitly stated - in one or two sentences - what behavioral context Fig. 1 reflects (isolated dyadic assay vs homecage setting), rather than leaving this information to the Methods only.

We agree. While the PVN optogenetic stimulation mainly serves here to probe the sensitivity of the behavioral assay, adult AON OXTR deletion provides complementary information to the NoSeMaze on the phenotype at the level of microsequences. To improve clarity, we split the original Fig. 1 into two figures as suggested (new Fig. 1 and Fig. 2) and revised the corresponding Results text to treat them as two conceptually distinct manipulations.

We also added one explicit sentence in the Results to state that these figures reflect a simple dyadic assay, so the behavioral context is clear without relying on the Methods. Results section starting L. 150: "*We therefore first analyzed video-tracked interaction states and the transitions in a simple condition of brief explorations of two mice in a cage suited for high-resolution mapping of exploration sequences (new Fig. 1a-b). Specifically, we quantified how often an animal progressed from approach (orientation and movement toward the partner within close range) to sniffing (close-contact investigatory olfactory behavior directed to the partner, including anogenital, nose-to-nose, and flank-sniffing), versus disengaging after initiating approach (i.e., aborted interactions).*"

- The definition of an "approach" is unclearly phrased in the Results text. From the current description, it is not immediately clear whether approaches can be sequentially bidirectional (if the definition for the proportion of distance traveled is met). In addition, the rationale for the 1.5 distance ratio threshold is not justified. Because this variable underlies all directed network analyses, both the wording and the justification should be made more explicit in the Results, not only under the Fig. and in the Methods.

We thank the reviewer for this clarifying point. We defined an approach event (A→B) based on two criteria:

First, the movement episode had to end in a dyadic interaction lasting ≥ 1 s, to ensure that we quantify approach movements that lead into an actual interaction and to avoid counting unrelated locomotion. This also means that we consider both animals' approaches that end with an interaction, thereby precluding sequential approaches confounding the measure. (Bidirectional sequential approaches with an interaction after the first approach are counted as two events if the interaction criterion is met). If two mice move within the set time period before the interaction towards each other, the asymmetry in the distance traveled (threshold ratio detailed further below) asserts that it is counted only as one approach. Thus, by definition, each interaction event could yield at most one directed approach. Cases in which both animals moved toward each other are included only if the movement was asymmetric (i.e., exceeded the ratio threshold). If both animals approached to a similar extent, the event was excluded from the directed-approach analysis.

Second, we acknowledge that the originally used distance-ratio threshold (1.5) was heuristic. As discussed above, directionality was assigned only if one animal's approach toward the other exceeded the other's approach by a predefined distance-ratio threshold. We revisited this threshold in the minimum ratio of approaches and examined thresholds systematically. We therefore performed a sensitivity analysis quantifying how the number of detected directed approaches varies with the distance-ratio threshold (now shown in New Supplementary Fig. 3). The tuning curve shows a steep drop at low thresholds (consistent with capturing many mutual or symmetric movements) and a slower decay at higher thresholds. To ensure directionality, we therefore reanalyzed the data using a more conservative threshold ratio of 3 and updated the threshold throughout the manuscript. Importantly, the main conclusions remain unchanged (new Fig. 4m-n, new Supplementary Fig. 15). Further, we

show stability across a broader range of thresholds (2.5–4.5, upper bound chosen because beyond this point the number of detected events drops below ~66% of that at the lower bound) (e.g. new Fig. 8e-f). Across this range, the increased approach activity of sRC members remains stable (new Supplementary Fig. 4, for an additional comparison of $OXTR^{\Delta AON}$ and control mice see also new Supplementary Fig. 3).

We added to the main Results starting L. 219: *"Secondly, to assure directionality in the approach, the distance travelled by the 'approaching' mouse in the 4s before the interaction had to be at least 3 times the distance travelled by the other mouse (new Fig. 4f). The threshold was chosen based on a sensitivity analysis (new Supplementary Figs. 3-4). Events below this relative directionality threshold were excluded from directed-approach analyses. This criterion was chosen to ensure that approach events had a clear direction while retaining a sufficient number of events for the analysis (see Methods). Mutual approaches were therefore excluded, as the relative distances travelled by both interaction partners would be comparable and thus fail to meet the predefined asymmetries in approach." And in the* Methods starting L. 666: "*If the distance traveled by one mouse exceeded that of the other by a factor of 3 or more, it was classified as the approaching individual. If neither met this threshold – such as when both mice were moving in parallel or converging passively – no approach was assigned. This criterion was chosen to ensure a clear directionality in the approaches, while maintaining a sufficient number of events for the analysis. Since the precise value of this threshold could impact the results of our analysis, we controlled for the robustness of the main results of the article over a range of threshold values from 2 to 4.5 (Supplementary Fig. 3-4)."*

- The sparse inclusion of OXTRΔAON mice within mostly neurotypical groups is an interesting and ecologically relevant design. However, the current experiments lack a classic group-level control consisting of wild-type-only groups. The authors themselves explicitly argue that individual-level perturbations propagate through the social network and reshape group behavior. Thus, the presence of mutants itself becomes a group-level manipulation. Control groups without any mutants are therefore needed to establish a proper baseline for interpreting these network effects, or at a bare minimum, this limitation should be explicitly acknowledged. However, I believe compromising on this issue would significantly deplete the impact of the paper. I thus encourage the authors to think about including the conventional control groups as a baseline reference for their intriguing results.

We thank the reviewer for this important remark and agree that, because individual-level perturbations can propagate through a social network, even a minority of $OXTR^{\Delta AON}$ mice may influence group-level social dynamics. Although wild-type-only groups were not part of the original experimental design, we have now incorporated data from an unpublished experiment with four wild-type-only groups. In 3 out of 4 wild-type-only groups, we observed the emergence of a stable RC (sRC), supporting that sRC formation is not contingent on the presence of mutants. These data are now presented in a new Supplementary Fig. 10, and we added the corresponding cohort details to the Methods (L. 556: "*Additionally, as a control group to test if similar sRCs form in groups without $OXTR^{\Delta AON}$ mice, we analyzed data from male wild-type C57BL/6J mice (obtained from Charles River, Sulzfeld); for details on this wild-type cohort see Supplementary Table S4*.") and to the animal summary table (Supplementary Table S4).

We also added a sentence in the result section starting L.263: "*We also tested if similar sRCs emerge if no interspersed mutants are present. Indeed, in a separate cohort of younger adult mice that were all wild-types, three out of four groups had sRCs (Supplementary Fig. 10, Supplementary Table S4).*" and in the discussion starting L. 405: "*The formation of sRCs was robust to insertion of a minority of $OXTR^{\Delta AON}$ mice, as supported by groups of wild-type mice that never interacted with mutants. Also, groups of either older or younger adults both generally formed sRCs."*

- The RC are central to the paper, yet their quantitative definition is not clearly stated in the Results. The criteria used to identify RCs should be briefly but explicitly defined here, not only put in the Methods, for readers' benefit.

We had originally moved methodological points to the methods section to fit the formatting guidelines. We now re-integrate the most important details into the results section to bring more clarity. Specifically, we added in the results section starting L.249: *"We defined RC membership based on node degree in the pruned interaction network. Nodes with degree $d \geq 3$ were classified as RC members. Prior to computing degree, we pruned weak connections by retaining, for each node, edges to its mutual k-nearest neighbors (k = 3; see Methods). We confirmed that the main conclusions are stable when varying the pruning criterion (see Supplementary Fig. 8)."*

- For Fig. 4, it would be more informative to present 4 experimental groups rather than 3: RC controls, non-RC controls, RC OXTRΔAON, and non-RC OXTRΔAON. This would allow a clearer separation of network/genotype effects. The number of OXTRΔAON mice in the experiments may be too small for such stratification, but if so, this should be openly stated. This has a significant impact on the interpretation of the results, and it is unclear to me why the authors decided not to present the data in this way from the beginning. If there is an justification of that fact it should be presented to the reader.

We thank the reviewer for this suggestion and agree that, in principle, separating the data into four groups (sRC vs non-sRC × genotype) would disentangle network position and genotype. However, in our dataset only 2 OXTR$^{\Delta AON}$ cases (across group configurations/rounds included in this analysis) were classified as belonging to an sRC. This case is too small for a meaningful statistical comparison with a separate "sRC OXTR$^{\Delta AON}$" subgroup, and therefore not presented as four groups in the original submission. To make this transparent, we now explicitly state in the Results that only two OXTR$^{\Delta AON}$ observations fell into the sRC category (L. 328 onwards "*Indeed, across all groups, only two OXTR$^{\Delta AON}$ mice entered the sRC, a frequency (6.4%) considerably below the fraction observed in control mice (30.3%) (new Fig. 7a).*").

We also revised the figure presentation to improve readability: Fig. 4 has been updated and is now shown as new Fig. 5e–h, using a simplified layout that avoids an essentially empty "sRC OXTR$^{\Delta AON}$" group. Additionally, we decided to replace the total interaction time (in minutes) by the average interaction duration (in seconds) in the new Figs. 4l & 5f, as we felt that it is a more informative metric of behavior.

Finally, to address the reviewer's request for clearer genotype separation where statistical power is adequate, we now report an additional post hoc comparison of OXTR$^{\Delta AON}$ vs control within the non-sRC subgroup in the Results (L. 272): "Notably, OXTR$^{\Delta AON}$ mice behaved similarly to non-sRC-member control mice on these measures, as similar results were obtained with the different measures (Supplementary Figure 11 and Supplementary Table S3)."

- Line 259: "sRC members tended to occupy higher ranks in the social hierarchy, which was quantified by incidental tube-test competitions within the NoSeMaze (Fig. 6d-e, 260 Supplementary Fig. 12b). This cannot be unequivocally concluded given the applied data presentation; the notion requires additional statistical support.

We thank the reviewer for this point and now provide the statistical support for the statement in the results starting L. 293: "*Membership in sRCs was associated with differences in other social behaviors. Using incidental competitions in the integrated tube test of the NoSeMaze, we quantified ranks in the social hierarchy (Fig. 6d). Non-members had a relatively uniform rank distribution, with no significant deviation from uniformity [χ^2 goodness-of-fit test, $p = 0.11$] (Fig. 6e). Importantly, sRC members occupied higher social ranks compared to non-sRC-members (Fig. 6e). Consistent with social rank being largely internalized and relatively stable across different groups [37], the distribution of social ranks of mice that were in the sRC during one NoSeMaze round did not significantly differ from the rank distribution in*

other rounds when they were not in an sRC (Fig. 6f). Together, this suggests that higher ranks facilitate, but are not sufficient for sRC entry.

We also tested for proactive chasing of others through the tubes (Fig. 6g). sRC-members initiated significantly more chases than non-sRC-members (two-sided t-test, p = 1.01e-12, Fig. 6h). Mice that were in the sRC during one NoSeMaze round contribute more to chasing than in rounds when they were not part of the sRC (Fig. 6i, see also Supplementary Fig. 13b and Supplementary Fig. 14). The sRC-members initiated chasing toward both fellow clique members and non-members (Supplementary Fig. 13e). The behavior of OXTR$^{\Delta AON}$ mice in chasing and social rank resembled largely those of non-sRC-member control mice (Supplementary Fig. 13a,c-d).“

We also moved the relevant panel from the Supplementary material into the main manuscript (new Fig. 6h) and added the corresponding statistical test (two-sided t-test, p = 0.004) demonstrating that the rank distribution of sRC members differs from that of non-members. We also revised the Results paragraph to follow the order of new Fig. 6 and to state the conclusion in a way that matches the statistical evidence.

- The conclusion that OXTRΔAON mice fail to enter RCs seems to be difficult to evaluate, given the low number of the mutant animals overall in comparison to the WT mice. I would recommend that the authors additionally support this claim by reporting and comparing the probability of RC membership for (i) the general tested population, (ii) wild-type mice, and (iii) mutant mice. Not only the comparison between the general population/wt and the mutant mice, which I reckon is the analysis right now (Fig. 6a): I am unsure which one it is, since the presentation of this result is challenging for the reader, and the description is insufficiently clear for me to be more definite. A more explicit description would greatly benefit the comprehensibility of these data. In my view, without this, it remains unclear whether the apparent exclusion of the OXTRΔAON mice reflects a biological effect or simply the sparse representation of mutants. This is another case where including or referencing wild-type-only control groups would greatly help disentangle genotype effects from group-composition effects. Especially given the conclusions of the authors, such as “the overall strength of outgoing approaches fluctuated to a similar degree in mut and wt mice (line 310)”.

When revising the ms. we also noticed that the presentation of the results did not visualize the magnitude of the effect with the precise group sizes and composition being separately presented in the supplement.

We added in the result section now the probability of a control mouse and an OXTR$^{\Delta AON}$ mouse to be in an sRC starting in L. 328: “*Indeed, across all groups, only two OXTR$^{\Delta AON}$ mice entered the sRC, a frequency (6.4%) considerably below the fraction observed in control mice (30.3%) (Fig. 7a).*“

We also added a pie chart better visualizing these probabilities in the total sample to new Fig. 7A displaying clearly the proportions of RC controls, non-RC controls, RC OXTR$^{\Delta AON}$, and non-RC OXTR$^{\Delta AON}$, as well as their exact numbers.

- Also, using NEF as the measure of social stability is an exciting concept, and I believe showing its application in the manuscript more broadly and explicitly would be beneficial to the field. Additionally, it might be interesting to the reader what the effects of applying this analytical approach to other measures of sociability, such as the time series of the time spent together in subsequent bins by the mice of diverse genotypes (since the OXTRΔAON mice do not differ from the wild-types when this variable is taken under consideration as a scalar value).

We thank the reviewer for the comment. Following this suggestion, we applied the NEF to other measures, and found significant differences for interactions and total time spent interacting. We consequently added these results to new Supplementary Figs. 17-18, and mentioned them in the main text starting L.373: “Interestingly, these erratic interactions patterns translated to a diminished

consistency of the time spent interacting over the duration of the experiment (Supplementary Fig. 17-18)."

Further, we also revised the effect of outliers ("bursts" of activity) on the NEF. We found that normalizing edge-wise fluctuations by the median daily activity, rather than the mean, improves robustness because occasional outlier days can inflate the mean and thereby suppress NEF values. For the same reason, we replaced the standard deviation term (computed around the mean) with the mean absolute deviation about the median (Boos, 2004, Habib 2012), which is less sensitive to sporadic spikes in activity. These changes preserve the interpretation of the NEF as fluctuations relative to typical activity levels, while improving robustness. We updated the NEF definition accordingly in the Methods and recomputed the corresponding results (new Fig. 8). Note that other robust fluctuation estimators can be used in the numerator of the NEF, producing similar results (see new Supplementary Fig 19).

In new Fig. 8e-f, we now additionally provide tuning curves showing that NEF-related statistical conclusions remain stable across a range of approach-asymmetry thresholds (shown separately for ingoing and outgoing directions; detailed also in the Methods starting L. 841: "*To ensure that the statistical conclusions on the approach behavior drawn from the NEF remain stable across a range of approach-asymmetry thresholds, we evaluate the NEF across a range of relative directionality threshold values (see new Fig. 8e-f). To gain a sense of effect sizes, we display the difference between the mean of the distribution of the NEF's value of OXTR$^{\Delta AON}$ mice and control mice, as a function of the relative directionality threshold parameter (shown as the gray $\mu_{OXTR\Delta AON}$-$\mu_{control}$ line in new Fig. 8e-f).*" and the figure caption to new Fig. 8e-f: "*Tuning curves showing how the differences in NEF for e) the outgoing approaches and f) incoming approaches of OXTR$^{\Delta AON}$ mice compared to controls change as a function of the approach distance threshold. The black curves show the p-value of the differences, while the gray curves show the average of the distribution of the NEF for OXTR$^{\Delta AON}$ mice minus the average of the distribution of control mice ($\mu_{OXTR\Delta AON} - \mu_{controls}$). These show that the effect size is maximal at threshold values around 3, as well as the p-value of the comparison between outgoing approaches of OXTR$^{\Delta AON}$ and control mice.*").

Finally, we removed the burstiness metric because it lacked sufficient robustness when probing it on different thresholds and pruning parameters.

- The Discussion content (lines 397-421) should be rethought and rephrased as needed, if the conclusions regarding OXTRΔAON mice's ability to enter RCs is seen in a more comprehensive way in the light of new analysis (if performed).

As detailed above in the point-by-point reply, we discuss the new results.

MINOR:

- The claim (line 69) that difficulties in forming stable relationships become particularly relevant in adulthood is problematic. In ASD, the most severe functional impairments typically emerge in early childhood, when individuals learn to operate in structured social environments such as kindergarten and school; the current framing appears driven by the authors' adult mouse model rather than by clinical reality. This statement should either be removed or explicitly reframed/cited to explain how adult de novo social integration deficits relate to early developmental social dysfunction.

We rewrote this point so that the point is not misunderstood starting L. 69: "*Symptoms may however not fully manifest until the increasing complexity and flexibility in social demands exceed limited capacities [11].*". This algins with DSM-V and our clinical observations.

- The intro paragraph (from line 91) already provides a nuanced account of oxytocin's role in social behavior. To further strengthen it, I would recommend that the authors explicitly add (and cite) that

oxytocin can promote not only affiliation but also aggression and other context-dependent "unpleasant" social responses, depending on the situation. Making this explicit would reinforce oxytocin as a complex, state-setting neuromodulator shaping multiple forms of social behavior, which aligns directly with the network-level framework of the paper.

We incorporated this point in L. 104: "*Oxytocin modulates diverse region-specific brain functions, including social sensory processing, motivation, bonding, and aggression [20–23, 28].*"

- While dyadic assays (Fig. 1) are valuable for establishing baseline social interactions, dyadic behaviors outside a group are not equivalent to dyadic behaviors embedded in a social network (as the authors themselves argue). A brief acknowledgment of this in the Discussion would strengthen the interpretation of the following group-level results.

We thank the Reviewer for highlighting this point. We plan a study that compares simple standardized tests outside of the NoSeMaze versus behaviors in the NoSeMaze accounting for the multiple factors that need to be considered for a valid comparison. We stated more clearly the complementary function of the simple dyadic test in the discussion (starting L. 440): *"The consequences of impaired state induction in mice carrying OXTRΔAON were surprisingly selective in the complex social environment. They kept normal rates of social interactions in the NoSeMaze. Also, the detailed patterns of olfactory social sniffing behavior were normal in OXTR$^{\Delta AON}$ mice during microsequences in simple dyadic tests; supporting together that motivation to interact or sample others are mediated by other OXT-modulated brain circuits.*

- Fig. 2b and c would benefit from being more explicit about the number of reshuffling rounds and how they were organized. In particular, the depiction of different age groups in the figure is not easily mapped onto the description in the Results. Aligning the figure more closely with the text and explaining the reshuffling structure more clearly would substantially improve readability.

We thank the reviewer for pointing out that the NoSeMaze study design (age cohorts and repetitions) should be made easier to follow. We have clarified the experimental repetition structure in the Results/Methods text. In addition, we updated former Fig. 2 (new Figure 3) to explicitly summarize the two age-defined cohorts and to indicate the number of NoSeMaze group rounds per cohort, as well as the reshuffling procedure between rounds. Full group compositions, reshuffling procedures, and data availability are provided in Supplementary Tables S1–S5.

We revised the results section starting L. 181: *"To assess social interaction dynamics in a complex setting while retaining full tracking of individual behavior, we employed the NoSeMaze system [37]. The NoSeMaze provides a sensor-rich environment enabling long-term, automated tracking of cognitive and social behaviors in group-housed mice without human interference (new Fig. 3a). We monitored groups of 9–10 RFID-chipped male mice (79 total; 22 OXTR$^{\Delta AON}$, 57 controls) on a C57BL/6J background over continuous three-week rounds in the NoSeMaze. Each group included two to five OXTR$^{\Delta AON}$ mice interspersed among controls to mimic sparse genetic variation. To ensure that phenotypes were observed across the adult lifespan, we studied two age cohorts separately: younger (16–30 weeks; 41 mice, 16 OXTR$^{\Delta AON}$, 25 controls) and older adults (55–97 weeks; 38 mice, 10 OXTR$^{\Delta AON}$, 28 controls) (new Fig. 3b). Before entering the NoSeMaze, mice within each cohort were repeatedly reshuffled in home-cage groups of 3-5 animals (new Fig. 3c-d) to promote familiarity among future group members. This procedure may also have contributed to the lack of overt aggression or injuries in the NoSeMaze. Most mice participated in multiple rounds (see Supplementary Table S2). To assess whether social behaviors depended on specific group composition, mice lived with different group members in each round of the NoSeMaze ('reshuffling', new Fig. 3d).*

We then established a pipeline to extract the social interaction networks from continuous video data in the open field arena for the groups of individually identified mice, including interspersed OXTR$^{\Delta AON}$ mutants (Fig. 4a-b). To identify individual mice, distinct fur patterns were bleached onto the back of the mice before each round (Fig. 4c). We analyzed the first 15 days of each round, as the subsequent

regrowth of the fur compromised the reliability of pattern recognition by the trained Convolutional Neural Network (CNN). Before each new round, the fur patterns were refreshed. 16 out of 21 NoSeMaze rounds with interspersed OXTR$^{\Delta AON}$ mice yielded video datasets of sufficient quality for social network reconstruction and were included in the video-based analyses (Fig. 4a-b). Additionally, we analyzed four rounds with wild-type mice only (cf. Supplementary Fig. 10), totaling overall to 7,200 hours of continuous video data used to generate the social networks in the final dataset (Fig. 4d)."

We revised new Fig. 3 (former Fig. 2) together with its caption: ***"Fig. 3: … b)*** *Overview of the two adult cohorts studied in separate experimental series. Cohort 1 ("younger adults") comprised 41 male C57BL/6J-background mice (16 OXTR$^{\Delta AON}$, 25 controls), aged 16–30 weeks during NoSeMaze testing, and contributed 11 NoSeMaze group rounds in total. Cohort 2 ("older adults") comprised 38 male mice (10 OXTR$^{\Delta AON}$, 28 controls), aged 55–97 weeks during NoSeMaze testing, and contributed 10 NoSeMaze group rounds in total.* ***c)*** *Experimental timeline. Before entry into the NoSeMaze, home-cage compositions were repeatedly reshuffled every 3–4 days (3–5 mice per cage) to promote familiarity among cohort members. Mice were then housed in the NoSeMaze for approximately 3 weeks per round in groups of 9–10, with 2–4 groups run in parallel. Between consecutive NoSeMaze rounds, mice returned to reshuffled home cages, where they were again reshuffled in the last week before the next NoSeMaze round. Reshuffling occurred within cohorts only, not across cohorts.* ***d)*** *Sankey-style diagram exemplarily illustrating how mice were reassigned across successive NoSeMaze rounds with reshuffling periods in home cages to ensure broad familiarity both before and between rounds. Each group consisted of 9–10 individuals with 2–5 OXTR$^{\Delta AON}$ mice (red circle). Reshuffling enabled dissociation of effects emerging from internalized traits in individuals versus group composition context.*

NoSeMaze, Non-invasive Sensor-rich Maze; RFID, radio-frequency identification"

- In Fig. 3, the rationale for giving panels g and h such prominent space is unclear. It would help the reader if the authors either justify their centrality or reduce it. Similarly, i and j seem to reiterate the same point, however, from slightly different angles. In my view one should be put in the supplement; k is unclear on when the dark/light phase begins; also word "massive" in the figure caption seems obsolete.

In former Fig. 3 (now new Fig. 4), we moved, as suggested, the mean results (old Fig. 3j) to Supplementary Fig. 2, leaving panel 3i (now Fig. 4i) in the main figure as it is closer to the raw data.

After internal discussion, we have decided to retain both 3g and 3h (now new Fig. 4g/h), as we wish to explicitly show the distinction between undirected networks (e.g., interactions) and directed networks (e.g., approaches). This is an important aspect of our analysis, and we feel that showing it in the figure facilitates comprehension.

Additionally, we have updated old Fig. 3k (now new Fig. 4j) by adding a line plus shading to make the dark cycle explicit. Also, we replaced the term "massive" in the caption with "long-term".

- In Fig. 4, panels a and c are difficult to read in their current form. Presenting these panels as higher-quality graphics would substantially improve their interpretability and make the structure of the RC easier for the reader to follow

We reworked the figures (now in Fig. 5) to improve readability of the graphs (font sizes etc). We also replotted old Fig. 4c (now Fig. 5b) to visualize the relevant network structures better.

- This claim (line 228) "sRC emerged in all 10 rounds with older adults (Fig. 4c, Supplementary Fig. 7), while in younger adults they were observed in 4 of 7 rounds (Supplementary Fig. 7), potentially suggesting that age may favor clique formation." lacks the experimental support due to the insufficiency of the control conditions performed; I would advise softening it and further elaborating on it in the discussion.

Even though we deal with a large data set, the number of rounds of groups does not allow for a definitive statistical conclusion regarding age-related differences in sRC formation. The corresponding result section now only states (starting in L. 261): "*sRCs emerged in all 10 rounds with older adults and in 4 out of 7 rounds in younger adults (new Fig. 5b, Supplementary Fig. 6).*" and in the discussion in L. 407-408: "Also, groups of either older or younger adults both generally formed sRC."

- Chasing behavior in semi-naturalistic settings has been shown to correlate with social hierarchy/dominance (e.g., Winiarski et al., 2025). Given this, the way data is interpreted here should be more carefully contextualized. If the present results diverge from that established relationship, this discrepancy should be discussed.

We added a section on this point in the discussion starting L. 413: "*In these groups, dominance in tube competitions and chasing have nuanced functions [37]. The hierarchy measured with incidental tube competitions indexed a stable internalized social rank carried by individuals across different group compositions. In many cases, chasing functions in these groups as an active mechanism of social rank negotiation within the upper tier of the hierarchy [37]. It should be noted however that the these social behaviors and their functions may change in small groups, other environments that foster territorial behavior, or limited access to resources [35, 38–40].*"

- Fig. 6f: should be "unstable", not "instable"; also, Fig. 6 f-g should rather be the initial panels in this data illustration than shown at the end.

We corrected this in the revised figure and rearranged and split this final figure (now new Fig. 7 and 8).

- This notion "(…) our findings support a non-hierarchical view of social brain functions, in which selective deficits in higher-order social functions can arise from alterations in early sensory processing rather than from dysfunction in higher brain regions. " presented in the discussion (line 393), and strongly supported by the presented data should be described in more depth, as it represents one of the most noteworthy discoveries presented in the paper.

We thank for highlighting this point and embedded this in a larger context: L. 454: "*Our findings provide experimental evidence that alterations in early sensory processing and recognition mechanisms can propagate to higher-order functions, extending this principle to the navigation of social networks. In doing so, they complement prior work demonstrating that sensory atypicalities constrain the construction and navigation of internal models of the environment, both social and nonsocial [41–43].*"

Congratulations to the Authors on this intriguing and well-executed study. I am grateful for the opportunity to contribute to the improvement of this work. I remain at your disposal, as I believe this is a valuable and timely contribution to the field of social neuroscience. Alicja Puścian

We thank you for the encouraging feedback.

References:

Anderson, K.A., Sosnowy, C., Kuo, A.A., Shattuck, P.T., 2018. Transition of Individuals With Autism to Adulthood: A Review of Qualitative Studies. Pediatrics 141, S318–S327. https://doi.org/10.1542/peds.2016-4300I

Ashbaugh, K., Koegel, R., Koegel, L., 2017. Increasing Social Integration for College Students with Autism Spectrum Disorder. Behav. Dev. Bull. 22, 183–196. https://doi.org/10.1037/bdb0000057

Barendse, E.M., Hendriks, M.P.H., Thoonen, G., Aldenkamp, A.P., Kessels, R.P.C., 2018. Social behaviour and social cognition in high-functioning adolescents with autism spectrum disorder (ASD): two sides of the same coin? Cogn. Process. 19, 545–555. https://doi.org/10.1007/s10339-018-0866-5

Butler, P.D., Javitt, D.C., 2005. Early-stage visual processing deficits in schizophrenia. Curr. Opin. Psychiatry 18, 151–157. https://doi.org/10.1097/00001504-200503000-00008

Friston, K., 2010. The free-energy principle: a unified brain theory? Nat. Rev. Neurosci. 11, 127–138. https://doi.org/10.1038/nrn2787

Fuentes, J., Hervás, A., Howlin, P., (ESCAP ASD Working Party), 2021. ESCAP practice guidance for autism: a summary of evidence-based recommendations for diagnosis and treatment. Eur. Child Adolesc. Psychiatry 30, 961–984. https://doi.org/10.1007/s00787-020-01587-4

Gurbuz, E., Hanley, M., Riby, D.M., 2019. University Students with Autism: The Social and Academic Experiences of University in the UK. J. Autism Dev. Disord. 49, 617–631. https://doi.org/10.1007/s10803-018-3741-4

Hodis, B., Mughal, S., Saadabadi, A., 2025. Autism Spectrum Disorder, in: StatPearls. StatPearls Publishing, Treasure Island (FL).

Pellicano, E., Burr, D., 2012. When the world becomes ‘too real’: a Bayesian explanation of autistic perception. Trends Cogn. Sci. 16, 504–510. https://doi.org/10.1016/j.tics.2012.08.009

Seltzer, M.M., Shattuck, P., Abbeduto, L., Greenberg, J.S., 2004. Trajectory of development in adolescents and adults with autism. Ment. Retard. Dev. Disabil. Res. Rev. 10, 234–247. https://doi.org/10.1002/mrdd.20038

Shattuck, P.T., Narendorf, S.C., Cooper, B., Sterzing, P.R., Wagner, M., Taylor, J.L., 2012. Postsecondary education and employment among youth with an autism spectrum disorder. Pediatrics 129, 1042–1049. https://doi.org/10.1542/peds.2011-2864

Shattuck, P.T., Orsmond, G.I., Wagner, M., Cooper, B.P., 2011. Participation in Social Activities among Adolescents with an Autism Spectrum Disorder. PLOS ONE 6, e27176. https://doi.org/10.1371/journal.pone.0027176

White, S.W., Oswald, D., Ollendick, T., Scahill, L., 2009. Anxiety in children and adolescents with autism spectrum disorders. Clin. Psychol. Rev. 29, 216–229. https://doi.org/10.1016/j.cpr.2009.01.003

Habib, E. A. (2012). Mean absolute deviation about median as a tool of explanatory data analysis. *International Journal of Research and Reviews in Applied Sciences*, *11*(3), 517-523.

Boos, Dennis D., and Cavell Brownie. "Comparing variances and other measures of dispersion." (2004): 571-578.

Point-by-point reply:

Reviewer #1 (Remarks to the Author):

All comments and concerns have been addressed in the revisions. I recommend publishing the article.

We thank the Reviewer for his helpful suggestions that substantially improved the manuscript.

Reviewer #2 (Remarks to the Author):

I believe that the authors have done a very good job addressing the comments. The new version of the ms is significantly clearer. Several of the corrections made the work stronger. Especially in terms of the presented rationale and the logic of the analyses.
Most notably, the authors improved the description of the algorithm used to quantify approach behavior, which lies at the foundation of their analysis of complex social behavior. This is an important correction. In the revised version, this measure is much more rigorous and clearly explained, which increases confidence in the analyses built on it.
Figures have also been significantly improved. Especially new figs. 3 and 5. They are now much more reader-friendly and make the logic of the results easier to follow. In addition, the authors performed new quantitative analyses, including new statistical testing, which further strengthens the conclusions.
The authors also improved the normalized edge fluctuation (NEF) calculations by changing the normalization strategy so that it better reflects behavioral fluctuations relative to the typical (rather than mean) activity levels of the animals. I see this as a significant improvement. The authors also applied it to new measures of social behavior in their experiments, which brought new insights, now presented in the Suppl figs. 17-19. NEF is, in my opinion, an exciting measure of variability that could be further applied to other experimental measures of sociability in the network context, beyond this work.
Further, the addition of new control experiments with WT mice was an indispensable element for empowering the conclusions of the paper. The fact that RCs were formed in 3 out of 4 cohorts is informative. It shows that the process is not universal, which is completely fine, as the social nature of the groups is understandably complex. However, I strongly believe that these results are critical to the understanding of the paper and should not remain in the supplementary material and cited in the main ms, which is how the authors decided to place it. They should be moved to the main figures. In my view, this is not a secondary control, but one of the main results on which the whole logic of the paper is built. I would also advise the authors to include in these fig. panels analogous to those currently shown in fig. 5e-h, to make the comparisons equivalent. In addition, a pie chart equivalent to the new (and very clear!) fig. 7a, but prepared for the control experiments with WT animals exclusively, would be a greatly valuable addition to this data presentation. Taken together, this would further help disentangle group effects from genotype effects, which I think is critically important, especially in face of inability to perform statistical tests allowing to disentangle the two, because of very few mutant animals in the RCs.
Provided this correction is made, I recommend this paper for publication. I believe it is a very valuable and well-thought-out addition to our understanding of higher order group processes in rodent social groups. I congratulate the authors on the immense workload they put into this paper, as the effects of their work are highly noteworthy.

We thank the Reviewer for her careful assessment and valuable suggestions that strengthened the ms.

Following Reviewer's first point, we have moved the figure presenting the control experiment from the Supplementary Information to the main manuscript (now Fig. 5). We have also revised the corresponding results section to integrate it better into the overall narrative of the paper. All changes are highlighted in red starting l. 244: "*Interaction data were analyzed across five consecutive three-day windows per round. Given the high connectivity in the networks, graphs were pruned using a mutual nearest-neighbor approach to isolate behaviorally relevant substructures (Fig. 5a). These structures correspond to rich-clubs (RC) [15, 22]. We defined RC membership based on node degree in the pruned interaction network. Nodes with degree* $d \geq 3$ *were classified as RC members. Prior to computing degree, we pruned weak connections by retaining, for each node, edges to its* ***mutual k-nearest neighbors*** *(k = 3; see Methods).*

Before analyzing the main cohort containing both controls and OXTR$^{\Delta AON}$ mice, we first asked whether RCs were expected to emerge in pure groups of wild-type mice of the same strain as the main cohort (see Supplementary Table S4). To address this, we analyzed four independent rounds consisting exclusively of younger adult male wild-type mice. We were particularly interested if stable rich-clubs (sRC) emerged in these groups. RCs were considered stable if they persisted across at least 4 out of 5 three-day-graphs. Such sRCs were identified in three of four groups (Fig. 5b).

We then examined the main cohort with interspersed OXTR$^{\Delta AON}$ mice (Fig. 6a, Supplementary Fig. 6). In most groups (81%), we observed sRCs. sRCs emerged in all 10 rounds with older adults and in 4 out of 7 rounds in younger adults (Fig. 6b). Probabilistic modeling confirmed that such persistence was highly unlikely to occur by chance ($p < 10^{-7}$ for pairs; $p < 10^{-9}$ for triads), indicating genuine social structure. This finding was further supported by richness coefficients consistently exceeding random chance throughout the experiment (see Supplementary Fig. 7). Finally, the overall structures of sRCs were stable when varying the pruning criterion (see Supplementary Fig. 8).

Notably, sRCs were robustly identifiable in both undirected interaction and directed approach networks (Supplementary Fig. 9). sRC members displayed distinct interaction patterns. sRC members exhibited greater reciprocity: 96.0% of their approaches were reciprocal (Fig. 6c), compared to 66.2% in the whole population (Fig. 6d). Compared to non-sRC-members, they engaged in more interactions and approaches, although mean interaction duration did not differ (Fig. 6e-h). Notably, OXTR$^{\Delta AON}$ mice behaved similarly to non-sRC-member control mice on these measures, as similar results were obtained with the different measures (Supplementary Figure 10 and Supplementary Table S3). Taken together, the formation of sRCs is a typical feature in the habitat and can be identified both from undirected interaction graphs and directional approach networks."

The proof of principle cohort served to show that stable rich clubs also form in pure wildtype cohorts. It was therefore not designed and powered to replicate the statistical investigation of approach and interaction quantities (like in Fig. 5e-h (now Fig. 6)) also on the smaller proof-of-principle cohort. Specifically, the proof-of-principle wild-type cohort comprises 4 rounds compared to 16 rounds in the main cohort, and thus also a smaller number of mouse subjects. Displaying these statistical tests on the proof-of-principle cohort would therefore not be statistically sound. Nonetheless, we checked and the results are consistent with the main cohort. We are currently preparing other large data sets for other questions using the NoSeMaze. These data will also comprise pure wild-type cohorts where these tests can be rigorously performed with sufficient power.
